# *In Vitro* Ischemia Triggers a Transcriptional Response to Down-Regulate Synaptic Proteins in Hippocampal Neurons

**DOI:** 10.1371/journal.pone.0099958

**Published:** 2014-06-24

**Authors:** Joana Fernandes, Marta Vieira, Laura Carreto, Manuel A. S. Santos, Carlos B. Duarte, Ana Luísa Carvalho, Armanda E. Santos

**Affiliations:** 1 CNC-Center for Neuroscience and Cell Biology, University of Coimbra, Coimbra, Portugal; 2 Faculty of Pharmacy, University of Coimbra, Coimbra, Portugal; 3 RNA Biology Laboratory, Department of Biology and CESAM, University of Aveiro, Aveiro, Portugal; 4 Department of Life Sciences, Faculty of Sciences and Technology, University of Coimbra, Coimbra, Portugal; University of Naples Federico II, Italy

## Abstract

Transient global cerebral ischemia induces profound changes in the transcriptome of brain cells, which is partially associated with the induction or repression of genes that influence the ischemic response. However, the mechanisms responsible for the selective vulnerability of hippocampal neurons to global ischemia remain to be clarified. To identify molecular changes elicited by ischemic insults, we subjected hippocampal primary cultures to oxygen-glucose deprivation (OGD), an *in vitro* model for global ischemia that resulted in delayed neuronal death with an excitotoxic component. To investigate changes in the transcriptome of hippocampal neurons submitted to OGD, total RNA was extracted at early (7 h) and delayed (24 h) time points after OGD and used in a whole-genome RNA microarray. We observed that at 7 h after OGD there was a general repression of genes, whereas at 24 h there was a general induction of gene expression. Genes related with functions such as transcription and RNA biosynthesis were highly regulated at both periods of incubation after OGD, confirming that the response to ischemia is a dynamic and coordinated process. Our analysis showed that genes for synaptic proteins, such as those encoding for PICK1, GRIP1, TARPγ3, calsyntenin-2/3, SAPAP2 and SNAP-25, were down-regulated after OGD. Additionally, OGD decreased the mRNA and protein expression levels of the GluA1 AMPA receptor subunit as well as the GluN2A and GluN2B subunits of NMDA receptors, but increased the mRNA expression of the GluN3A subunit, thus altering the composition of ionotropic glutamate receptors in hippocampal neurons. Together, our results present the expression profile elicited by *in vitro* ischemia in hippocampal neurons, and indicate that OGD activates a transcriptional program leading to down-regulation in the expression of genes coding for synaptic proteins, suggesting that the synaptic proteome may change after ischemia.

## Introduction

Global cerebral ischemia is a pathological condition in which brain tissue is subjected to reduced levels of oxygen and glucose due to impairment in blood supply to the entire brain, causing biochemical modifications in the normal functioning of neurons that can lead to injury in specific neuronal subpopulations. One of the main features of transient global cerebral ischemia is the delayed death of the pyramidal neurons of the CA1 region of the hippocampus, which occurs hours to days after the insult. This time-window between the end of the transient ischemic insult and the first signs of neuronal demise is believed to be associated with the activation of competing programs of gene expression, in which some will facilitate cell survival, whereas others will contribute to neuronal death [1].

A great effort has been put into identifying genes that participate in the response of hippocampal cells to global cerebral ischemia *in vivo* [2]–[4]. However, while it is accepted that several cellular functions are compromised, our understanding of how this correlates with the selective and delayed death of hippocampal neurons is still unclear. This analysis requires investigations at the molecular level, more easily performed using *in vitro* models. In the present study we used microarray technology to identify genes whose expression is significantly altered in hippocampal neuronal cultures submitted to oxygen and glucose deprivation (OGD), an established *in vitro* model for cerebral global ischemia [5]. To the best of our knowledge no large scale study was developed so far using an OGD insult as a tool to study ischemia-induced changes in the transcriptome of hippocampal neurons at different periods of recovery. In accordance to what has been previously observed in models of global and focal ischemia [2]–[4], [6]–[9], OGD induced changes in the expression levels of genes related with a variety of functions within neurons, such as the synapse. Failure in synaptic activity is one of the earliest events in cerebral ischemia, due to the energetic imbalance that occurs during an ischemic insult and that leads to neuronal depolarization and impaired neurotransmission [10]. Moreover, there are biochemical alterations that occur in neurons submitted to ischemic insults, which include changes in the expression levels and the molecular composition of proteins related with synaptic transmission, such as the ionotropic glutamate receptors of the α-amino-3-hydroxy-5-methyl-4-isoxazolepropionic acid (AMPAR) and the N-methyl-D-aspartate (NMDAR) types, among other proteins [11]–[14] that can be implicated in the mechanisms promoting either cell death or cell survival.

In particular, we observed that OGD up-regulates REST expression, triggers a transcriptional program that down-regulates synaptic protein-encoding genes and induces changes in the subunit composition of the AMPAR and the NMDAR subtypes. The extent to which the post-ischemic alterations identified in this work influence the fate of neuronal cells exposed to ischemia can now be addressed, and may result in the identification of attractive therapeutic targets for the treatment of cerebral ischemia.

## Methods

### Primary hippocampal neuronal cultures

Primary cultures of rat hippocampal neurons were prepared from the hippocampi of E18-E19 Wistar rat embryos, after treatment with trypsin (0.06%, 15 min, 37°C; Gibco Invitrogen, Paisley, UK) in Ca^2+^- and Mg^2+^-free Hank's balanced salt solution (HBSS; in mM: 5.36 KCl, 0.44 KH_2_PO_4_, 137 NaCl, 4.16 NaHCO_3_, 0.34 Na_2_HPO _4_.2H_2_O, 5 glucose, 1 sodium pyruvate, 10 HEPES and 0.001% phenol red). The hippocampi were then washed with HBSS containing 10% fetal bovine serum (Gibco Invitrogen), to stop trypsin activity, and transferred to Neurobasal medium (Gibco, Life Technologies, Paisley, UK), supplemented with SM1 neuronal supplement (1∶50 dilution; Stem Cell Technologies, Grenoble, France), 25 µM glutamate, 0.5 mM glutamine and 0.12 mg/ml gentamycin (Sigma-Aldrich, St Louis, MO). The cells were dissociated in this solution and were then plated in 6-well microplates (MW6) (for Western blot and real-time PCR experiments) or 24-well microplates (MW24) (for cell death assays), previously coated with poly-D-lysine (0.1 mg/mL, Sigma-Aldrich), or on poly-D-lysine coated glass coverslips, at a density of 85.0×10^3^ cells/cm^2^. The cultures were maintained in a humidified incubator with 5% CO_2_/95% air, at 37°C, for 14–15 days. According to what has been previously observed in our lab [15], we estimate that hippocampal cultures contain ∼10% of glial cells. All animal procedures were reviewed and approved by DGAV, Portugal.

### Oxygen-Glucose Deprivation (OGD)

For the OGD insult, hippocampal cultures were incubated in a glucose–free saline buffer (in mM: 10 HEPES, 116 NaCl, 5.4 KCl, 0.8 MgSO_4_, 1 NaH_2_PO_4_, 1.8 CaCl_2_, 25 NaHCO_3_, 25 sucrose, pH 7.3) in an anaerobic chamber (Thermo Forma 1029, Thermo Fisher Scientific, Waltham, MA), at 37 °C, for the indicated periods of time. Control neurons were placed in a similar saline buffer with 25 mM glucose instead of sucrose and kept in an air/CO_2_ incubator, at 37°C, for the same period of time. After the stimulation periods, the saline buffers were replaced by the conditioned medium and the cultures returned to the air/CO_2_ incubator, where they were left to recover for the indicated times.

In studies performed in the presence of the NMDA receptor antagonist MK-801 (10 µM), the AMPA receptor antagonist GYKI 52466 (50 µM) and the selective Ca^2+^-permeable AMPARs (CP-AMPARs) antagonist Naspm (50 µM), a pre-incubation of 15 minutes was done and the antagonists were present during both the insult and the post-ischemic period. MK-801, GYKI 52466 and Naspm were purchased from Tocris Bioscience (Bristol, UK). In the studies performed in the presence of the calpain inhibitor MDL 28170 (50 µM, Calbiochem, Darmstadt, Germany), a pre-incubation of 30 minutes was done and the inhibitor was present during both the insult and the post-ischemic period.

### Analysis of the nuclear morphology

For analysis of the nuclear morphology, neurons were fixed 24 h after OGD at room temperature in 4% sucrose/4% paraformaldehyde in phosphate-buffered saline (PBS), washed with PBS and incubated with the fluorescent dye Hoechst 33342 (1 µg/ml, Molecular Probes Europe) for 10 min. The coverslips were mounted on glass slides with Dako mounting medium (Thermo Scientific) and examined with a Zeiss Axiovert 200 fluorescence microscope (40× objective). The cell-permeable DNA stain Hoechst 33342 presents blue fluorescence. Viable cells display a normal nuclear size and a diffuse blue fluorescence, whereas damaged cells display bright blue pyknotic nuclei with condensed or fragmented chromatin [16]. The experiments were performed in duplicate and approximately 400 cells were counted per coverslip in 6–10 distinct randomly selected optical fields. Cell death is expressed as the percentage of dead cells relatively to the total number of scored cells.

### LDH release assay

For the assessment of lactate dehydrogenase (LDH) release, the culture conditioned medium was collected after the indicated times of incubation after exposure to OGD or control conditions. The LDH activity was assayed using a commercial kit (CytoTox 96 Non-Radioactive Cytotoxicity Assay, Promega, Madison, WI), and determined as indicated in the manufacturer's protocol. The percentage of LDH release was determined as the ratio between LDH activity in the extracellular medium and total LDH activity, obtained after complete cell lysis with Triton X-100. The percentage of cell death was calculated relatively to cells treated with lysis buffer, which were considered as 100%. All experiments were carried out in duplicate or triplicate, for each independent experiment.

### Total RNA isolation, RNA Quality and RNA Concentration

Seven and 24 hours after the OGD challenge, total RNA was extracted from cultured hippocampal neurons with TriZol reagent (Gibco Invitrogen), following the manufacturer's specifications. Briefly, 1 ml of TRIzol was added to each well of a 6-well plate and the content of each experimental condition (two wells) was collected. Chloroform was then added for phase separation and the RNA precipitated by isopropanol addition. The precipitated RNA was washed with 75% ethanol, centrifuged, air-dried and resuspended in 20 µl of RNase-free water (Gibco Invitrogen). RNA quality and integrity were evaluated using the Experion automated gel-electrophoresis system (Bio-Rad, Hercules, CA). RNA concentration was determined spectrophotometrically using Nanodrop 2000 (Thermo Fisher Scientific, Wilmington, DE). The samples were stored at −80°C until further use.

### Microarray hybridization

For the microarray analysis, total RNA from rat hippocampal neuronal cultures subjected to control or OGD conditions was collected after 7 h and 24 h of post-incubation in culture conditioned medium. RNA from three independent cultures was used as biological replicates. Equal amounts of RNA extract (200 ng) from each replicate were amplified and Cy-3-labeled using the Low Input Quick Amp Labeling kit (Agilent Technologies, Santa Clara, CA). Hybridizations were carried out following Agilent Technologies instructions for One-Color Microarray-Based Gene Expression Analysis (Agilent Technologies), using whole-genome Rat GE 4×44K v3 Microarrays. Images were obtained using the Agilent G2565AA microarray scanner and fluorescence quantization was performed using the Agilent Feature Extraction 10.5.1.1 software and the GE1_105_Dec08 protocol. The signal intensity was aligned and normalized between microarrays by centering the median of the signal distribution using BRB-ArrayTools v3.8.1. The microarray data was submitted to GEO database and has been given the accession number GSE54037.

### Microarray data analysis

The TIGR MultiExperiment Viewer (MeV) v4.6 was used for statistical analysis of the data. Student's *t*-test was used to determine differentially expressed genes, with a *p*-value cut-off of 0.05. Of these, only genes with a fold change above 2.0 were considered differentially expressed and included in further analyses.

For gene ontology analyses, the lists of differentially expressed genes from all conditions were imported to GoMiner. Ontological classes were selected manually and, for the production of the pie-charts, the number of genes for each class were divided by the sum of the total number of genes in the selected classes, as indicated in figure captions.

### Primer Design

Primers for target genes were designed using the “Beacon Designer 7” software (Premier Biosoft International), with the following specifications: (1) GC content about 50%; (2) Annealing temperature (Ta) between 55±5°C; (3) Secondary structures and primer-dimers were avoided; (4) Primers length between 18–24 bp; (5) Final product length between 100–200 bp. All primers used in this work are listed in **Table S1**.

### Real-Time PCR

For cDNA synthesis, 1 µg of total RNA was used with the iScript cDNA Synthesis Kit (BioRad), according to the manufacturers' instructions. For real-time PCR (qPCR) 20 µl reactions were prepared with 2 µl of 1∶10 diluted cDNA, 10 µl of 2x iQ SYBR Green Supermix (Bio-Rad) and specific primers at 250 nM. The fluorescent signal was measured after each elongation step of the PCR reaction, in the iQ5 Multicolor Real-Time PCR Detection System (Bio-Rad), and was used to determine the threshold cycle (C_t_), as previously described [17]. Melting curves were performed in order to detect non-specific amplification products, a non-template control was included in all assays, and for each set of primers a standard curve was performed to assess primer efficiency. Reactions were run in duplicate. For each gene, average C_t_ was calculated as the mean of five biological replicates for each condition. The expression level of each gene was normalized to the expression level of the gene in the corresponding control condition. All C_t_ values were normalized to two internal control genes, Actb and Gapdh (shown not to change between control and OGD conditions), using the GenEx software (MultiD Analyses). Fold change values above 1.0 indicate an up-regulation relative to the control condition, whereas fold change values below 1 indicate a down-regulation relative to the control condition. All values are indicated as log-transformed data.

### Preparation of total protein extracts

Hippocampal neuronal cultures were washed twice with ice-cold PBS before addition of ice-cold lysis buffer (in mM: 50 HEPES, 150 NaCl, 2 EGTA, 2 EDTA, 2 Na_3_VO_4_, 50 NaF, pH 7.4, with 1% Triton X-100) supplemented with 1 mM DTT and a mixture of protease inhibitors: 0.1 mM PMSF and CLAP (1 µg/ml chymostatin, 1 µg/ml leupeptin, 1 µg/ml antipain, and 1 µg/ml pepstatin (Sigma-Aldrich). Samples were frozen twice at -80°C, after which the total protein was quantified using the BCA method (Thermo Scientific). Samples were then denatured with 2x concentrated denaturing buffer (125 mM Tris, pH 6.8, 100 mM glycine, 4% SDS, 200 mM DTT, 40% glycerol, 3 mM sodium orthovanadate, and 0.01% bromophenol blue) at 95°C for 5 min.

### Biotinylation assay

Biotinylation assays were performed 24 h after the OGD insult. Cells were washed twice with PBS containing calcium and magnesium (PBS/Ca^2+^/Mg^2+^; in mM: 137 NaCl, 2.7 KCl, 1.8 KH_2_PO_4_, 10 Na_2_POH_4_, plus 0.5 MgCl_2_, 1 CaCl_2_, pH 7.4), followed by incubation with 0.25 mg/ml NHS-SS-Biotin (Thermo Scientific) for 15 min at 4°C under mild shaking. Cells were then washed twice with PBS/Ca^2+^/Mg^2+^ supplemented with glycine (100 mM) and incubated in this solution for 15 min at 4°C under mild shaking. For analysis of the surface AMPA receptors (AMPAR), cells were lysed in the lysis buffer indicated above for total protein extracts, supplemented with protease inhibitors, followed by 30 min incubation on ice, and frozen at −80°C. After thawing, cellular extracts were centrifuged at 18,000 g for 30 min at 4°C and the pellet was discarded. Fifty µg of each protein extract was used for the input and 150 µg was used for incubation with NeutrAvidin beads. For analysis of the surface NMDA receptors (NMDAR), cells were incubated with lysis buffer (in mM: 50 Tris-HCl, pH 7.4, 5 EGTA, 1 DTT), supplemented with protease inhibitors, for 30 min at 4°C under mild shaking, after which samples were collected and briefly sonicated. Cellular extracts were then incubated with 1% DOC, pH 9.0, for 1 h at 37°C, centrifuged at 18,000 g for 30 min at 4°C and the pellet was discarded. One hundred µg of each protein extract was used for the input and 400 µg was used for incubation with NeutrAvidin beads.

In both cases, NeutrAvidin beads were added in equal amounts to the supernatant fluid (2.5 µl/10 µg total protein) and incubated for 2 h at 4°C in an orbital shaker. The beads were washed four times with the correspondent lysis buffer. The samples were then eluted with 2x denaturating buffer, boiled at 95°C for 5 min and centrifuged into a tube collector with a 0.45 µm filter.

### Preparation of nuclear protein extracts

Nuclear extracts of hippocampal neurons were prepared 24 h after the OGD insult. Cells were washed with ice-cold PBS and solubilized in ice-cold buffer 1 (in mM: 10 HEPES, 10 NaCl, 3 MgCl_2_, 1 EGTA and 0.1% Triton X-100, pH 7.5) for 30–40 min. The nuclei were pelleted by centrifugation at 2,400 g for 10 min at 4°C and then resuspended in ice-cold buffer 2 (in mM: 25 HEPES, 300 NaCl, 5 MgCl_2_, 1 EGTA and 20% glycerol, pH 7.4) for 1 h, after which they were centrifuged at 12,000 g for 20 min at 4°C. The supernatants (nuclear extracts) were collected and stored at −80°C until use. Both buffers were supplemented with 0.1 mM PMSF and 1 µg/ml CLAP, as well as with 1 mM DTT before use. Protein concentration of the extracts was measured using the Bradford assay. Samples were then denatured with 2x concentrated denaturing buffer at 95°C for 5 min, and 100 µl of each sample were used for SDS-PAGE.

### SDS-PAGE and Western Blotting

Protein samples were separated by SDS-PAGE in 7.5% (REST), 8% (NMDAR subunits) or 10% (AMPAR subunits) polyacrylamide gels, transferred to PVDF membranes (Millipore, Billerica, MA) and immunoblotted. The membranes were blocked with Tris-buffered saline-Tween (TBS-T) (in mM: 20 Tris, 137 NaCl, pH 7.6, and 0.1% Tween20) with 5% non-fat milk, for 1 h at room temperature, and then incubated with the primary antibody in TBS-T 5% milk, overnight at 4°C. Incubation with antibodies against α-tubulin (Sigma-Aldrich, #T5168), β-actin (Sigma-Aldrich, #A5441) and α-spectrin (Millipore, #1622) were performed for 1 h at room temperature, whereas antibodies against REST(Millipore, #07-579), GluA1 (Millipore, #AB1504), GluA2 (Millipore, #MAB397), GluN1 (Millipore, #MAB363), GluN2A (Millipore, #AB1555P) and GluN2B (BD Biosciences, San Jose, CA, #610417) were incubated overnight at 4°C. After extensive washing, membranes were incubated with the secondary antibody conjugated with alkaline phosphatase for 1 h at room temperature. After additional washes, the membranes were developed using the enhanced chemifluorescence (ECF) substrate, and scanned on the Storm 860 Gel and Blot Imaging System (Amersham Biosciences, Buckinghamshire, UK). The density of the bands was analyzed with ImageQuant 5.0 software. For subsequent reprobing, the membranes were stripped of antibody with NaOH 0.2 M for 20 minutes, blocked again and incubated with the appropriate antibodies.

### Statistical Analysis

Results are presented as means ± S.E.M. of the number of experiments indicated performed in different preparations. The normality of the data was assessed using the Kolmogorov-Smirvov test. Statistical significance was assessed by one-way analysis of variance (ANOVA) followed by the Bonferroni's or Dunn's Multiple Comparison test, or by Student's *t*-test (always performed in pairs by comparing each OGD condition with the respective control), as indicated in the figure captions. These statistical analyses were performed using the software package GraphPad Prism 5.

## Results

### OGD induces delayed neuronal death in mature hippocampal neurons

**Figure 1:**
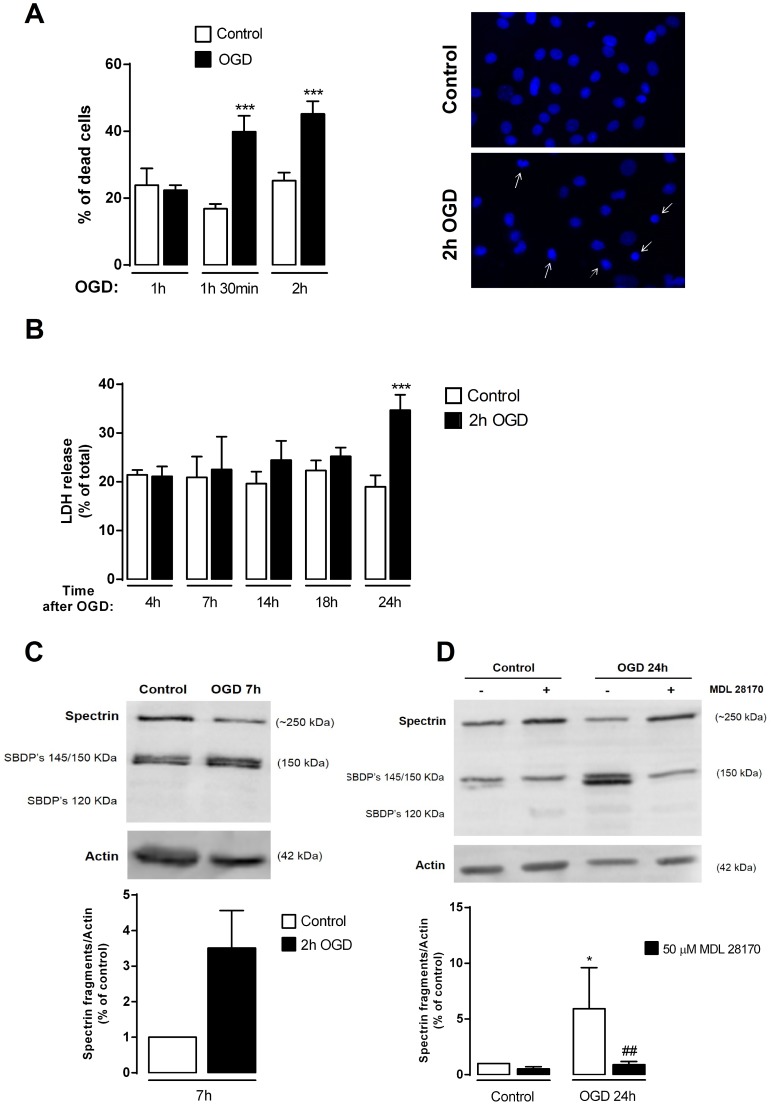
OGD induces delayed neuronal death of mature hippocampal neurons in culture. (**A**) OGD causes hippocampal neuronal cell death, as determined by analysis of nuclear morphology. After incubation under OGD conditions for 1 h (n = 3), 1 h30 (n = 12) and 2 h (n = 10), cells were returned to the 5% CO_2_ incubator for 24 h. Cell viability was then assessed by analysis of the nuclear morphology. Pyknotic nuclei (arrows) were counted as dead cells. The results were expressed as the percentage of dead cells relatively to the total cell number. The right panel depicts nuclear morphology of neurons subjected to control and 2 h OGD. (**B**) Time-course of OGD-induced neuronal death, as determined by LDH release. Cells were subjected to OGD for 2 h and the LDH release was assessed 0 h (n = 6), 4 h (n = 5), 7 h (n = 3), 14 h (n = 6), 18 h (n = 6) and 24 h (n = 13) after the stimulus. (**C–D**) OGD induces cleavage of spectrin and the formation of spectrin breakdown products (SBDPs). SBDPs protein levels were analyzed by Western blot 7 h (**C**) and 24 h (**D**) after 2 h of OGD. The calpain inhibitor MDL 28170 (50 µM) was added 30 min prior to stimulation and kept during the stimulation and post-stimulation period (24 h). Actin was used as loading control. Bars represent the mean ± SEM of five (**C**) or ten (**D**) independent experiments performed in distinct preparations. *p<0.05, ***p<0.001, as determined by the Student's *t*-test (**C**) and One-way ANOVA followed by Dunn's Multiple Comparison Test (**D**; *p<0.05 for the OGD condition compared to control, ##*p*<0.01 for the OGD+MDL28170 condition compared to OGD).

In order to characterize the neuronal injury induced by *in vitro* oxygen and glucose deprivation, primary hippocampal neuronal cultures were subjected to different periods of OGD, followed by 24 h incubation in culture conditioned medium, after which cell viability was evaluated by analysis of the nuclear morphology. Periods of OGD≥1 h 30 min resulted in a decrease in cell viability of nearly 20% (**Figure 1A**). Therefore, in all forthcoming experiments the cells were subjected to OGD for 2 h and further incubated in culture conditioned medium for the indicated periods of time.

To test whether OGD causes delayed neuronal death, we assessed cell viability at different periods after the insult, by quantification of the LDH release (**Figure 1B**). We observed that at post-incubation periods of up to 18 h after OGD there was no detectable change in cell viability, whereas at 24 h after the insult LDH release was increased, thus confirming that the OGD challenge induces delayed neuronal death, similarly to the ischemic insult *in vivo* [5], [18]–[20].

Previous studies have indicated that the intracellular effects of ischemia include the activation of calpains, a family of calcium-activated cysteine proteases which trigger substrate-specific proteolysis that may contribute to neuronal death [21], after both *in vivo* [22], [23] and *in vitro* ischemia [24]–[26]. Calpain activation (usually detected as the breakdown products of a preferred substrate, spectrin) can therefore serve as an indirect indicator of the induction of cell death. As expected, no significant OGD-induced calpain activation was observed 7 h (**Figure 1C**) after subjecting hippocampal neurons to the ischemic insult (a time point at which there is still no significant cell death, as assessed by quantification of LDH release, Figure 1B). However, at 24 h after the insult, significant activation of these proteases was observed, as indicated by the formation of the calpain-specific145 kDa spectrin cleavage product (**Figure 1D**). No 120 kDa cleavage products were detected, indicating that caspase-3 does not contribute to spectrin cleavage under the experimental conditions used [27]. We have used a calpain inhibitor (MDL 28170) to test whether the OGD-induced formation of the 145 kDa spectrin cleavage product was specifically mediated by calpain activation upon OGD (Figure 1D). We observed that in the presence of the calpain inhibitor there is a dramatic reduction in the formation of the OGD-induced spectrin cleavage product (145 kDa). Taken together, these results show that incubation under OGD (2 h) results in significant cell death in primary hippocampal neurons, 24 h after the insult. Therefore, this protocol can be used to investigate the ischemic response at earlier (7 h) or later (24 h) recovery periods after the insult.

### OGD-induced hippocampal neuronal death is prevented by glutamate receptor antagonists

**Figure 2:**
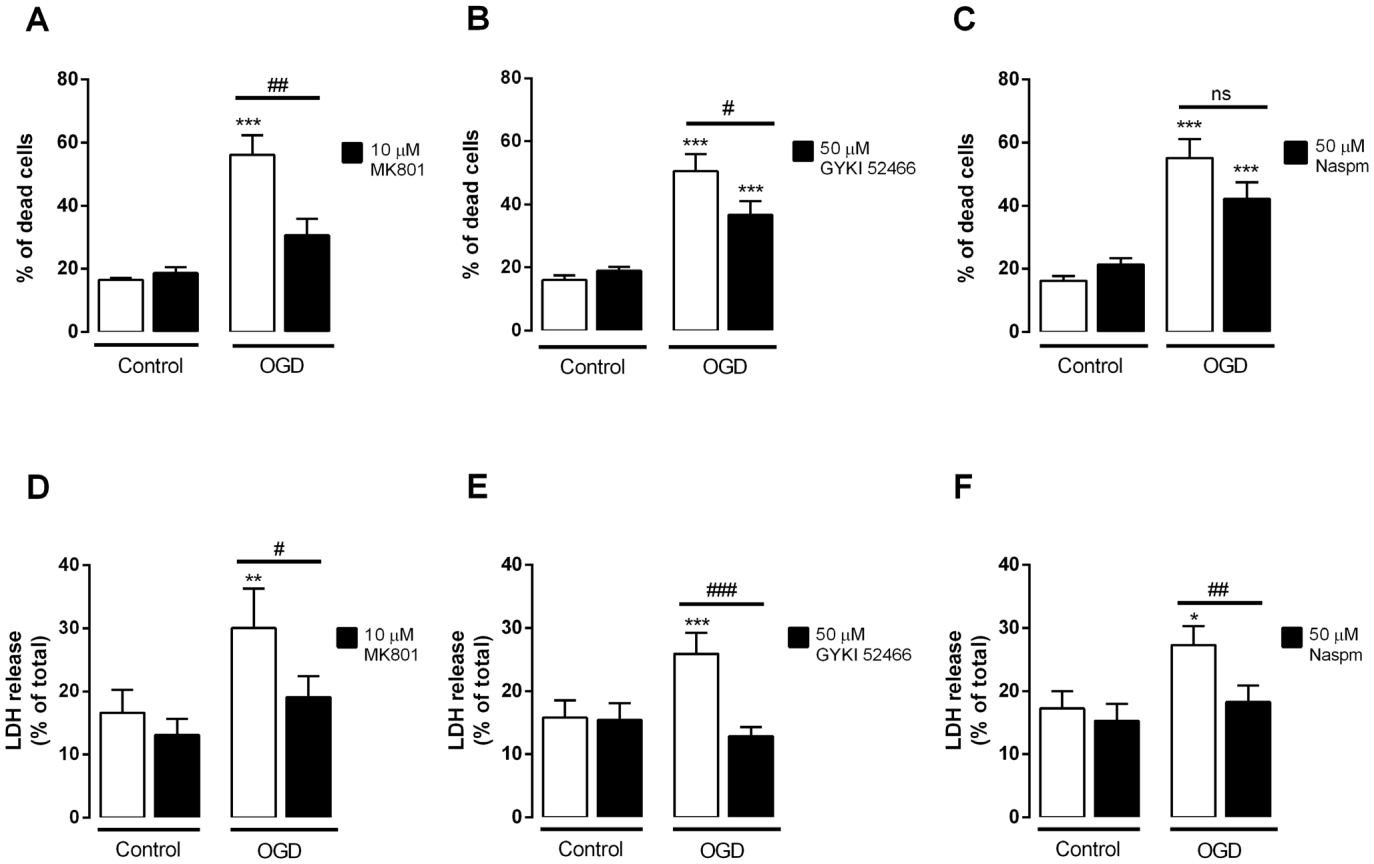
Inhibition of glutamate receptors protects hippocampal neurons against OGD-induced cell death. Mature hippocampal neurons were subjected to 2-stimulation periods. Cell viability was assessed 24 h after the stimulus by analysis of the nuclear morphology (**A–C**) and by determination of the LDH release (**D–F**). Bars represent the mean ± SEM of 4–9 independent experiments, performed in distinct preparations. Statistical analysis was performed using One-way ANOVA followed by Bonferroni's Multiple Comparison Test: *p<0.05, **p<0.01, ***p<0.001, relative to control; ^#^ *p*<0.05, ^##^ *p*<0.01, ^###^ *p*<0.001 relative to OGD condition. MK-801, selective NMDAR antagonist; GYKI 52466, selective AMPAR antagonist; Naspm, selective CP-AMPAR antagonist.

Glutamate toxicity due to overactivation of glutamate receptors, or *excitotoxicity*, has been previously related to cerebral ischemia and mediates an important component of global ischemia-induced neuronal damage, given that application of glutamate receptor antagonists attenuates synaptic transmission and neuronal death, conferring neuroprotection [28], [29]. To analyze the contribution of NMDA and AMPA ionotropic glutamate receptors to neuronal death elicited by OGD, hippocampal neuronal cultures were submitted to OGD in the absence or in the presence of MK801 (selective NMDAR antagonist), GYKI 52466 (selective AMPAR antagonist) or Naspm (selective Ca^2+^-permeable (CP) AMPAR antagonist). All antagonists were added prior to OGD and kept during the stimulation and post-stimulation periods. Cell viability was assessed 24 h after the stimulus by analysis of nuclear morphology (**Figure 2A–C**) and by quantification of LDH release (**Figure 2D–F**). Both assays showed that cell death induced by OGD was prevented by the NMDA and AMPA receptor antagonists. Also, the selective CP-AMPARs antagonist Naspm inhibited the increase in LDH release, indicating the partipation of this subtype of AMPARs in the neuronal death elicited by OGD. Taken together, these results confirm that both AMPA and NMDA receptors are involved in OGD-induced neuronal death and mediate the main excitotoxic component of this *in vitro* model of cerebral ischemia.

### OGD induces large-scale regulation of hippocampal gene expression

**Figure 3:**
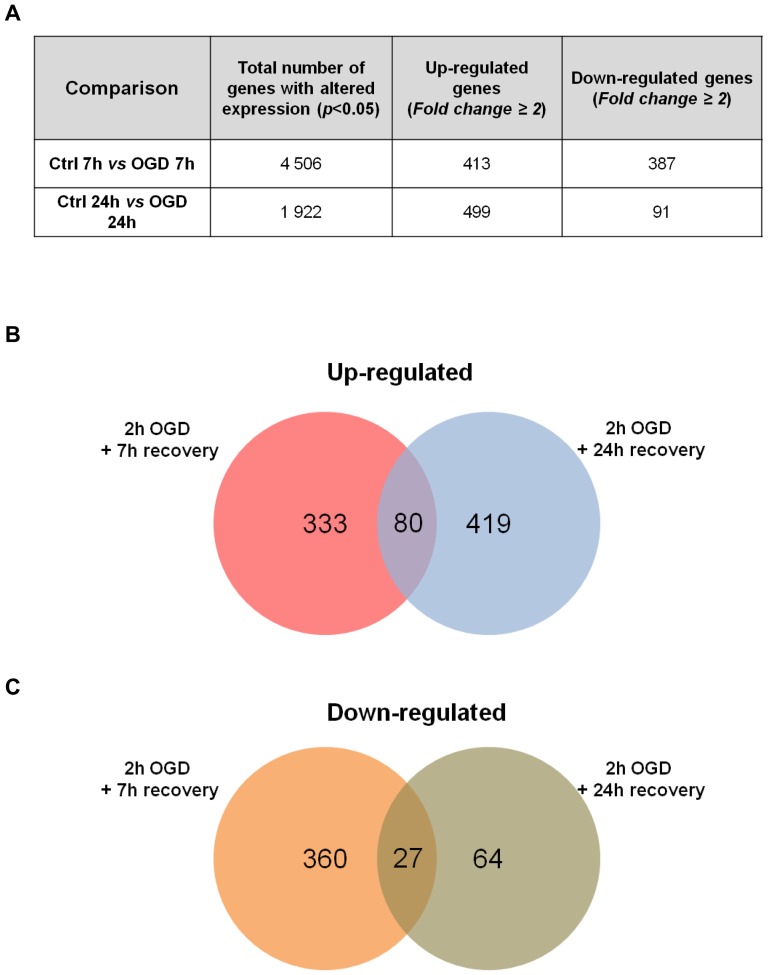
Summary of gene expression changes at 7 h and 24 h after OGD. (**A**) Student's *t*-test analysis was applied to the microarray data to identify all genes whose expression was significantly different between conditions (*p*<0.05). Up-regulated and down-regulated genes include those whose expression levels had a fold change ≥2.0. (**B and C**) Number of up-regulated (**B**) or down-regulated (**C**) transcripts at 7 h and 24 h after 2 h OGD. The intersection represents the number of transcripts whose transcription was changed in both recovery periods. The transcripts used in this analysis were considered differentially expressed after using two cut-off criteria: a *p*-value <0.05 and a fold change of 2.0. The VENNY informatic tool was used to compare the lists of transcripts to obtain the Venn Diagrams.

To investigate the molecular mechanisms involved in the neuronal response mediated by the OGD insult, we performed a whole-rat genome Agilent microarray analysis. Total RNA from rat hippocampal neuronal cultures submitted to control conditions or OGD were analyzed after 7 h and 24 h incubation in culture conditioned medium, in order to compare gene expression profiles at a time point prior to and after the onset of cell death. All experimental conditions were performed with three independent biological replicates. Student's *t*-test was used to determine the genes whose expression was significantly different between cells subjected to ischemic injury and the correspondent control. Only genes with *p*<0.05 and with an expression fold change of 2.0 relatively to the control condition were considered differentially expressed and selected for further analyses. From the approximately 44 000 probes present on each array, the expression levels of 4 506 transcripts were altered 7 h after OGD, whereas 1 922 transcripts were differently expressed at 24 h after OGD, when compared to their respective controls (*p*<0.05, Student's *t*-test). Of these, we observed that at 7 h and 24 h of incubation after OGD, the levels of a total of 413 and 499 transcripts were more than two fold altered in response to OGD, respectively (**Figure 3A**).

**Figure 3B–C** shows the number of genes that were exclusively altered at 7 h or 24 h after OGD, as well as the number of genes affected at both time points. Of all the transcripts up-regulated after OGD, the expression levels of 333 transcripts were increased specifically 7 h after injury, 419 transcripts were exclusively increased after 24 h after the insult and a total of 80 transcripts were found to be up-regulated after both time periods of incubation (**Figure 3B**). On the other hand, 360 transcripts were exclusively down-regulated at 7 h after OGD, whereas only 64 transcripts had reduced expression levels specifically at 24 h after OGD. A total number of 27 transcripts were down-regulated both at 7 h and 24 h after OGD (**Figure 3C**). These results indicate that whereas at 7 h after *in vitro* ischemia the changes in the transcriptome are related to both an up- and down-regulation of gene expression, at 24 h after the insult there is mainly up-regulation of gene expression.

### Transcriptional adaptations induced by OGD

**Figure 4:**
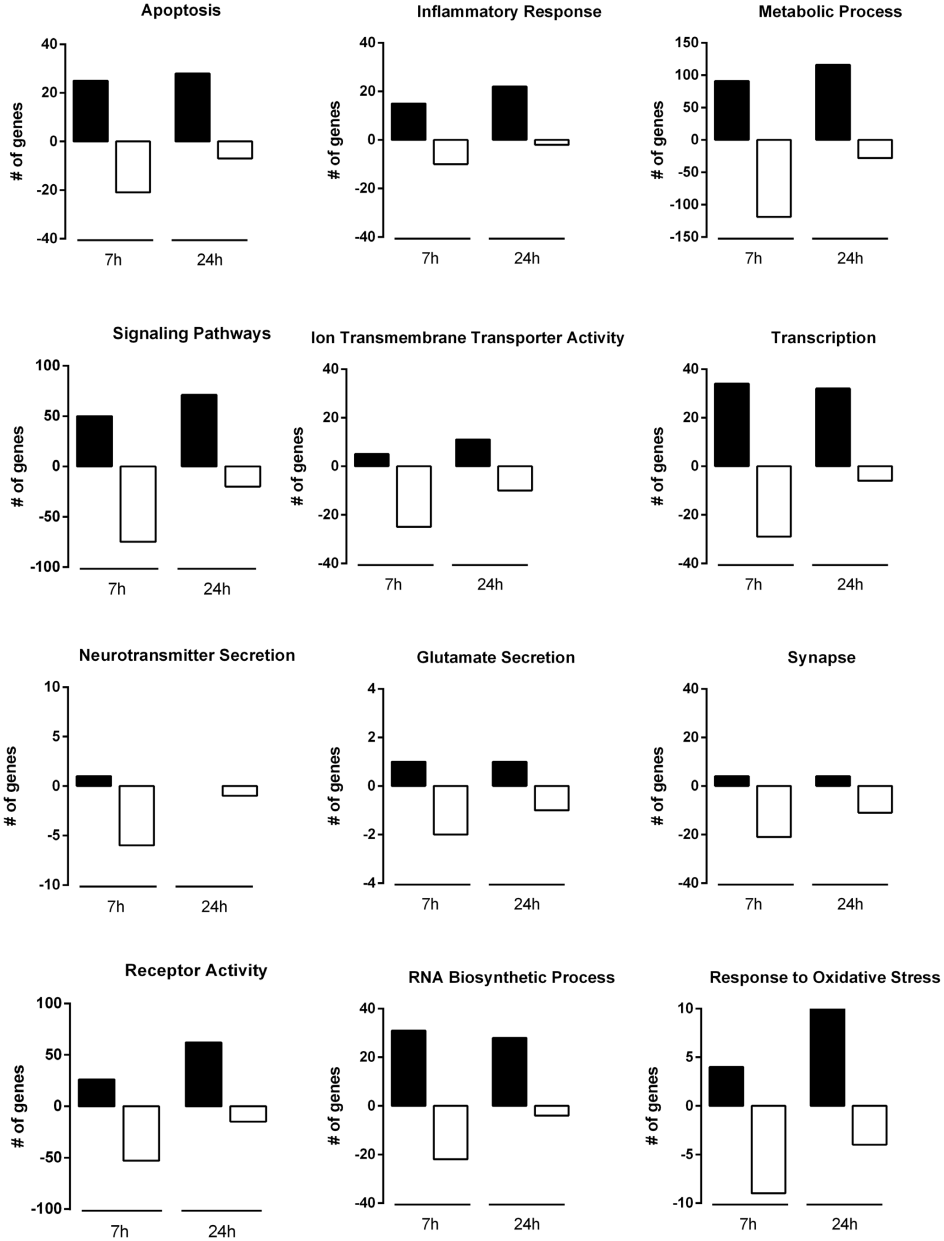
Time course analysis of OGD-induced differential gene expression within each functional gene category. The number of genes that were up-regulated (black bars) or down-regulated (white bars) at each period of recovery after the OGD insult is plotted for each functional gene category. Gene ontology analyses were performed using GoMiner and functional groups were selected manually.

To study the changes induced by the OGD insult in terms of functional gene groups, we next considered all the transcripts that displayed a significant expression increase or decrease at 7 h and 24 h of incubation after injury (or at both time points of recovery, where indicated) by gene ontology categories using the informatics tool GoMiner. The most up- and down-regulated genes at 7 h, 24 h or at both time points after OGD (*i.e.*, genes that are regulated as a constitutive response to *in vitro* ischemia) belong to a diverse set of categories and are indicated in **Tables S2–S4**. **Figure 4** shows the number of genes up- or down-regulated at 7 h and 24 h after OGD for different ontological classes. We found that genes related with metabolic processes, signaling pathways, receptor activity, transcription, RNA biosynthesis and apoptosis were the most altered after OGD. Genes included in other categories such as ion transmembrane transporter activity, inflammation and synapse were also highly regulated by the OGD challenge. Whereas the most up-regulated classes (and the number of genes with increased expression included in each one) are similar between 7 h and 24 h of recovery, the pattern of down-regulation is different. It is clear that at 7 h after OGD there are more genes whose expression is decreased than at 24 h, in all the classes considered (**Figure 4**).

**Figure 5:**
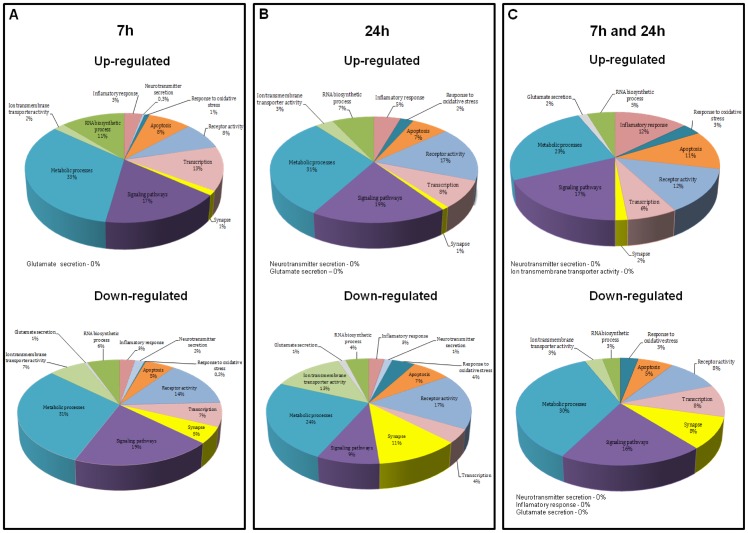
Ontology of genes differentially expressed at 7 h and 24 h of incubation after 2 h of OGD. Gene ontology analyses included genes that had a *p*-value <0.05 and a fold change of 2.0 and were performed using GoMiner. Classes were selected manually and the number of genes for each class divided by the sum of the total number of genes in the selected classes (586 genes at 7 h and 403 genes at 24 h). Note that some genes are included in more than one class. (**A**) Ontology of genes up-regulated (upper) and down-regulated (lower) relatively to the control at 7 h of incubation in culture conditioned medium after 2 h OGD. In this analysis, the total numbers of up-regulated and down-regulated genes were 232 and 354, respectively. (**B**) Ontology of genes up-regulated (upper) and down-regulated (lower) relatively to the control at 24 h of incubation in culture conditioned medium after 2 h OGD. In this analysis, the total numbers of up-regulated and down-regulated genes were 333 and 70, respectively. (**C**) Ontology of genes up-regulated (upper) and down-regulated (lower) at 7 h and 24 h of incubation in culture conditioned medium after 2 h OGD. In this analysis, the total numbers of up-regulated and down-regulated genes were 60 and 31, respectively.

We then selected the functional gene classes with potential relevance to the excitotoxic process, comprising a total of 586 and 403 genes that were altered at 7 h and 24 h after OGD, respectively, and analyzed changes in gene expression among genes belonging to those classes. In **Figure 5** we indicate the percentage of altered genes in ontology groups regulated exclusively at 7 h (**A**), 24 h (**B**) or regulated at both periods of recovery (**C**). The categories of metabolic processes and signaling pathways contained the largest percentage of regulated (up- or down-regulated) genes at both periods of incubation after OGD, both constitutively or time point-specific regulated genes. Additionally, differential effects were observed when comparing the expression profiles at 7 h and 24 h after injury. For example, at 24 h there was an increase in the percentage of up-regulated genes that code for proteins involved in the response to oxidative stress and receptor activity, whereas fewer genes were up-regulated in classes such as transcription and RNA synthesis, when compared to what is observed 7 h after OGD. Also, at 24 h more genes were down-regulated in classes such as response to oxidative stress and synapse, whereas genes coding for proteins related with transcription, RNA synthesis, metabolism and signaling pathways were less down-regulated. The complete lists of genes for every ontology group considered at 7 h, 24 h and both time points used in these analyses are shown in **Tables S5–S7.**

Taken together, these results demonstrate that the OGD model induces similar changes in functional groups of genes that have been shown to be differently regulated after *in vivo* ischemia [2]–[4], [6]–[9]. Moreover, although many genes were differentially expressed at both time points of recovery, most genes were exclusively altered at only one of the post-injury periods tested, suggesting that specific molecular pathways can be regulated at an early or late response to OGD.

### Genes coding for synaptic proteins are down-regulated after OGD

To validate the gene expression profiles obtained in the microarray assay, we performed qPCR analyses for 11 selected genes that were differentially regulated (up- and down-regulated) and belong to different functional categories (**Table S8**). Most of the genes tested with qPCR analysis showed changes in expression following OGD in the same direction as determined in the microarray experiment (**Figure S1**).

**Figure 6:**
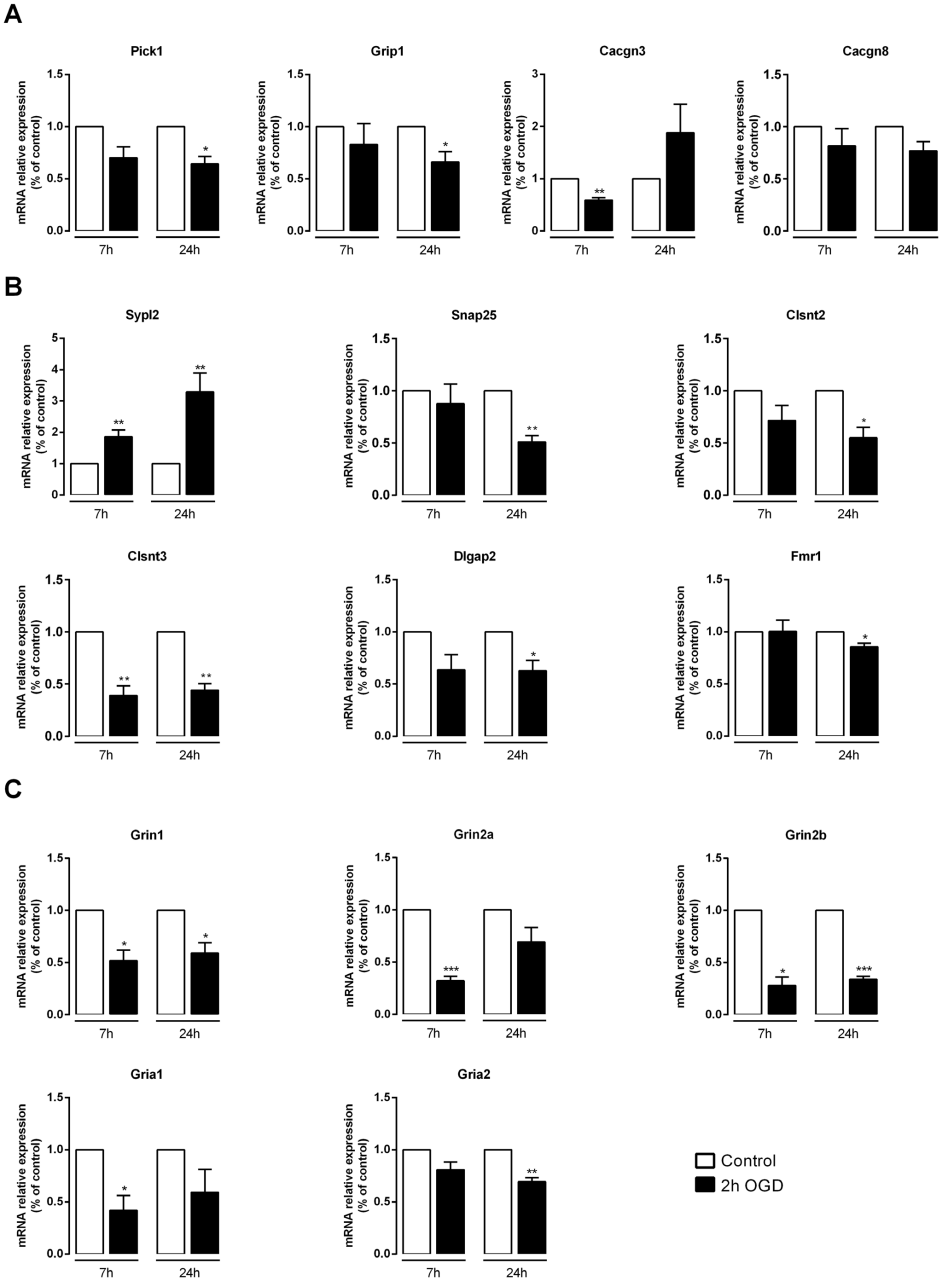
OGD induces changes in the mRNA levels of transcripts encoding synaptic proteins. Total RNA was extracted with TriZol 7 µg of total RNA and specific primers for each selected gene. Fold change in mRNA levels was normalized to Gapdh and Actb. Quantitative PCR analysis showed that genes encoding proteins associated with AMPAR trafficking (**A**) and pre- and post-synaptic compartments (**B**), as well as subunits of the AMPA and NMDA receptors (**C**) were mostly down-regulated (with the exception of Sypl2, which had increased expression levels) after OGD, at least at one of the time points analyzed after OGD. Bars represent the mean ± SEM of 5 independent experiments, performed in distinct preparations. *p<0.05, **p<0.01, ***p<0.001, as determined by the Student's *t*-test on log-transformed data.

According to the microarray data, genes coding for several synaptic proteins were differentially regulated after the OGD challenge (**Tables S5–S7, Synapse category**). We chose to validate the gene expression changes obtained in the microarray assay for 15 distinct synaptic protein genes (**Table 1**). These genes are of particular interest since they code for proteins involved in glutamatergic neurotransmission, whose imbalance contributes to ischemic injury. Thus, the expression levels of genes encoding proteins related with AMPAR trafficking (Pick1, Grip1, Cacgn3 and Cacgn8), pre- and post-synaptic compartments (Sypl2, Snap25, Clstn2, Clstn3, Dlgap2 and Fmr1) and subunits of the AMPA (Gria1 and Gria2) and NMDA (Grin1, Grin2a and Grin2b) receptors were analyzed by qPCR (**Figure 6**). Notably, most of these genes were down-regulated after the OGD insult, as indicated by the microarray experiment (**Table 1**) and confirmed by qPCR analyses (**Figure 6**). For instance, the results obtained for Cacgn3, Clsnt2 and Clstn3 correlated with the microarray data, since their expression levels were reduced at the same time points of incubation after OGD as in the microarray assay. Sypl2 was also in agreement with the microarray data, and it was the only synaptic protein gene confirmed to be up-regulated in response to the OGD insult; the up-regulation of Frm1 7 h after OGD indicated in the microarray data was not confirmed by qPCR. In some cases, qPCR analysis allowed the detection of a decrease in the mRNA levels of genes at earlier (Gria1 and Grin2a), later (Pick1, Snap25 and Dlgap2) or at both (Grip1, Grin1 and Grin2b) time points of incubation after OGD rather than just the one indicated in the microarray data. The down-regulation of Cacgn8, as indicated by the microarray data, was not detected by qPCR analysis, whereas Gria2 was found to have reduced mRNA levels at 24 h after the insult, which was undetected in the microarray experiment.

**Table 1:** Microarray data showing the effect of OGD in the mRNA levels of genes encoding synaptic proteins.

	Gene	Gene Name	Protein	Status	Fold Change
**AMPAR trafficking**	Pick1	Protein interacting with PRKCA 1	PICK1	Down-regulated at 7 h	0.48
	Grip1	Glutamate receptor interacting protein 1	GRIP1	Down-regulated at 7 h and 24 h	0.62 (7 h)/0.64 (24 h)
	Cacng3	Calcium channel, voltage-dependent, gamma subunit 3	TARP γ3	Down-regulated at 7 h	0.41
	Cacng8	Calcium channel, voltage-dependent, gamma subunit 8	TARP γ8	Down-regulated at 24 h	0.64
**Pre- and post-synaptic compartment**	Sypl2	Synaptophysin-like 2	SYPL2	Up-regulated at 7 h and 24 h	2.61 (7 h)/2.63 (24 h)
	Snap25	Synaptosomal-associated protein 25	SNAP-25	Down-regulated at 7 h	0.43
	Clstn2	Calsyntenin 2	Calsyntenin 2	Down-regulated at 24 h	0.44
	Clstn3	Calsyntenin 3	Calsyntenin 3	Down-regulated at 7 h and 24 h	0.44 (7 h)/0.38 (24 h)
	Dlgap2	Discs, large (Drosophila) homolog-associated protein 2	SAPAP2	Down-regulated at 7 h and 24 h	0.48 (7 h)/0.4 (24 h)
	Fmr1	Fragile X mental retardation 1	FMRP	Up-regulated at 7 h	2.34
**AMPAR subunits**	Gria1	Glutamate receptor, ionotropic, AMPA 1	GluA1	Down-regulated at 24 h	0.55
	Gria2	Glutamate receptor, ionotropic, AMPA 2	GluA2	No change	—
**NMDAR subunits**	Grin1	Glutamate receptor, ionotropic, *N*-methyl-D-aspartate 1	GluN1	Down-regulated at 24 h	0.39
	Grin2a	Glutamate receptor, ionotropic, *N*-methyl-D-aspartate 2A	GluN2A	Down-regulated at 24 h	0.44
	Grin2b	Glutamate receptor, ionotropic, *N*-methyl-D-aspartate 2B	GluN2B	Down-regulated at 24 h	0.44

In general, the qPCR analysis confirmed the OGD-induced changes in the expression levels of synaptic protein genes detected with the microarray data analysis. As such, our results show that OGD activates a transcriptional program leading to the repression of genes related with the synaptic function in hippocampal neurons, suggesting that changes at the synapse take place after the ischemic event.

### The expression levels of the silencing transcription factor REST increase after OGD

**Figure 7:**
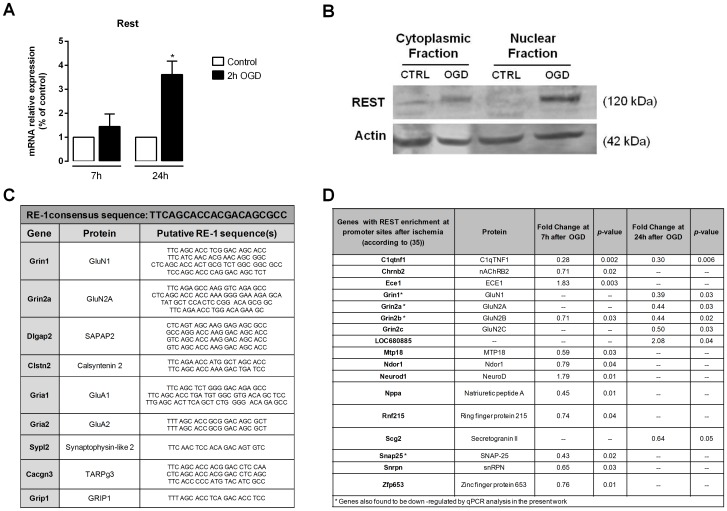
OGD increases REST expression in mature hippocampal neurons. (**A**) Quantitative PCR analysis showed the OGD insult induced a marked increase in Nrse (Rest) mRNA. Total RNA was extracted with TriZol 7 h and 24 h after the OGD insult. Quantitative PCR analysis was performed using cDNA prepared from 1 µg of total RNA and specific primers for each selected gene. Fold change in mRNA levels was normalized to Gapdh and Actb. Bars represent the mean ± SEM of three independent experiments, performed in distinct preparations. *Significantly different from control (*p<0.05, Student's *t*-test on log-transformed data). (**B**) Representative Western blot shows a marked increase in REST protein levels, both in the cytoplasmic and nuclear fractions of hippocampal neurons submitted to OGD followed by 24 h of incubation in culture conditioned medium (n = 3). Actin was used as loading control. (**C**) Putative RE-1 sequence(s)/REST-binding site(s) in synaptic genes (according to [35]) found to be down-regulated after OGD. (D) Genes with enrichment of REST after ischemia (according to [33]) found to be differently expressed after OGD.

The gene-silencing transcription factor REST (repressor element-1 silencing transcription factor) actively represses neuronal genes important for synaptic plasticity and remodeling, such as synaptic vesicle proteins, synaptic structural proteins and receptors, in progenitor and non-neuronal cells [30]–[32]. As neuronal differentiation takes place, REST is down-regulated, an essential process for the maintenance of the neuronal phenotype. Neuronal insults, such as transient global cerebral ischemia, activate REST in CA1 hippocampal neurons destined to die [20], [33], [34]. We therefore tested whether the OGD insult also triggers the induction of REST. In the microarray data the expression fold change for Rest was 1.63 at 7 h after OGD (*p* = 0.016). We also analyzed Rest mRNA expression in hippocampal cultures submitted to OGD by qPCR, and observed that even though at 7 h after the insult the mRNA levels of REST were not significantly different from the control, a significant increase was observed at 24 h (**Figure 7A**). Consistently, the increase in the mRNA levels of Rest translated in the induction of the REST protein levels at the same time-point after OGD (**Figure 7B**). These results corroborate previous observations suggesting that REST may be one of the transcriptional factors mediating the transcriptional response to ischemia. Indeed, 9 out of the 15 genes encoding synaptic proteins that were down-regulated after OGD contain putative REST-binding sites (**Figure 7C**, according to [35]) and may therefore be regulated by REST.

We next analyzed whether genes that have previously been described to have REST enrichment at their promoters after transient global brain ischemia in rats [33] were differently expressed after OGD (**Figure 7D**). Interestingly, analysis of the microarray data showed that several genes with enriched REST [33] have decreased expression levels at 7 h or 24 h of recovery after OGD; in the case of Snap25, Grin1, Grin2a and Grin2b, the down-regulation in their mRNAs was further confirmed by qPCR analysis in the present work (**Figure 6**). Gria2, the gene encoding GluA2, has been proven to be a REST target gene in the post-ischemic CA1 hippocampal region [20], [33], [34]. Although our microarray analysis did not detect significant differences in GluA2 expression, qPCR analysis showed down-regulation of GluA2 mRNA levels 24 h after OGD (**Figure 6C**). Collectively, these observations support previous evidence for the hypothesis that REST is activated under ischemic conditions [20], [33], [34]. As such, REST activation may be responsible for the repression in the transcription of some of the synaptic protein genes that we observed to be down-regulated under OGD.

### OGD down-regulates total and cell surface GluA1 protein levels

**Figure 8:**
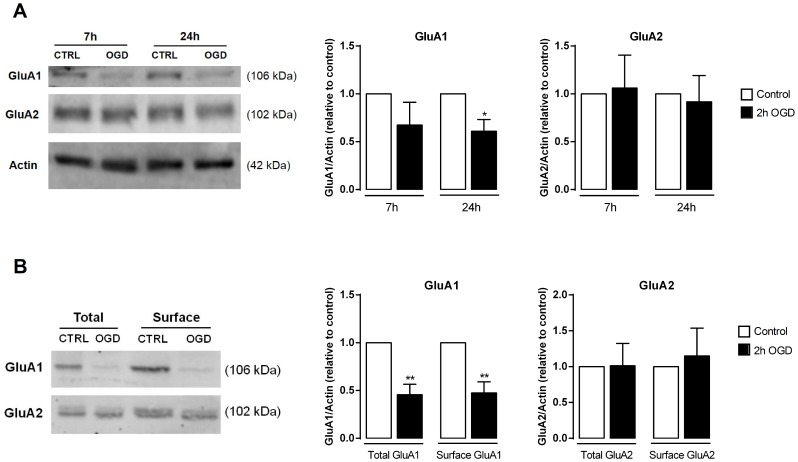
Ischemic insults affect the protein levels of the AMPAR subunits GluA1 and GluA2. (**A**) Total protein extracts were prepared 7 h and 24 h after the OGD insult and Western blot analysis were performed. Total GluA1 is decreased after 24 h, whereas GluA2 levels remain unaltered following both periods of recovery. The left panel shows a representative Western blot for total GluA1 and GluA2 present in cell lysates after OGD. The right panel represents the quantification of the Western blots. Bars represent the mean ± SEM of five independent experiments, performed in ditinct preparations. (**B**) GluA1 and GluA2 surface levels were analyzed after biotinylation of cultured hippocampal neurons, 24 h after the OGD insult. At this time point after OGD GluA1 is removed from the surface, whereas GluA2 surface levels remain unaltered. The left panel shows a representative Western blot for surface GluA1 and GluA2 after the insult. Total actin (from the total extract) was used as loading control. Bars represent the mean ± SEM of 3–4 independent experiments, performed in distinct preparations. *p<0.05, as determined by the Student's *t*-test.

Previous studies indicate that both transient global cerebral ischemia and *in vitro* ischemic insults can down-regulate the AMPAR subunit GluA2, both at the mRNA and protein levels, in hippocampal neurons [11], [12], [20], [33], [36]–[39], thus inducing a switch from GluA2-containing/Ca^2+^-impermeable AMPARs to GluA2-lacking/Ca^2+^-permeable AMPARs. However, according to our qPCR data, the mRNA levels of both GluA1 (Gria1) and GluA2 (Gria2) subunits were decreased after the OGD insult (**Figure 6C**). We therefore tested whether total protein levels of GluA1 and GluA2 varied in the same direction as their respective mRNAs, at 7 h and 24 h after OGD. Despite the reduction in the mRNA levels of GluA1 observed after 7 h after injury, the decrease in the protein levels was detected only 24 h after OGD. Curiously, GluA2 protein levels remained unaltered after OGD, in spite of the reduction in its mRNA levels detected by qPCR at 24 h after the insult (**Figure 8A**).

We then biotinylated cell surface proteins in hippocampal neuronal cultures 24 h after incubation under control or OGD conditions, and purified biotinylated proteins by affinity chromatography to analyze the cell surface content of both subunits of AMPAR (**Figure 8B**). We observed that, consistent with the results obtained for the total protein levels, surface GluA1 was reduced 24 h after OGD, whereas surface GluA2 expression was unaffected. These results show that AMPARs present at the cell surface in a mixed population of hippocampal neurons subjected to OGD have a decreased content on GluA1 when evaluated 24 h after the insult, resulting from a delayed reduction in the mRNA levels of GluA1 in these experimental conditions. The reduction in AMPAR GluA1 content that we detected may represent a neuroprotective mechanism occurring in specific neuronal subtypes in the preparation.

### OGD down-regulates GluN2 subunits and increases the expression levels of GluN3A

**Figure 9:**
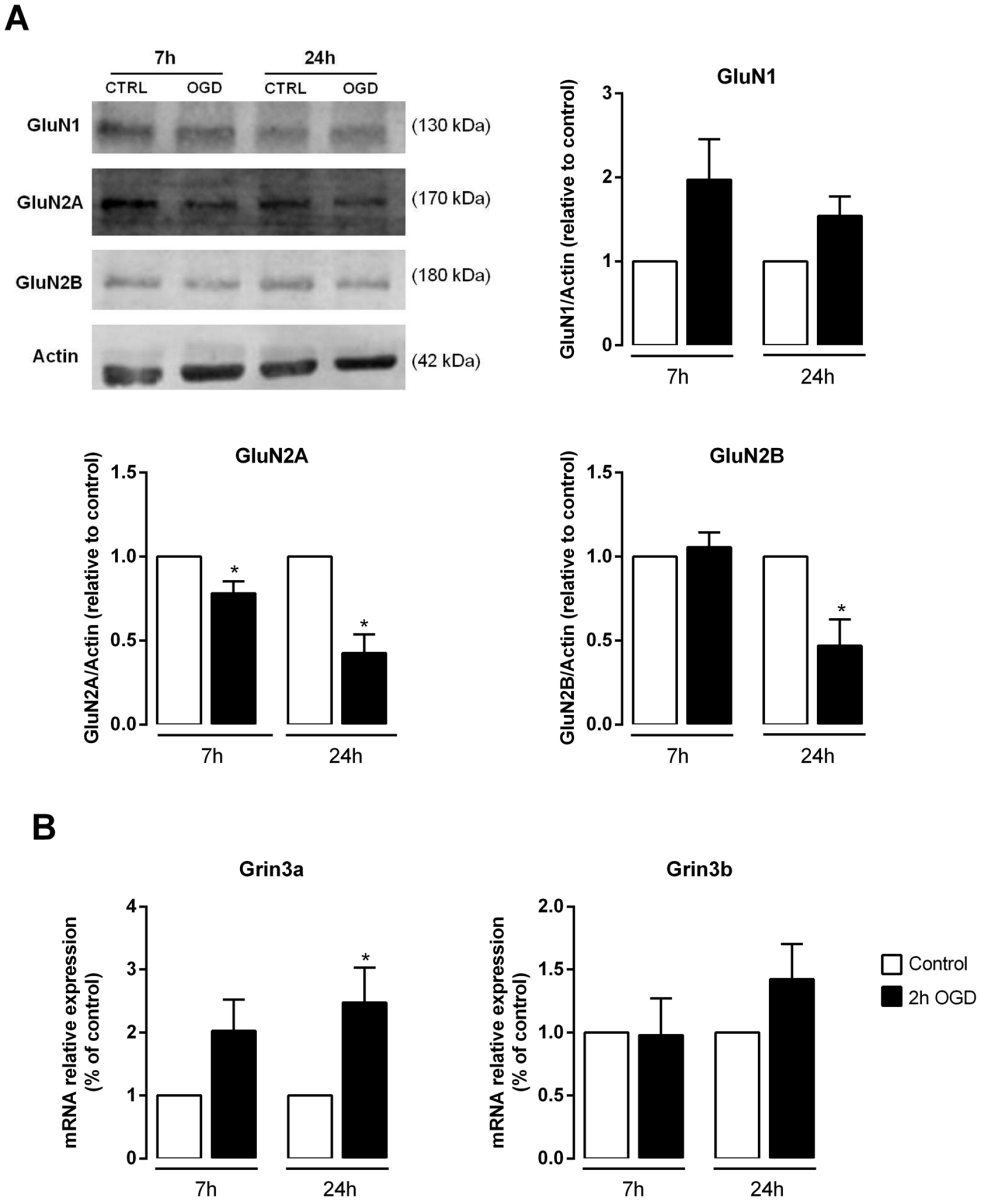
OGD affects the expression levels of NMDAR subunits. (**A**) Total protein extracts were collected 7 h and 24 h after OGD and Western blot analysis was performed. Whereas total GluN1 levels tend to increase after the insult, a decrease in total GluN2A was observed after both periods of recovery while GluN2B decreased at 24 h after the OGD insult. The left panel shows a representative western blot for GluN1, GluN2A and GluN2B levels after OGD. The right panel represents the quantification of the Western blots. Actin was used as loading control. Bars represent the mean ± SEM of 5–9 independent experiments, performed in distinct preparations. (**B**) OGD induces a marked increase in the mRNA levels of GluN3A but not in GluN3B. Bars represent the mean ± SEM of 3–5 independent experiments, performed in distinct preparations. *p<0.05, as determined by the Student's *t*-test.

The NMDA subtype of ionotropic glutamate receptors has been extensively studied in ischemic conditions due to their overactivation by glutamate and subsequent contribution to the activation of pathologic molecular pathways [29], [40]. In the *in vitro* ischemia model used in the present work, cell death is also dependent on the activation of NMDA receptors (**Figure 2A, D**). According to our qPCR data, the mRNA levels of GluN1 (Grin1) and GluN2B (Grin2b) were down-regulated both at 7 h and 24 h of incubation after OGD, whereas GluN2A (Grin2a) was down-regulated only at 7 h after the ischemic injury (**Figure 6C**). We then analyzed the total protein levels of these subunits by Western blot (**Figure 9A**). At 24 h after OGD, protein levels for both GluN2A and GluN2B were decreased, whereas GluN1 was not significantly changed, despite a clear tendency for increased expression. Together, these results indicate that OGD leads to the down-regulation of the most abundant GluN2 subunits in the hippocampus, thereby influencing the subunit composition of NMDARs.

Given that the total protein levels of GluN1 failed to accompany the decrease on protein levels found for both GluN2A and GluN2B subunits, we hypothesized that OGD may influence the expression levels of the GluN3 subunit, which can also promote GluN1 trafficking to the cell surface [41], [42]. In fact, analysis of the mRNA levels of the GluN3 subunit revealed that GluN3A displayed a remarkable increase in the mRNA expression 24 h after OGD, whereas GluN3B mRNA levels remained unaltered at both periods of recovery (**Figure 9B**). This suggests that OGD specifically induces the expression of GluN3A, but not GluN3B, thus promoting the presence of GluN1-GluN3A receptors in neurons submitted to ischemic insults. This result is in accordance with recent observations indicating that GluN3A can be up-regulated in response to ischemic insults and might therefore exert a neuroprotective effect against neuronal damage [43].

## Discussion

Transient global cerebral ischemia induces a profound change in the transcriptome of brain cells, which is partially associated with the induction or repression of specific genes that influence the outcome of the ischemic insult. However, the mechanisms responsible for the selective vulnerability of hippocampal neurons to global ischemia remain to be clarified. As such, in this study we have subjected mature primary cultures of hippocampal neurons to OGD, a well-established *in vitro* model for global ischemia, in order to identify molecular changes that may be involved in the response of hippocampal neurons to an ischemic insult. The OGD challenge induced delayed neuronal death in hippocampal cultures and activated an excitotoxic component mediated by NMDA and AMPA receptors as observed in previous studies [11], [12], [25], [44]. OGD also induced the activation of calpains, which corroborates previous studies showing that these proteases can play an important role in the enhancement of cell death induced by *in vitro* ischemic insults [24]–[26].

Although the OGD model has been used to infer about changes in gene expression associated with pre- and post-conditioning-derived neuroprotection [45], [46], to the best of our knowledge no similar large scale study was developed so far using this insult as a tool to study ischemia-induced changes in the transcriptome of hippocampal neurons at different periods of recovery. Our analysis has allowed the detection of a large number of altered genes related with the specific response of neurons to the ischemic insult. The validity of our approach was confirmed by performing qPCR analysis of many selected genes belonging to different functional groups, including the synapse, which was in good agreement with the microarray data.

At an early time point (7 h) after OGD there are more differentially regulated transcripts than at 24 h, which corroborates the notion that the response to ischemia is a dynamic and coordinated process that starts soon after the insult, but is extended until later time points. In fact, this is supported by the observation that genes related with functions such as transcription and RNA biosynthesis are highly regulated at both periods of recovery. We also noted that the response at 7 h after OGD is more associated with a general repression of genes than at 24 h, at which point there is a general induction of gene expression. This observation further supports the idea that the response to ischemia at a delayed time point is still part of an active process that rather involves the induction than the repression of genes, and excludes the possibility that an unspecific down-regulation of genes could be associated with an increased rate of cell death.

Consistent with the results obtained using gene ontology analysis after *in vivo* ischemia [2]–[4], [6]–[9], we found that genes related with metabolic processes, signaling pathways, receptor activity, transcription, inflammation, neurotransmitter/glutamate secretion, RNA biosynthesis and apoptosis were differentially regulated after OGD. These categories were chosen due to their relevance in identifying the molecular pathways involved in the selective vulnerability of hippocampal neurons to ischemic insults. According to our microarray and qPCR data, most of the synaptic protein genes considered in this work showed down-regulation during the recovery periods after the OGD insult. As far as we know, this is the first study to detect changes in the expression levels of an extensive group of genes coding for synaptic protein genes upon ischemia. Down-regulation of some synaptic protein genes (such as Snap 25, Gria 1,2, and Grin1, 2a) has been reported previously in studies regarding changes in gene expression after focal [6] and global [2] ischemia, but the other synaptic protein genes that we identified had not been previously reported to be altered after ischemic insults.

Although cerebral ischemia can induce a reduction of spines and synapses as neurons degenerate [13], [47], the down-regulation of specific synaptic protein genes suggests the initiation of an adaptive response to hyperexcitability in post-ischemic neurons. The group of genes involved in synaptic function that we identified code for proteins that can be involved in many regulatory processes, *i.e.* the cycling of synaptic vesicles and neurotransmitter release at nerve terminals (Snap25), trafficking and stabilization of glutamate receptors at the cell surface (Pick1, Grip1, Cacgn3 and 8) and propagation of neuronal signal impulses upon cell stimulation (AMPAR and NMDAR subunits).

SNAP-25 (synaptosomal-associated protein, 25 kDa) is involved in synaptic vesicle membrane docking and fusion mediated by SNAREs, therefore contributing to the regulation of neurotransmitter release in presynaptic terminals. Moreover, SNAP-25 has been shown to have a critical role in PKC-induced, SNARE-dependent insertion of NMDAR at synaptic sites, a mechanism relevant to synaptic plasticity [48]. A decrease in the mRNA levels of SNAP-25 at a later time point after the ischemic insult, as our data shows, might consist of a protective strategy of neurons to reduce neuronal hyperexcitability by reducing the release of glutamate as well as the insertion of NMDAR at the plasma membrane. PICK1 (protein interacting with C kinase) and GRIP1 (glutamate receptor interacting protein) are both involved in the trafficking of AMPAR [49]. Under physiologic conditions PICK1 binds to AMPAR subunits GluA2, an interaction known to be required for AMPAR internalization [50]. In hippocampal neurons subjected to OGD, rapid PICK1-mediated GluA2 internalization during the OGD insult was found to contribute to cell death mediated by GluA2-lacking Ca^2+^-permeable AMPARs [12]. A delayed decrease in the levels of PICK1 following the reduction in its mRNA levels would probably account for the maintenance of the GluA2 subunit at the cell surface of post-ischemic neurons, as observed in our work. Interestingly, the gene coding for TARPγ3 was also shown to be down-regulated after OGD, suggesting another mechanism through which trafficking of AMPARs to the cell surface is changed after ischemia. Moreover, it has been shown that genetic deletion of TARPγ8 selectively abolishes sustained depolarizations in hippocampus mediated by kainate activation of AMPA receptors [51]. Therefore, it would be interesting to confirm whether the protein levels of TARPγ3 or TARPγ8 follow the same pattern of their respective mRNAs and, if so, their implication for the transport of AMPARs in ischemia. FMRP (Fragile X mental retardation protein) is a dendritic modulator of mRNA transport and translation repression, and knock-out mice present reduced levels of many postsynaptic proteins [52]. SAPAP2 (Synapse-associated protein 90/postsynaptic density-95-associated proteins, encoded by Dlgap2) is a member of the SAPAP family of postsynaptic proteins that can interact with various synaptic components, including the NMDARs [53]–[55]. Since both the genes encoding for FMR1 and SAPAP2 were down-regulated after OGD, a decrease in the respective proteins could hypothetically play a role in the re-organization of functional multiprotein units at post-ischemic synapses. Additionally, calsyntenin-3 (Clsnt3), which is dramatically reduced after OGD, has been recently attributed a function in promoting synapse development, with Clstn3^−/−^ mice showing compromised inhibitory and excitatory neurotransmission [56]. Overall, our results suggest that neurons can activate a program to decrease the expression of genes coding for proteins involved in the machinery of synaptic transmission, which might contribute to reduce glutamate-mediated signaling and excitotoxicity.

We also obtained evidence that the transcription factor REST is induced upon an OGD insult to cultured hippocampal neurons, in agreement with previous studies showing an increase of REST mRNA in the CA1 region of the hippocampus [20], [33]. Noteworthy, knockdown of REST protected CA1 neurons from OGD-induced death, thus suggesting that expression of REST is causally related with neuronal death [20], [34], [57]. One of the most studied targets of REST repressive activity is the AMPAR subunit GluA2, to whose promoter REST can bind, thus suppressing GluA2 expression [20], [32]–[34]. The increase in REST levels promoted by ischemic insults has also been shown to correlate with the down-regulation of the Na^+^-Ca^2+^ exchanger 1 (NCX1) [57]. However, there is also evidence pointing to a neuroprotective role of REST in some circumstances. Formisano and co-workers showed the REST-mediated repression of the expression of the opioid receptor 1 in interneurons, under ischemia conditions, was neuroprotective [59]. Recent evidence suggests that REST can have a crucial role in mediating neuroprotection in the ageing brain [58]. Indeed, this study shows that whereas in healthy ageing neurons REST is significantly up-regulated and represses many genes associated with cell death pathways, nuclear REST expression is substantially reduced in patients with age-related neurodegenerative diseases such as Alzheimer's disease. In these neurons, reduced REST binding leads to increased expression of many genes related with Alzheimer's disease pathology. The repression of cell death-associated genes therefore suggests that REST might be neuroprotective [58].

Other genes have been identified with REST-occupied target sequences (RE1 sites) after a genome-wide approach using serial analysis of chromatin occupancy in HEK cells [35], among which are some of the synaptic protein-encoding genes analyzed in the present work (**Figure 7C**). Interestingly, we found that several genes previously described to have REST enrichment at their promoters after in vivo ischemia [33] have reduced mRNA levels after the OGD insult (Figure 7D), thus providing a new list of REST targets with a potential role in ischemia, and further supporting the role of REST as a repressive transcription factor under ischemic conditions. Indeed, our observations are supported by other studies showing REST enrichment at the promoters of Grin1, Grin2a, Grin2b and Snap25 [33], as well as by two recent reports confirming the regulation of Grin2a and Snap25 by REST [58], and identifying Gria1, Gria2 and Grin1 as REST targets [60]. Overall, these studies confirm the binding of REST to the promoter of some of the synaptic protein genes that we found to be down-regulated after OGD and which contain a putative REST binding site. Of note, our study proposes novel REST targets, such as the SAPAP2, Calsyntenin 2, Synaptophysin-like 2, TARPγ3 and GRIP1 genes. Since several of the synaptic protein genes we found to be down-regulated have putative REST-binding sites, it will be of interest to confirm whether REST can indeed bind to the promoter of these genes under ischemic conditions.

Interestingly, ischemia-induced changes in the protein levels of synaptic components suggest that important alterations occur at the synapses of post-ischemic neurons. In particular, our results support the hypothesis that transcription-dependent mechanisms take place in insulted neurons that promote the reduction of neuronal activity through down-regulation of the expression of central synaptic players. In particular, given that NMDARs and AMPARs have been long considered important targets for therapeutical intervention, information concerning their post-ischemic expression levels is of the upmost interest.

Our results indicate that at 24 h after a 2 h OGD insult, AMPARs present at the cell surface of cultured hippocampal neurons have a decreased content of GluA1 subunits, whereas GluA2 protein levels were unchanged. Previous studies using cultured hippocampal neurons and the OGD stimulus showed early effects at the level of AMPAR traffic. Brief OGD caused the internalization of synaptic GluA2-containing AMPAR in hippocampal neurons [11], [12]. Other studies indicated that global ischemia triggers the reduction of GluA2 expression in the hippocampus CA1 region neurons, both at the mRNA [36], [39] and protein levels [37], [38], resulting in increased levels of Ca^2+^-permeable AMPA receptors. In the present study, the antagonist for Ca^2+^-permeable AMPAR, Naspm, protected hippocampal neurons from OGD-induced cell death (**Figure 2F**), supporting, along with other studies (e.g. [37]), a function for Ca^2+^-permeable AMPAR in ischemia-induced cell death. However, in cultured hippocampal neurons we failed to detect changes in GluA2 expression and traffic reported by others, presumably because these changes are cell-type specific and become diluted in a mixed population of hippocampal neurons. Also, the intensity of the ischemic insult might influence transcriptional and post-transcriptional regulatory mechanisms which may explain different results obtained in distinct works. Nevertheless, our study has shown for the first time a dramatic decrease in the total mRNA and protein levels of GluA1 24 h after a 2 h OGD insult. The decreased levels of GluA1-containing AMPARs likely result in a depression of synaptic transmission, with consequences similar to the increased internalization of AMPARs found in CA3 pyramidal neurons following a 15 min OGD protocol [61]. Both the endocytosis of AMPAR [61] and the delayed decrease on GluA1 expression after OGD that we describe here are potentially neuroprotective mechanisms.

Furthermore, we found that the subunit composition of NMDARs is altered after the OGD insult, which is in agreement with previous studies showing a down-regulation of genes encoding for subunits of the NMDARs after *in vivo* [6], [62]–[64] and *in vitro* [13], [65] ischemia. In our experimental system, OGD decreased the total mRNA and protein levels of the GluN2 subunits without affecting the protein levels of the GluN1 subunit (**Figure 9**). Additionally, we investigated the role of GluN3 subunit (GluN3A and GluN3B) expression in hippocampal neurons submitted to OGD since the GluN3 subunits are now recognized players in the modulation of NMDAR activity [66]. Interestingly, GluN3A expression is induced by OGD (**Figure 9B**), corroborating the results obtained in a recent study showing that GluN3A protein levels increase in neurons submitted to OGD and focal ischemia [43], but in disagreement with an earlier report showing that focal ischemia down-regulates GluN3A at the mRNA and protein levels 24 h after the insult [67]. Although GluN3 subunits can be found in many brain tissues including the hippocampus [68], [69], the expression of these subunits is temporally restricted. Whereas GluN3A is predominantly expressed during early development and diminishes to lower levels in adulthood, GluN3B levels are low during early development and gradually increase into adulthood [70]. Compared with NMDARs comprised of GluN1/GluN2 subunits, GluN3-containing NMDARs exhibit several different properties, including lower amplitude currents, lower calcium permeability and reduced Mg^2+^ sensitivity [70]–[76]. As such, GluN3 subunits are considered to have a dominant-negative effect upon NMDAR activity, which can be an advantage under pathologic conditions. Indeed, the neuroprotective role of GluN3A against excitotoxicity and ischemia-mediated injury has been already shown [43], [77]. Cultured neurons from GluN3A knock-out mice display greater vulnerability to toxic NMDA application, whereas neurons expressing transgenic GluN3A are more resistant to NMDA-mediated neurotoxicity and focal ischemia than wild-type neurons [77]. Although some evidence suggests that the protective role of GluN3A is associated with a decrease in Ca^2+^ permeability and in the production of ROS mediated by NMDARs [43], further studies are required to confirm the up-regulation of GluN1/GluN3A receptors upon ischemic insults and to understand the role mediated by these receptors in post-ischemic neurons. Importantly, as no significant changes were observed for the GluN3B subunit, neither at the mRNA (the present study) or protein [43] levels after ischemic insults, it is probable that this subunit is not involved in the pathological response mediated by ischemia, at least in hippocampal neurons.

## Conclusions

The present study shows that the OGD model not only mimics cell death events induced by cerebral ischemia, but can also serve, when combined with the microarray technology, as a useful tool to gain insight into particular cellular and molecular mechanisms evoked by global ischemia in hippocampal neurons. Notably, our results suggest that OGD induces the activation of a transcriptional program that represses the expression of many synaptic protein genes that encode for proteins mainly associated with glutamatergic neurotransmission. In particular, we have shown that OGD alters the expression of AMPA and NMDA receptor subunits that modulate the ion channel activity of the receptors, namely Ca^2+^-permeability. Given that NMDA and AMPA receptors have long been considered important targets for therapeutical intervention, information concerning the post-ischemic expression levels of these receptor subunits is crucial to guarantee efficacy of potential neuroprotective strategies concerning the activation and further downstream signaling mediated by these receptors.

## Supporting Information

Figure S1 **Effect of OGD followed by 7 h or 24 h of incubation in culture conditioned medium in the mRNA levels of 11 selected genes, compared to the respective control.** Total RNA was extracted with TriZol at 7 h and 24 h after OGD. Quantitative PCR analysis was performed using cDNA prepared from 1 µg of total RNA and specific primers for each selected gene. Fold change in mRNA levels was normalized to Gapdh and Actb. Bars represent the mean ± SEM of 5 independent experiments, performed in different preparations. *p<0.05, **p<0.01, as determined using the Student's *t*-test on log-transformed data.
(TIF)

Table S1 List of primer sequences used to analyze gene expression by qPCR.
(DOCX)

Table S2 Most strongly up-regulated (**A**) and down-regulated (**B**) genes at 7 h after OGD (fold change ≥2).
(DOCX)

Table S3 Most strongly up-regulated (**A**) and down-regulated (**B**) genes at 24 h after OGD (fold change ≥2).
(DOCX)

Table S4 Most strongly up-regulated (**A**) and down-regulated (**B**) genes at both recovery periods (7 h and 24 h) after OGD (fold change ≥2).
(DOCX)

Table S5 List of genes up-regulated and down-regulated at 7 h after OGD for different ontological classes. Gene ontology analyses included genes that had a *p*-value <0.05 and a fold change of 2.0 and were performed using GoMiner. Classes were selected manually. Note that some genes are included in more than one class.
(DOCX)

Table S6 List of genes up-regulated and down-regulated at 24 h after OGD for different ontological classes. Gene ontology analyses included genes that had a *p*-value <0.05 and a fold change of 2.0 and were performed using GoMiner. Classes were selected manually. Note that some genes are included in more than one class.
(DOCX)

Table S7 List of genes up-regulated and down-regulated at both 7 h and 24 h after OGD for different ontological classes. Gene ontology analyses included genes that had a *p*-value <0.05 and a fold change of 2.0 and were performed using GoMiner. Classes were selected manually. Note that some genes are included in more than one class.
(DOCX)

Table S8 Microarray data of the genes selected for validation through qPCR analyses.
(DOCX)
