# Supplementary figures and images for: In Vitro Ischemia Triggers a Transcriptional Response to Down-Regulate Synaptic Proteins in Hippocampal Neurons
Source: PLoS One. 2014 Jun 24;9(6):e99958. doi: 10.1371/journal.pone.0099958 (PMC4069008; doi:10.1371/journal.pone.0099958)

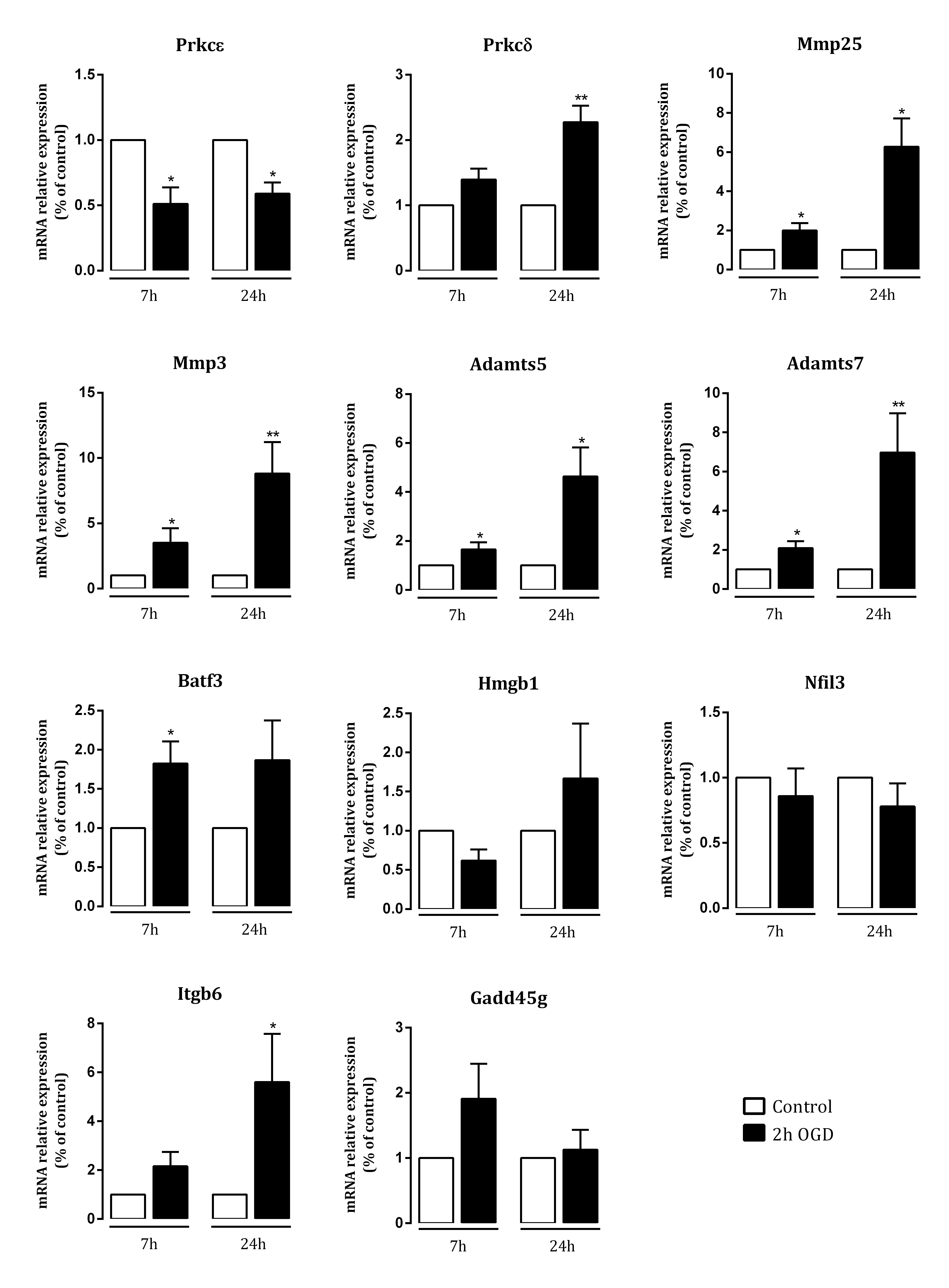

Supplement: Figure S1 — Effect of OGD followed by 7 h or 24 h of incubation in culture conditioned medium in the mRNA levels of 11 selected genes, compared to the respective control. Total RNA was extracted with TriZol at 7 h and 24 h after OGD. Quantitative PCR analysis was performed using cDNA prepared from 1 µg of total RNA and specific primers for each selected gene. Fold change in mRNA levels was normalized to Gapdh and Actb. Bars represent the mean ± SEM of 5 independent experiments, performed in different preparations. *p<0.05, **p<0.01, as determined using the Student's t-test on log-transformed data. (TIF) [file pone.0099958.s001.tif]
