# Supplementary material for: In Vitro Ischemia Triggers a Transcriptional Response to Down-Regulate Synaptic Proteins in Hippocampal Neurons
Source: PLoS One. 2014 Jun 24;9(6):e99958. doi: 10.1371/journal.pone.0099958 (PMC4069008; doi:10.1371/journal.pone.0099958)
Supplement: Table S1 — List of primer sequences used to analyze gene expression by qPCR. (DOCX) [file pone.0099958.s002.docx]

**C**

| **Gene** | **Primers** | **Anneling Temperature** |
| --- | --- | --- |
| **Actb** | Forward: CGTCACCTACTCTAACCG  Reverse: CTTGTGCTATCTGCTCATC | 52.8 |
| **Gapdh** | Forward: AACCTGCCAAGTATGATG  Reverse: GGAGTTGCTGTTGAAGTC | 53.4 |
| **Batf3** | Forward: ATGATGACAGGAAGGTTC  Reverse: CTCCAGACTCTCATGTTC | 58 |
| **Hmgb1** | Forward: AATACGGATTGCTCAGGAA  Reverse: GGACAACTGGTACTAATATGC | 55 |
| **Nfil3** | Forward: CTCTCCCTGAAATTAAAGTTTG  Reverse: GCTGTGGAATTACTGAGTT | 55 |
| **Itgb6** | Forward: ATAAGCCTCTCAGCGTAG  Reverse: CTCAACTTAAGAACCAAGC | 54.3 |
| **Gadd45g** | Forward: GAATCTTTACTTGCCCTC  Reverse: TTCTTCCAGAGTCATTGT | 56 |
| **Prkcε** | Forward: ATACTTACACTTGTGGAT  Reverse: AATAGTTCGAGACATTCT | 57.2 |
| **Prkcδ** | Forward: GTGTGTGCAGTATTTCCT  Reverse: TCATAGTTGGGAACATGG | 56.6 |
| **Mmp25** | Forward: CAAGAGGTGGATTCTCAG  Reverse: GTAACTGTCTTGGTGGTA | 56.3 |
| **Mmp3** | Forward: AATTGTTAAGAAGATCCATG  Reverse: AGAGTAAGGAAACCACTT | 55.4 |
| **Adamts5** | Forward: GCAACAGACCCAACTAAAG  Reverse: TTGCTGCTGTGGCTAATG | 56.4 |
| **Adamts7** | Forward: ACATATTCAGAGGAAGAG  Reverse: AGGAACAACTAAGACTAC | 55 |
| **Pick1** | Forward: TGGATGTGAAGTTTGAGTA  Reverse: GTATAGCGGCTCTCCTAG | 57 |
| **Grip1** | Forward: GAAGCAAGAAATCAAGGA  Reverse: TGCCAGAGTCTTTGTATA | 54.7 |
| **Cacgn3** | Forward: TTTGGAGCCTTCTCTTTC  Reverse: GCTGATGCTTCTCAATATAG | 55.5 |
| **Cacgn8** | Forward: TGGAATCATTGAAACGCT  Reverse: ATGGTGGTCAGTAGTACC | 55.9 |
| **Sypl2** | Forward: TACATAGTGGTCATCTGG  Reverse: GTACATTGAGCATTCCTAA | 56.8 |
| **Snap25** | Forward: TACATAGTGGTCATCTGG  Reverse: GTACATTGAGCATTCCTAA | 54.7 |
| **Clstn2** | Forward: CAGATCAAGTGTTCAGAG  Reverse: ATGTTCCGTATCAGAGAC | 55.6 |
| **Clstn3** | Forward: TAACACCATTCAGAACGA  Reverse: AGTAGAGAAAGGCGATTC | 55.7 |
| **Dlgap** | Forward: ACATGGACCACATCCCAC  Reverse: AAATGAACAGACAGTAAGGG | 57.9 |
| **Fmr1** | Forward: GTCTTTCTGGGTAAATCACAT  Reverse: AATGGCACAGCACTTCAT | 54.9 |
| **Gria1** | Forward: GAACCATCCGTGTTTGTTCG  Reverse: TTCCTGTCTGCTCCAGTTAC | 57 |
| **Gria2** | Forward: GAAGCCTTGTGACACCATGA  Reverse: AGCCTTGCCTTGCTCCTCAT | 57 |
| **Grin1** | Forward: TACACTGCCAACTTGGCAGCT TTC  Reverse: CATGAAGACCCCTGCCATGTT3 | 58 |
| **Grin2a** | Forward: TGG CTG CCT TCA TGA TCC  Reverse: TGC AGC GCA ATT CCA TAG | 58 |
| **Grin2b** | Forward: GGA TCT ACC AGT CTA ACA TG  Reverse: GAT AGT TAG TGA TCC CAC TG | 58 |
| **Grin3a** | Forward: TCCTCTGCCACCTCAGTAA  Reverse: TCTCACTTGGCTGGCTTCT | 58 |
| **Grin3b** | Forward: AACTGTGATGACCTGAAAAC  Reverse: AAATGAGGTGTTGCCGAG | 58 |
| **Rest** | Forward: TAAGCCATGCCAGTATGA  Reverse: AACTTCTTAGCACTGTGAAC | 57 |
