# Supplementary material for: In Vitro Ischemia Triggers a Transcriptional Response to Down-Regulate Synaptic Proteins in Hippocampal Neurons
Source: PLoS One. 2014 Jun 24;9(6):e99958. doi: 10.1371/journal.pone.0099958 (PMC4069008; doi:10.1371/journal.pone.0099958)
Supplement: Table S2 — Most strongly up-regulated (A) and down-regulated (B) genes at 7 h after OGD (fold change ≥2). (DOCX) [file pone.0099958.s003.docx]

| **A. Most up-regulated genes after 7h of recovery** | | | | |
| --- | --- | --- | --- | --- |
| **Gene Symbol** | **Gene Name** | **Function** | **Fold Change 7h** | ***p*-value** |
| **Mageb3** | Melanoma antigen family B, 3 | Apoptosis | 10.82 | 0.05 |
| **Pax3** | Paired box 3 | Transcription factor | 10.65 | 0.04 |
| **LOC685371** | Hypothetical protein LOC685371 | Unknown | 10.19 | 0.03 |
| **Evc2** | Ellis van Creveld syndrome 2 homolog (human) | Organ development | 9.86 | 0.04 |
| **RGD1309808** | Similar to apolipoprotein L2, apolipoprotein L-II | Lipid transporter activity | 9.66 | 0.04 |
| **Mkx** | Mohawk homeobox | Transcription factor | 9.17 | 0.04 |
| **Mcc** | Mutated in colorectal cancers | Unknown | 9.17 | 0.04 |
| **Krt80** | Keratin 80 | Cytoskeleton | 8.68 | 0.04 |
| **Ckm** | Creatine kinase, muscle | Metabolic process | 8.50 | 0.04 |
| **Fam155b** | Family with sequence similarity 155. member B | Unknown | 8.18 | 0.03 |
| **B. Most down-regulated genes after 7h of recovery** | | | | |
| **Gene Symbol** | **Gene Name** | **Function** | **Fold Change 7h** | ***p*-value** |
| **Agtr1b** | Angiotensin II receptor, type 1b | Receptor activity | 0.21 | 0.002 |
| **Clrn1** | Clarin 1 | Cytoskeleton | 0.22 | 0.0002 |
| **Dclk3** | Doublecortin-like kinase 3 | Kinase activity | 0.23 | 0.02 |
| **RGD1305627** | Hypothetical LOC314467 | Protease inhibitor | 0.23 | 0.01 |
| **Ttc22** | Tetratricopeptide repeat domain 22 | Unknown | 0.23 | 0.01 |
| **Mmp9** | Matrix metallopeptidase 9 | Extracellular matrix | 0.23 | 0.03 |
| **Nostrin** | Nitric oxide synthase trafficker | DNA binding | 0.24 | 0.03 |
| **Cga** | Glycoprotein hormones, alpha polypeptide | Hormone activity | 0.25 | 0.02 |
| **Ccl1** | Chemokine (C-C motif) ligand 1 | Inflammation | 0.25 | 0.04 |
| **RGD1562683** | Rgd1562683 | Unknown | 0.25 | 0.03 |
