# Supplementary material for: In Vitro Ischemia Triggers a Transcriptional Response to Down-Regulate Synaptic Proteins in Hippocampal Neurons
Source: PLoS One. 2014 Jun 24;9(6):e99958. doi: 10.1371/journal.pone.0099958 (PMC4069008; doi:10.1371/journal.pone.0099958)
Supplement: Table S3 — Most strongly up-regulated (A) and down-regulated (B) genes at 24 h after OGD (fold change ≥2). (DOCX) [file pone.0099958.s004.docx]

| **A. Most up-regulated genes after 24h of recovery** | | | | |
| --- | --- | --- | --- | --- |
| **Gene Symbol** | **Gene Name** | **Function** | **Fold change 24h** | ***p*-value** |
| **Eif4g2** | Eukaryotic translation initiation factor 4, gamma 2 | Translation initiation factor | 12.23 | 0.005 |
| **Arhgap24** | Rho gtpase activating protein 24 | Enzyme | 11.98 | 0.001 |
| **Slfn2** | Schlafen 2 | Cell cycle | 11.20 | 0.02 |
| **LOC100365145** | Hypothetical protein LOC100365145 | Unknown | 10.47 | 0.05 |
| **Ctgf** | Connective tissue growth factor | Growth factor activity | 9.84 | 0.01 |
| **Cxcl2** | Chemokine (C-X-C motif) ligand 2 | Inflammation | 8.61 | 0.05 |
| **S100a6** | S100 calcium binding protein A6 | Calcium binding | 7.79 | 0.03 |
| **Cxcl10** | Chemokine (C-X-C motif) ligand 10 | Inflammation | 7.57 | 0.02 |
| **Ptgr1** | Prostaglandin reductase 1 | Apoptosis | 7.03 | 0.03 |
| **Hmga2** | High mobility group AT-hook 2 | Transcription | 6.99 | 0.02 |
| **B. Most down-regulated genes after 24h of recovery** | | | | |
| **Gene Symbol** | **Gene Name** | **Function** | **Fold change 24h** | ***p*-value** |
| **RGD1559536** | Similar to vitellogenin-like 1 precursor | Protease inhibitor | 0.21 | 0.04 |
| **Ifi203** | Interferon activated gene 203 | Transcription factor | 0.24 | 0.05 |
| **Agbl1** | ATP/GTP binding protein-like 1 | Signaling | 0.27 | 0.02 |
| **Gpr83** | G protein-coupled receptor 83 | Receptor activity | 0.30 | 0.03 |
| **F2** | Coagulation factor II (thrombin) | Protease activity | 0.30 | 0.01 |
| **Cd27** | CD27 molecule | Apoptosis | 0.31 | 0.04 |
| **Hapln1** | Hyaluronan and proteoglycan link protein 1 | Extracellular matrix | 0.32 | 0.01 |
| **Grin1** | Glutamate receptor, ionotropic, N-methyl D-aspartate 1 | Receptor activity | 0.39 | 0.04 |
| **LOC499418** | Similar to Putative protein c21orf56 homolog | Unknown | 0.40 | 0.02 |
| **Wdr63** | WD repeat domain 63 | Cytoskeleton | 0.40 | 0.01 |
