# Supplementary material for: In Vitro Ischemia Triggers a Transcriptional Response to Down-Regulate Synaptic Proteins in Hippocampal Neurons
Source: PLoS One. 2014 Jun 24;9(6):e99958. doi: 10.1371/journal.pone.0099958 (PMC4069008; doi:10.1371/journal.pone.0099958)
Supplement: Table S4 — Most strongly up-regulated (A) and down-regulated (B) genes at both recovery periods (7 h and 24 h) after OGD (fold change ≥2). (DOCX) [file pone.0099958.s005.docx]

| **A. Most up-regulated genes at both periods of recovery (7h and 24h) after OGD** | | | | | | |
| --- | --- | --- | --- | --- | --- | --- |
| **Gene Symbol** | **Gene Name** | **Function** | **Fold change 7h** | ***p*-value** | **Fold change 24h** | ***p*-value** |
| **Il1rl1** | Interleukin 1 receptor-like 1 | Inflammation | 13.01 | 0.03 | 47.02 | 0.001 |
| **Reg3b** | Regenerating islet-derived 3 beta | Inflammation | 17.43 | 0.01 | 29.00 | 0.01 |
| **Tfpi2** | Tissue factor pathway inhibitor 2 | Enzyme inhibitor | 7.24 | 0.05 | 22.64 | 0.01 |
| **Cxcl1** | Chemokine (C-X-C motif) ligand 1 (melanoma growth stimulating activity, alpha) | Inflammation | 8.35 | 0.002 | 11.99 | 0.01 |
| **Gal** | Galanin prepropeptide | Hormone activity | 9.39 | 0.004 | 5.60 | 0.03 |
| **Serpine1** | Serpine peptidase inhibitor. clade E (nexin. plasminogen activator inhibitor type 1), member1 | Protease inhibitor | 5.03 | 0.02 | 9.77 | 0.03 |
| **B. Most down-regulated genes at both periods of recovery (7h and 24h) after OGD** | | | | | | |
| **Gene Symbol** | **Gene Name** | **Function** | **Fold change 7h** | ***p*-value** | **Fold change 24h** | ***p*-value** |
| **Mmp28** | Matrix metallopeptidase 28 | Extracellular matrix | 0.20 | 0.02 | 0.24 | 0.01 |
| **RGD1559748** | Similar to Palate lung and nasal carcinoma-like protein precursor (Tongue plunc-like protein) | Unknown | 0.29 | 0.001 | 0.25 | 0.01 |
| **Rasgrp1** | RAS guanyl releasing protein 1 (calcium and DAG-regulated) | Enzyme activity | 0.25 | 0.003 | 0.30 | 0.03 |
| **LOC100360071** | Neuropeptide S-like | Signaling | 0.19 | 0.0002 | 0.38 | 0.05 |
| **C1qtnf1** | C1q and tumor necrosis factor related protein 1 | Extracellular matrix | 0.28 | 0.002 | 0.30 | 0.01 |
| **Ube2ql1** | Ubiquitin-conjugating enzyme E2Q family-like 1 | Metabolic process | 0.31 | 0.01 | 0.36 | 0.04 |
