# Supplementary material for: In Vitro Ischemia Triggers a Transcriptional Response to Down-Regulate Synaptic Proteins in Hippocampal Neurons
Source: PLoS One. 2014 Jun 24;9(6):e99958. doi: 10.1371/journal.pone.0099958 (PMC4069008; doi:10.1371/journal.pone.0099958)
Supplement: Table S5 — List of genes up-regulated and down-regulated at 7 h after OGD for different ontological classes. Gene ontology analyses included genes that had a p-value <0.05 and a fold change of 2.0 and were performed using GoMiner. Classes were selected manually. Note that some genes are included in more than one class. (DOCX) [file pone.0099958.s006.docx]

| **Apoptosis** | **Up-regulated** | **Gene Symbol** | **Gene name** | **Accession Number** | **Fold Change 7h** | ***p*-value** |
| --- | --- | --- | --- | --- | --- | --- |
|  |  | **Tcf7l2** | Transcription factor 7-like 2 (T-cell specific, HMG-box) | NM_001191052 | 7.39 | 0.050 |
|  |  | **Il6** | Interleukin 6 | NM_012589 | 7.19 | 0.022 |
|  |  | **Bmp2** | Bone morphogenetic protein 2 | NM_017178 | 4.00 | 0.003 |
|  |  | **Hspa1b** | Heat shock 70kd protein 1B (mapped) | NM_212504 | 3.05 | 0.044 |
|  |  | **Adm** | Adrenomedullin | NM_012715 | 2.67 | 0.029 |
|  |  | **Igfbp3** | Insulin-like growth factor binding protein 3 | NM_012588 | 2.44 | 0.000 |
|  |  | **Wwox** | WW domain-containing oxidoreductase | NM_001106188 | 2.38 | 0.038 |
|  |  | **Myc** | Myelocytomatosis oncogene | NM_012603 | 2.38 | 0.014 |
|  |  | **Sox7** | SRY (sex determining region Y)-box 7 | NM_001106045 | 2.37 | 0.044 |
|  |  | **Pvr** | Poliovirus receptor | NM_017076 | 2.33 | 0.021 |
|  |  | **Cd44** | Cd44 molecule | NM_012924 | 2.28 | 0.041 |
|  |  | **Tgfb1** | Transforming growth factor, beta 1 | NM_021578 | 2.27 | 0.047 |
|  |  | **Set** | SET nuclear oncogene | NM_001012504 | 2.13 | 0.007 |
|  |  | **Gadd45g** | Growth arrest and DNA-damage-inducible, gamma | NM_001077640 | 2.08 | 0.014 |
|  |  | **Cx3cr1** | Chemokine (C-X3-C motif) receptor 1 | NM_133534 | 2.06 | 0.041 |
|  |  | **Sin3a** | SIN3 homolog A, transcription regulator (yeast) | NM_001108761 | 2.03 | 0.004 |
|  |  | **Ppp1r15a** | Protein phosphatase 1, regulatory (inhibitor) subunit 15A | NM_133546 | 2.02 | 0.035 |
|  |  | **Hmgb1** | High mobility group box 1 | NM_012963 | 2.00 | 0.009 |
|  |  | | | | | |
|  | **Down-regulated** | **Gene Symbol** | **Gene name** | **Accession Number** | **Fold Change 7h** | ***p*-value** |
|  |  | **Agtr1b** | Angiotensin II receptor, type 1b | NM_031009 | 0.21 | 0.002 |
|  |  | **Mmp9** | Matrix metallopeptidase 9 | NM_031055 | 0.23 | 0.027 |
|  |  | **Tp63** | Tumor protein p63 | NM_019221 | 0.25 | 0.000 |
|  |  | **Pou4f3** | POU class 4 homeobox 3 | NM_001108889 | 0.26 | 0.013 |
|  |  | **Cidec** | Cell death-inducing DFFA-like effector c | NM_001024333 | 0.32 | 0.014 |
|  |  | **Il4** | Interleukin 4 | NM_201270 | 0.34 | 0.002 |
|  |  | **Esr1** | Estrogen receptor 1 | NM_012689 | 0.34 | 0.002 |
|  |  | **Fgf8** | Fibroblast growth factor 8 | NM_133286 | 0.35 | 0.004 |
|  |  | **Prkcz** | Protein kinase C, zeta | NM_022507 | 0.37 | 0.012 |
|  |  | **Adcyap1** | Adenylate cyclase activating polypeptide 1 | NM_016989 | 0.40 | 0.002 |
|  |  | **Casp8** | Caspase 8 | NM_022277 | 0.40 | 0.028 |
|  |  | **Lck** | Lymphocyte-specific protein tyrosine kinase | NM_001100709 | 0.41 | 0.006 |
|  |  | **Dbh** | Dopamine beta-hydroxylase (dopamine beta-monooxygenase) | NM_013158 | 0.41 | 0.046 |
|  |  | **Foxl2** | Forkhead box L2 | ENSRNOT00000023091 | 0.44 | 0.004 |
|  |  | **Ache** | Acetylcholinesterase | NM_172009 | 0.44 | 0.015 |
|  |  | **Snca** | Synuclein, alpha (non A4 component of amyloid precursor) | S73008 | 0.45 | 0.030 |
|  |  | **Madd** | MAP-kinase activating death domain | NM_053585 | 0.46 | 0.019 |
|  |  | **Apbb1** | Amyloid beta (A4) precursor protein-binding, family B, member 1 (Fe65) | NM_080478 | 0.47 | 0.017 |
|  |  | **Plekhg5** | Pleckstrin homology domain containing, family G (with rhogef domain) member 5 | NM_201272 | 0.48 | 0.003 |
|  | | | | | | |
| **Inflammatory Response** | **Up-regulated** | **Gene Symbol** | **Gene name** | **Accession Number** | **Fold Change 7h** | ***p*-value** |
|  |  | **Il6** | Interleukin 6 | NM_012589 | 7.19 | 0.022 |
|  |  | **Bmp2** | Bone morphogenetic protein 2 | NM_017178 | 4.00 | 0.003 |
|  |  | **Cd55** | Cd55 molecule | NM_022269 | 3.79 | 0.011 |
|  |  | **Itgb6** | Integrin, beta 6 | NM_001004263 | 3.75 | 0.003 |
|  |  | **Tlr2** | Toll-like receptor 2 | NM_198769 | 3.58 | 0.005 |
|  |  | **Cd44** | Cd44 molecule | NM_012924 | 2.28 | 0.041 |
|  |  | **Tgfb1** | Transforming growth factor, beta 1 | NM_021578 | 2.27 | 0.047 |
|  |  | **Hmgb1** | High mobility group box 1 | NM_012963 | 2.00 | 0.009 |
|  |  | | | | | |
|  | **Down-regulated** | **Gene Symbol** | **Gene name** | **Accession Number** | **Fold Change 7h** | ***p*-value** |
|  |  | **Ptafr** | Platelet-activating factor receptor | NM_053321 | 0.30 | 0.025 |
|  |  | **Il4** | Interleukin 4 | NM_201270 | 0.34 | 0.002 |
|  |  | **Afap1l2** | Actin filament associated protein 1-like 2 | XM_001064140 | 0.36 | 0.002 |
|  |  | **Adcyap1** | Adenylate cyclase activating polypeptide 1 | NM_016989 | 0.40 | 0.002 |
|  |  | **Masp1** | Mannan-binding lectin serine peptidase 1 | NM_022257 | 0.40 | 0.024 |
|  |  | **Ache** | Acetylcholinesterase | NM_172009 | 0.44 | 0.015 |
|  |  | **F12** | Coagulation factor XII (Hageman factor) | NM_001014006 | 0.45 | 0.011 |
|  |  | **Tac1** | Tachykinin 1 | NM_012666 | 0.49 | 0.010 |
|  |  | **Itgb2** | Integrin, beta 2 | NM_001037780 | 0.49 | 0.035 |
|  |  | **Tnfrsf11a** | Tumor necrosis factor receptor superfamily, member 11a | XM_001063501 | 0.49 | 0.005 |
|  | | | | | | |
| **Ion Transmembr.**  **Transporter Activity** | **Up-regulated** | **Gene Symbol** | **Gene name** | **Accession Number** | **Fold Change 7h** | ***p*-value** |
|  |  | **Slc26a3** | Solute carrier family 26, member 3 | NM_053755 | 4.34 | 0.013 |
|  |  | **Kcnj8** | Potassium inwardly-rectifying channel, subfamily J, member 8 | NM_017099 | 3.32 | 0.032 |
|  |  | **Slc1a5** | Solute carrier family 1 (neutral amino acid transporter), member 5 | NM_175758 | 2.59 | 0.007 |
|  |  | **Slc15a3** | Solute carrier family 15, member 3 | NM_139341 | 2.35 | 0.031 |
|  |  | **Glra2** | Glycine receptor, alpha 2 | NM_012568 | 2.24 | 0.028 |
|  |  | | | | | |
|  | **Down-regulated** | **Gene Symbol** | **Gene name** | **Accession Number** | **Fold Change 7h** | ***p*-value** |
|  |  | **Trpc7** | Transient receptor potential cation channel, subfamily C, member 7 | NM_001191691 | 0.34 | 0.031 |
|  |  | **Kcnj4** | Potassium inwardly-rectifying channel, subfamily J, member 4 | NM_053870 | 0.36 | 0.012 |
|  |  | **Trpc6** | Transient receptor potential cation channel, subfamily C, member 6 | NM_053559 | 0.37 | 0.009 |
|  |  | **Chrng** | Cholinergic receptor, nicotinic, gamma | NM_019145 | 0.38 | 0.035 |
|  |  | **Cacna2d3** | Calcium channel, voltage-dependent, alpha2/delta subunit 3 | NM_175595 | 0.40 | 0.028 |
|  |  | **Atp9b** | Atpase, class II, type 9B | NM_001106130 | 0.40 | 0.008 |
|  |  | **Slc13a1** | Solute carrier family 13 (sodium/sulfate symporters), member 1 | NM_031651 | 0.40 | 0.026 |
|  |  | **Atp5d** | ATP synthase, H+ transporting, mitochondrial F1 complex, delta subunit | BC161836 | 0.41 | 0.000 |
|  |  | **Cacng3** | Calcium channel, voltage-dependent, gamma subunit 3 | NM_080691 | 0.41 | 0.009 |
|  |  | **Clic3** | Chloride intracellular channel 3 | NM_001013080 | 0.42 | 0.039 |
|  |  | **Nox1** | NADPH oxidase 1 | NM_053683 | 0.43 | 0.002 |
|  |  | **Snap25** | Synaptosomal-associated protein 25 | NM_030991 | 0.43 | 0.027 |
|  |  | **Chrna4** | Cholinergic receptor, nicotinic, alpha 4 | NM_024354 | 0.43 | 0.025 |
|  |  | **Slc30a2** | Solute carrier family 30 (zinc transporter), member 2 | NM_001083122 | 0.44 | 0.005 |
|  |  | **Cftr** | Cystic fibrosis transmembrane conductance regulator homolog (human) | NM_031506 | 0.44 | 0.013 |
|  |  | **Svop** | SV2 related protein | NM_134404 | 0.44 | 0.033 |
|  |  | **Slc6a5** | Solute carrier family 6 (neurotransmitter transporter, glycine), member 5 | NM_203334 | 0.45 | 0.021 |
|  |  | **Cacnb2** | Calcium channel, voltage-dependent, beta 2 subunit | NM_053851 | 0.45 | 0.003 |
|  |  | **Slc17a7** | Solute carrier family 17 (sodium-dependent inorganic phosphate cotransporter), member 7 | NM_053859 | 0.45 | 0.022 |
|  |  | **Atp6v0a4** | Atpase, H+ transporting, lysosomal V0 subunit A4 | NM_001106591 | 0.46 | 0.028 |
|  |  | **Kcnk6** | Potassium channel, subfamily K, member 6 | NM_053806 | 0.46 | 0.009 |
|  |  | **Kcns1** | Potassium voltage-gated channel, delayed-rectifier, subfamily S, member 1 | NM_053954 | 0.46 | 0.042 |
|  |  | **Kctd2** | Potassium channel tetramerisation domain containing 2 | XM_001081684 | 0.48 | 0.023 |
|  |  | **Trpm2** | Transient receptor potential cation channel, subfamily M, member 2 | NM_001011559 | 0.49 | 0.002 |
|  | | | | | | |
| **Metabolic Process** | **Up-regulated** | **Gene Symbol** | **Gene name** | **Accession Number** | **Fold Change 7h** | ***p*-value** |
|  |  | **Pax3** | Paired box 3 | NM_053710 | 10.65 | 0.044 |
|  |  | **Rgd1309808** | Similar to apolipoprotein L2; apolipoprotein L-II | NM_001134801 | 9.66 | 0.042 |
|  |  | **Mkx** | Mohawk homeobox | ENSRNOT00000025623 | 9.17 | 0.040 |
|  |  | **Ckm** | Creatine kinase, muscle | NM_012530 | 8.50 | 0.035 |
|  |  | **Tcf7l2** | Transcription factor 7-like 2 (T-cell specific, HMG-box) | NM_001191052 | 7.39 | 0.050 |
|  |  | **Il6** | Interleukin 6 | NM_012589 | 7.19 | 0.022 |
|  |  | **Ereg** | Epiregulin | NM_021689 | 6.11 | 0.002 |
|  |  | **Fst** | Follistatin | NM_012561 | 4.89 | 0.020 |
|  |  | **Nr4a3** | Nuclear receptor subfamily 4, group A, member 3 | NM_017352 | 4.54 | 0.003 |
|  |  | **Dio3** | Deiodinase, iodothyronine, type III | NM_017210 | 4.47 | 0.003 |
|  |  | **Adamts1** | ADAM metallopeptidase with thrombospondin type 1 motif, 1 | NM_024400 | 4.32 | 0.001 |
|  |  | **Ucn2** | Urocortin 2 | NM_133385 | 4.06 | 0.018 |
|  |  | **Rgd1561667** | Similar to putative protein kinase | XM_001054195 | 4.05 | 0.026 |
|  |  | **Rgd1565390** | Similar to putative protein kinase | XM_344843 | 3.28 | 0.018 |
|  |  | **Bmp2** | Bone morphogenetic protein 2 | NM_017178 | 4.00 | 0.003 |
|  |  | **Runx1** | Runt-related transcription factor 1 | NM_017325 | 3.90 | 0.000 |
|  |  | **Alpl** | Alkaline phosphatase, liver/bone/kidney | NM_013059 | 3.89 | 0.000 |
|  |  | **Car13** | Carbonic anhydrase 13 | NM_001134993 | 3.81 | 0.048 |
|  |  | **Cd55** | Cd55 molecule | NM_022269 | 3.79 | 0.011 |
|  |  | **Loc365499** | Similar to KP78b CG17216-PA | XM_001053352 | 3.60 | 0.050 |
|  |  | **Tlr2** | Toll-like receptor 2 | NM_198769 | 3.58 | 0.005 |
|  |  | **Map3k8** | Mitogen-activated protein kinase kinase kinase 8 | NM_053847 | 3.43 | 0.001 |
|  |  | **Phlda1** | Pleckstrin homology-like domain, family A, member 1 | NM_017180 | 3.13 | 0.008 |
|  |  | **Plaur** | Plasminogen activator, urokinase receptor | NM_134352 | 3.13 | 0.006 |
|  |  | **Loc689299** | Similar to serine/threonine kinase | XM_001070335 | 3.08 | 0.049 |
|  |  | **Areg** | Amphiregulin | NM_017123 | 3.07 | 0.017 |
|  |  | **Hspa1b** | Heat shock 70kd protein 1B (mapped) | NM_212504 | 3.05 | 0.044 |
|  |  | **Loc681776** | Similar to High mobility group protein 1 (HMG-1) (High mobility group protein B1) (Amphoterin) (Heparin-binding protein p30) | XM_002725357 | 3.03 | 0.018 |
|  |  | **Loc681117** | Similar to ribosomal protein L6 | XM_001060376 | 3.03 | 0.026 |
|  |  | **Sfrs11** | Splicing factor, arginine/serine-rich 11 | NM_001035255 | 2.99 | 0.031 |
|  |  | **Batf3** | Basic leucine zipper transcription factor, ATF-like 3 | NM_021865 | 2.95 | 0.005 |
|  |  | **Uncx** | UNC homeobox | NM_017179 | 2.90 | 0.001 |
|  |  | **Usp36** | Ubiquitin specific peptidase 36 | NM_001107069 | 2.88 | 0.023 |
|  |  | **Foxe3** | Forkhead box E3 | ENSRNOT00000010208 | 2.85 | 0.017 |
|  |  | **Hlx** | H2.0-like homeobox | NM_001077674 | 2.83 | 0.000 |
|  |  | **Hmga1** | High mobility group AT-hook 1 | NM_139327 | 2.82 | 0.011 |
|  |  | **Sgsm1** | Small G protein signaling modulator 1 | ENSRNOT00000056824 | 2.81 | 0.038 |
|  |  | **Adm** | Adrenomedullin | NM_012715 | 2.67 | 0.029 |
|  |  | **Nasp** | Nuclear autoantigenic sperm protein (histone-binding) | NM_001005543 | 2.60 | 0.042 |
|  |  | **Pax1** | Paired box 1 | NM_001107787 | 2.59 | 0.030 |
|  |  | **Hbegf** | Heparin-binding EGF-like growth factor | NM_012945 | 2.56 | 0.010 |
|  |  | **Prss23** | Protease, serine, 23 | NM_001007691 | 2.49 | 0.037 |
|  |  | **Erc1** | ELKS/RAB6-interacting/CAST family member 1 | NM_170788 | 2.47 | 0.033 |
|  |  | **Taok3** | TAO kinase 3 | NM_001024254 | 2.46 | 0.036 |
|  |  | **Igfbp3** | Insulin-like growth factor binding protein 3 | NM_012588 | 2.44 | 0.000 |
|  |  | **Rnf144b** | Ring finger protein 144B | NM_001108881 | 2.43 | 0.006 |
|  |  | **Hdc** | Histidine decarboxylase | NM_017016 | 2.40 | 0.027 |
|  |  | **Wnt6** | Wingless-type MMTV integration site family, member 6 | NM_001108226 | 2.39 | 0.048 |
|  |  | **Wwox** | WW domain-containing oxidoreductase | NM_001106188 | 2.38 | 0.038 |
|  |  | **Myc** | Myelocytomatosis oncogene | NM_012603 | 2.38 | 0.014 |
|  |  | **Sox7** | SRY (sex determining region Y)-box 7 | NM_001106045 | 2.37 | 0.044 |
|  |  | **Eid3** | EP300 interacting inhibitor of differentiation 3 | NM_001044304 | 2.37 | 0.027 |
|  |  | **Tgm1** | Transglutaminase 1, K polypeptide | NM_031659 | 2.36 | 0.022 |
|  |  | **Slc15a3** | Solute carrier family 15, member 3 | NM_139341 | 2.35 | 0.031 |
|  |  | **Fmr1** | Fragile X mental retardation 1 | NM_052804 | 2.34 | 0.027 |
|  |  | **Npm3** | Nucleophosmin/nucleoplasmin, 3 | ENSRNOT00000023963 | 2.29 | 0.012 |
|  |  | **Ptx3** | Pentraxin related gene | NM_001109536 | 2.29 | 0.023 |
|  |  | **Cd44** | Cd44 molecule | NM_012924 | 2.28 | 0.041 |
|  |  | **Tgfb1** | Transforming growth factor, beta 1 | NM_021578 | 2.27 | 0.047 |
|  |  | **Junb** | Jun B proto-oncogene | NM_021836 | 2.26 | 0.001 |
|  |  | **Ctrc** | Chymotrypsin C (caldecrin) | NM_001077649 | 2.25 | 0.039 |
|  |  | **Rgd1561672** | Similar to novel protein | XM_001065540 | 2.24 | 0.014 |
|  |  | **Klf5** | Kruppel-like factor 5 | NM_053394 | 2.23 | 0.012 |
|  |  | **Rgd1564342** | Similar to hypothetical protein FLJ32685 | ENSRNOT00000030991 | 2.21 | 0.044 |
|  |  | **Atf3** | Activating transcription factor 3 | NM_012912 | 2.20 | 0.021 |
|  |  | **Rxfp3** | Relaxin/insulin-like family peptide receptor 3 | NM_001008310 | 2.15 | 0.018 |
|  |  | **Fzd6** | Frizzled homolog 6 (Drosophila) | NM_001130536 | 2.15 | 0.005 |
|  |  | **Zfp217** | Zinc finger protein 217 | NM_001107813 | 2.14 | 0.003 |
|  |  | **Cym** | Chymosin | NM_020091 | 2.11 | 0.038 |
|  |  | **Pdgfc** | Platelet derived growth factor C | NM_031317 | 2.11 | 0.044 |
|  |  | **Gtpbp4** | GTP binding protein 4 | NM_053689 | 2.09 | 0.003 |
|  |  | **Gadd45g** | Growth arrest and DNA-damage-inducible, gamma | NM_001077640 | 2.08 | 0.014 |
|  |  | **Hsp90aa1** | Heat shock protein 90, alpha (cytosolic), class A member 1 | NM_175761 | 2.07 | 0.018 |
|  |  | **Sbk2** | SH3-binding domain kinase family, member 2 | NM_001127539 | 2.04 | 0.023 |
|  |  | **Sin3a** | SIN3 homolog A, transcription regulator (yeast) | NM_001108761 | 2.03 | 0.004 |
|  |  | **Ppp1r15a** | Protein phosphatase 1, regulatory (inhibitor) subunit 15A | NM_133546 | 2.02 | 0.035 |
|  |  | **Hmgb1** | High mobility group box 1 | NM_012963 | 2.00 | 0.009 |
|  |  | | | | | |
|  | **Down-regulated** | **Gene Symbol** | **Gene name** | **Accession Number** | **Fold Change 7h** | ***p*-value** |
|  |  | **Hes5** | Hairy and enhancer of split 5 (Drosophila) | NM_024383 | 0.18 | 0.033 |
|  |  | **Tgm5** | Transglutaminase 5 | XM_001080153 | 0.20 | 0.001 |
|  |  | **Mmp9** | Matrix metallopeptidase 9 | NM_031055 | 0.23 | 0.027 |
|  |  | **Nostrin** | Nitric oxide synthase trafficker | NM_001024260 | 0.24 | 0.029 |
|  |  | **Cga** | Glycoprotein hormones, alpha polypeptide | NM_053918 | 0.25 | 0.024 |
|  |  | **Cdkn1c** | Cyclin-dependent kinase inhibitor 1C | NM_182735 | 0.25 | 0.005 |
|  |  | **Tp63** | Tumor protein p63 | NM_019221 | 0.25 | 0.000 |
|  |  | **Pou4f3** | POU class 4 homeobox 3 | NM_001108889 | 0.26 | 0.013 |
|  |  | **Adam2** | ADAM metallopeptidase domain 2 | NM_020077 | 0.26 | 0.001 |
|  |  | **Tmprss11d** | Transmembrane protease, serine 11d | NM_022630 | 0.27 | 0.027 |
|  |  | **Adamdec1** | ADAM-like, decysin 1 | NM_001106046 | 0.28 | 0.003 |
|  |  | **Aicda** | Activation-induced cytidine deaminase | NM_001100779 | 0.29 | 0.015 |
|  |  | **Ptafr** | Platelet-activating factor receptor | NM_053321 | 0.30 | 0.025 |
|  |  | **Cpxm2** | Carboxypeptidase X (M14 family), member 2 | NM_001106306 | 0.31 | 0.029 |
|  |  | **Cyp27a1** | Cytochrome P450, family 27, subfamily a, polypeptide 1 | NM_178847 | 0.32 | 0.025 |
|  |  | **Neu4** | Sialidase 4 | NM_001108234 | 0.32 | 0.020 |
|  |  | **Htr4** | 5-hydroxytryptamine (serotonin) receptor 4 | NM_012853 | 0.32 | 0.002 |
|  |  | **Cyp11b1** | Cytochrome P450, family 11, subfamily b, polypeptide 1 | NM_012537 | 0.32 | 0.003 |
|  |  | **Tyrp1** | Tyrosinase-related protein 1 | NM_001106664 | 0.33 | 0.012 |
|  |  | **Tbx1** | T-box 1 | NM_001108322 | 0.33 | 0.037 |
|  |  | **Fancd2** | Fanconi anemia, complementation group D2 | NM_001001719 | 0.34 | 0.044 |
|  |  | **Il4** | Interleukin 4 | NM_201270 | 0.34 | 0.002 |
|  |  | **Esr1** | Estrogen receptor 1 | NM_012689 | 0.34 | 0.002 |
|  |  | **Fgf8** | Fibroblast growth factor 8 | NM_133286 | 0.35 | 0.004 |
|  |  | **Pcsk2** | Proprotein convertase subtilisin/kexin type 2 | NM_012746 | 0.35 | 0.020 |
|  |  | **Rgd1304879** | Similar to Zinc finger protein 398 (Zinc finger DNA binding protein p52/p71) | NM_001014056 | 0.35 | 0.004 |
|  |  | **Uox** | Urate oxidase | NM_053768 | 0.36 | 0.030 |
|  |  | **Ctsll3** | Cathepsin L-like 3 | ENSRNOT00000061398 | 0.36 | 0.002 |
|  |  | **Afap1l2** | Actin filament associated protein 1-like 2 | XM_001064140 | 0.36 | 0.002 |
|  |  | **Acan** | Aggrecan | NM_022190 | 0.36 | 0.036 |
|  |  | **Camkk1** | Calcium/calmodulin-dependent protein kinase kinase 1, alpha | NM_031662 | 0.36 | 0.004 |
|  |  | **Prkcz** | Protein kinase C, zeta | NM_022507 | 0.37 | 0.012 |
|  |  | **Gstm6** | Glutathione S-transferase, mu 6 | NM_001109192 | 0.38 | 0.007 |
|  |  | **Pkm2** | Pyruvate kinase, muscle | NM_053297 | 0.38 | 0.035 |
|  |  | **Adcyap1** | Adenylate cyclase activating polypeptide 1 | NM_016989 | 0.40 | 0.002 |
|  |  | **Acot12** | Acyl-coa thioesterase 12 | NM_130747 | 0.40 | 0.033 |
|  |  | **Masp1** | Mannan-binding lectin serine peptidase 1 | NM_022257 | 0.40 | 0.024 |
|  |  | **Casp8** | Caspase 8 | NM_022277 | 0.40 | 0.028 |
|  |  | **Atp9b** | Atpase, class II, type 9B | NM_001106130 | 0.40 | 0.008 |
|  |  | **Gna15** | Guanine nucleotide binding protein, alpha 15 | NM_053542 | 0.40 | 0.001 |
|  |  | **Hoxc6** | Homeo box C6 | XM_001069410 | 0.40 | 0.015 |
|  |  | **Dmrtc1a** | DMRT-like family c1a | NM_001025288 | 0.40 | 0.008 |
|  |  | **Atp5d** | ATP synthase, H+ transporting, mitochondrial F1 complex, delta subunit | BC161836 | 0.41 | 0.000 |
|  |  | **Dclk3** | Doublecortin-like kinase 3 | NM_001191800 | 0.41 | 0.000 |
|  |  | **Lck** | Lymphocyte-specific protein tyrosine kinase | NM_001100709 | 0.41 | 0.006 |
|  |  | **Dbh** | Dopamine beta-hydroxylase (dopamine beta-monooxygenase) | NM_013158 | 0.41 | 0.046 |
|  |  | **P4htm** | Prolyl 4-hydroxylase, transmembrane | ENSRNOT00000027466 | 0.41 | 0.013 |
|  |  | **Plagl1** | Pleiomorphic adenoma gene-like 1 | NM_012760 | 0.41 | 0.015 |
|  |  | **Mas1** | MAS1 oncogene | NM_012757 | 0.42 | 0.030 |
|  |  | **Calcr** | Calcitonin receptor | NM_053816 | 0.42 | 0.040 |
|  |  | **Prmt8** | Protein arginine methyltransferase 8 | XM_002726433 | 0.42 | 0.023 |
|  |  | **Nox1** | NADPH oxidase 1 | NM_053683 | 0.43 | 0.002 |
|  |  | **Wnt3** | Wingless-type MMTV integration site family, member 3 | NM_001105715 | 0.43 | 0.003 |
|  |  | **Chrna4** | Cholinergic receptor, nicotinic, alpha 4 | NM_024354 | 0.43 | 0.025 |
|  |  | **Hcfc1** | Host cell factor C1 | NM_001139507 | 0.43 | 0.005 |
|  |  | **Foxl2** | Forkhead box L2 | ENSRNOT00000023091 | 0.44 | 0.004 |
|  |  | **Egr4** | Early growth response 4 | NM_019137 | 0.44 | 0.008 |
|  |  | **Cftr** | Cystic fibrosis transmembrane conductance regulator homolog (human) | NM_031506 | 0.44 | 0.013 |
|  |  | **Ache** | Acetylcholinesterase | NM_172009 | 0.44 | 0.015 |
|  |  | **Htr2c** | 5-hydroxytryptamine (serotonin) receptor 2C | NM_012765 | 0.44 | 0.041 |
|  |  | **Fut7** | Fucosyltransferase 7 (alpha (1,3) fucosyltransferase) | NM_199491 | 0.44 | 0.024 |
|  |  | **Gal3st2** | Galactose-3-O-sulfotransferase 2 | XM_001063652 | 0.44 | 0.036 |
|  |  | **Adra2a** | Adrenergic, alpha-2A-, receptor | NM_012739 | 0.44 | 0.040 |
|  |  | **Acot5** | Acyl-coa thioesterase 5 | NM_001079709 | 0.44 | 0.024 |
|  |  | **Socs7** | Suppressor of cytokine signaling 7 | XM_213443 | 0.45 | 0.004 |
|  |  | **Nppa** | Natriuretic peptide precursor A | NM_012612 | 0.45 | 0.014 |
|  |  | **Snca** | Synuclein, alpha (non A4 component of amyloid precursor) | S73008 | 0.45 | 0.030 |
|  |  | **F12** | Coagulation factor XII (Hageman factor) | NM_001014006 | 0.45 | 0.011 |
|  |  | **Loc680273** | Similar to Forkhead box protein L1 (Forkhead-related protein FKHL11) (Forkhead-related transcription factor 7) (FREAC-7) | XM_001056413 | 0.45 | 0.036 |
|  |  | **Akr1c13** | Aldo-keto reductase family 1, member C13 | NM_001014240 | 0.45 | 0.029 |
|  |  | **Lrrn3** | Leucine rich repeat neuronal 3 | NM_030856 | 0.45 | 0.043 |
|  |  | **Gucy2g** | Guanylate cyclase 2G | NM_139042 | 0.45 | 0.001 |
|  |  | **Adcy3** | Adenylate cyclase 3 | NM_130779 | 0.45 | 0.014 |
|  |  | **Gstm3** | Glutathione S-transferase mu 3 | NM_020540 | 0.46 | 0.026 |
|  |  | **Rdh5** | Retinol dehydrogenase 5 | ENSRNOT00000010217 | 0.46 | 0.028 |
|  |  | **Atp6v0a4** | Atpase, H+ transporting, lysosomal V0 subunit A4 | NM_001106591 | 0.46 | 0.028 |
|  |  | **Ckmt2** | Creatine kinase, mitochondrial 2, sarcomeric | NM_001127652 | 0.46 | 0.048 |
|  |  | **Bmx** | BMX non-receptor tyrosine kinase | NM_001109016 | 0.46 | 0.020 |
|  |  | **Madd** | MAP-kinase activating death domain | NM_053585 | 0.46 | 0.019 |
|  |  | **Tceanc** | Transcription elongation factor A (SII) N-terminal and central domain containing | NM_001109015 | 0.46 | 0.007 |
|  |  | **Dpp4** | Dipeptidylpeptidase 4 | NM_012789 | 0.46 | 0.044 |
|  |  | **Zfp112** | Zinc finger protein 112 | NM_001107487 | 0.47 | 0.005 |
|  |  | **Pcsk4** | Proprotein convertase subtilisin/kexin type 4 | NM_133559 | 0.47 | 0.006 |
|  |  | **Apbb1** | Amyloid beta (A4) precursor protein-binding, family B, member 1 (Fe65) | NM_080478 | 0.47 | 0.017 |
|  |  | **Mc4r** | Melanocortin 4 receptor | NM_013099 | 0.47 | 0.033 |
|  |  | **Dpyd** | Dihydropyrimidine dehydrogenase | NM_031027 | 0.32 | 0.031 |
|  |  | **Ugt2b36** | Dihydropyrimidine dehydrogenase | NM_031027 | 0.32 | 0.031 |
|  |  | **Plekhg5** | Pleckstrin homology domain containing, family G (with rhogef domain) member 5 | NM_201272 | 0.48 | 0.003 |
|  |  | **Zfp324** | Zinc finger protein 324 | ENSRNOT00000036874 | 0.48 | 0.009 |
|  |  | **Prlr** | Prolactin receptor | NM_012630 | 0.48 | 0.013 |
|  |  | **Sult4a1** | Sulfotransferase family 4A, member 1 | NM_031641 | 0.48 | 0.013 |
|  |  | **Rpusd3** | RNA pseudouridylate synthase domain containing 3 | NM_001108641 | 0.48 | 0.010 |
|  |  | **Tex15** | Testis expressed 15 | NM_001106087 | 0.48 | 0.046 |
|  |  | **Pick1** | Protein interacting with PRKCA 1 | NM_053460 | 0.48 | 0.018 |
|  |  | **Prlhr** | Prolactin releasing hormone receptor | NM_139193 | 0.48 | 0.002 |
|  |  | **Zcchc12** | Zinc finger, CCHC domain containing 12 | NM_001014065 | 0.48 | 0.021 |
|  |  | **Asphd2** | Aspartate beta-hydroxylase domain containing 2 | NM_001009716 | 0.48 | 0.011 |
|  |  | **Itgb2** | Integrin, beta 2 | NM_001037780 | 0.49 | 0.035 |
|  |  | **Tnfrsf11a** | Tumor necrosis factor receptor superfamily, member 11a | XM_001063501 | 0.49 | 0.005 |
|  |  | **Ece2** | Endothelin-converting enzyme 2 | NM_001002815 | 0.49 | 0.004 |
|  |  | **Ampd2** | Adenosine monophosphate deaminase 2 (isoform L) | NM_001101681 | 0.49 | 0.025 |
|  |  | **Mgc109340** | Similar to Microsomal signal peptidase 23 kda subunit (spase 22 kda subunit) (SPC22/23) | NM_001024267 | 0.49 | 0.023 |
|  |  | **Insig2** | Insulin induced gene 2 | NM_178091 | 0.49 | 0.013 |
|  |  | **Rgd1308116** | Similar to hypothetical protein MGC42105 | XM_001076547 | 0.50 | 0.022 |
|  |  | **Afp** | Alpha-fetoprotein | NM_012493 | 0.50 | 0.039 |
|  |  | **Zbtb24** | Zinc finger and BTB domain containing 24 | NM_001098667 | 0.50 | 0.013 |
|  |  | **Mstn** | Myostatin | NM_019151 | 0.50 | 0.021 |
|  |  | **Lor** | Loricrin | ENSRNOT00000056500 | 0.50 | 0.004 |
|  | | | | | | |
| **Neurotransmitter Secretion** | **Up-regulated** | **Gene Symbol** | **Gene name** | **Accession Number** | **Fold Change 7h** | ***p*-value** |
|  |  | **Unc13c** | Unc-13 homolog C (C. Elegans) | NM_173146 | 2.51 | 0.039 |
|  |  | | | | | |
|  | **Down-regulated** | **Gene Symbol** | **Gene name** | **Accession Number** | **Fold Change 7h** | ***p*-value** |
|  |  | **Unc13b** | Unc-13 homolog B (C. Elegans) | NM_001042579 | 0.33 | 0.017 |
|  |  | **Htr6** | 5-hydroxytryptamine (serotonin) receptor 6 | NM_024365 | 0.39 | 0.046 |
|  |  | **Snap25** | Synaptosomal-associated protein 25 | NM_030991 | 0.43 | 0.027 |
|  |  | **Htr2c** | 5-hydroxytryptamine (serotonin) receptor 2C | NM_012765 | 0.44 | 0.041 |
|  |  | **Snca** | Synuclein, alpha (non A4 component of amyloid precursor) | S73008 | 0.45 | 0.030 |
|  |  | **Syt3** | Synaptotagmin III | NM_019122 | 0.48 | 0.014 |
|  | | | | | | |
| **Signaling Pathways** | **Up-regulated** | **Gene Symbol** | **Gene name** | **Accession Number** | **Fold Change 7h** | ***p*-value** |
|  |  | **Mcc** | Mutated in colorectal cancers | NM_001170534 | 0.04 | 0.036 |
|  |  | **Tcf7l2** | Transcription factor 7-like 2 (T-cell specific, HMG-box) | NM_001191052 | 0.05 | 0.050 |
|  |  | **Il6** | Interleukin 6 | NM_012589 | 0.02 | 0.022 |
|  |  | **Ereg** | Epiregulin | NM_021689 | 0.00 | 0.002 |
|  |  | **Fst** | Follistatin | NM_012561 | 0.02 | 0.020 |
|  |  | **Adamts1** | ADAM metallopeptidase with thrombospondin type 1 motif, 1 | NM_024400 | 0.00 | 0.001 |
|  |  | **Bmp2** | Bone morphogenetic protein 2 | NM_017178 | 0.00 | 0.003 |
|  |  | **Itgb6** | Integrin, beta 6 | NM_001004263 | 0.00 | 0.003 |
|  |  | **Olr374** | Olfactory receptor 374 | NM_001001289 | 0.01 | 0.011 |
|  |  | **Tlr2** | Toll-like receptor 2 | NM_198769 | 0.01 | 0.005 |
|  |  | **Areg** | Amphiregulin | NM_017123 | 0.02 | 0.017 |
|  |  | **Sgsm1** | Small G protein signaling modulator 1 | ENSRNOT00000056824 | 0.04 | 0.038 |
|  |  | **Rgd1564791** | Similar to hypothetical protein 4930474N05 | XM_574052 | 0.04 | 0.041 |
|  |  | **Adm** | Adrenomedullin | NM_012715 | 0.03 | 0.029 |
|  |  | **Hbegf** | Heparin-binding EGF-like growth factor | NM_012945 | 0.01 | 0.010 |
|  |  | **Fgf18** | Fibroblast growth factor 18 | NM_019199 | 0.00 | 0.000 |
|  |  | **Erc1** | ELKS/RAB6-interacting/CAST family member 1 | NM_170788 | 0.03 | 0.033 |
|  |  | **Taok3** | TAO kinase 3 | NM_001024254 | 0.04 | 0.036 |
|  |  | **F2rl1** | Coagulation factor II (thrombin) receptor-like 1 | NM_053897 | 0.01 | 0.007 |
|  |  | **Wnt6** | Wingless-type MMTV integration site family, member 6 | NM_001108226 | 0.05 | 0.048 |
|  |  | **Wwox** | WW domain-containing oxidoreductase | NM_001106188 | 0.04 | 0.038 |
|  |  | **Olr1694** | Olfactory receptor 1694 | NM_001001110 | 0.00 | 0.001 |
|  |  | **Sox7** | SRY (sex determining region Y)-box 7 | NM_001106045 | 0.04 | 0.044 |
|  |  | **Rab20** | RAB20, member RAS oncogene family | NM_001109535 | 0.01 | 0.006 |
|  |  | **Olr1673** | Olfactory receptor 1673 | NM_001000268 | 0.01 | 0.014 |
|  |  | **Cd44** | Cd44 molecule | NM_012924 | 0.04 | 0.041 |
|  |  | **Tgfb1** | Transforming growth factor, beta 1 | NM_021578 | 0.05 | 0.047 |
|  |  | **Rnd3** | Rho family gtpase 3 | NM_001007641 | 0.04 | 0.039 |
|  |  | **Shc4** | SHC (Src homology 2 domain containing) family, member 4 | NM_001191065 | 0.00 | 0.005 |
|  |  | **Glra2** | Glycine receptor, alpha 2 | NM_012568 | 0.03 | 0.028 |
|  |  | **Bst2** | Bone marrow stromal cell antigen 2 | NM_198134 | 0.03 | 0.031 |
|  |  | **Rxfp3** | Relaxin/insulin-like family peptide receptor 3 | NM_001008310 | 0.02 | 0.018 |
|  |  | **Fzd6** | Frizzled homolog 6 (Drosophila) | NM_001130536 | 0.01 | 0.005 |
|  |  | **Pdgfc** | Platelet derived growth factor C | NM_031317 | 0.04 | 0.044 |
|  |  | **Rspo3** | R-spondin 3 homolog (Xenopus laevis) | NM_001100990 | 0.02 | 0.025 |
|  |  | **Olr803** | Olfactory receptor 803 | NM_001000853 | 0.01 | 0.009 |
|  |  | **Gadd45g** | Growth arrest and DNA-damage-inducible, gamma | NM_001077640 | 0.01 | 0.014 |
|  |  | **Cx3cr1** | Chemokine (C-X3-C motif) receptor 1 | NM_133534 | 0.04 | 0.041 |
|  |  | **Shoc2** | Soc-2 (suppressor of clear) homolog (C. Elegans) | NM_001013155 | 0.00 | 0.003 |
|  |  | **Ppp1r15a** | Protein phosphatase 1, regulatory (inhibitor) subunit 15A | NM_133546 | 0.03 | 0.035 |
|  |  | | | | | |
|  | **Down-regulated** | **Gene Symbol** | **Gene name** | **Accession Number** | **Fold Change 7h** | ***p*-value** |
|  |  | **Blnk** | B-cell linker | NM_001025767 | 0.13 | 0.003 |
|  |  | **Hes5** | Hairy and enhancer of split 5 (Drosophila) | NM_024383 | 0.18 | 0.033 |
|  |  | **Olr282** | Olfactory receptor 282 | NM_001000224 | 0.19 | 0.038 |
|  |  | **Agtr1b** | Angiotensin II receptor, type 1b | NM_031009 | 0.21 | 0.002 |
|  |  | **Cdkn1c** | Cyclin-dependent kinase inhibitor 1C | NM_182735 | 0.25 | 0.005 |
|  |  | **Tp63** | Tumor protein p63 | NM_019221 | 0.25 | 0.000 |
|  |  | **Ptafr** | Platelet-activating factor receptor | NM_053321 | 0.30 | 0.025 |
|  |  | **Gpr6** | G protein-coupled receptor 6 | NM_031806 | 0.31 | 0.003 |
|  |  | **Htr4** | 5-hydroxytryptamine (serotonin) receptor 4 | NM_012853 | 0.32 | 0.002 |
|  |  | **Unc13b** | Unc-13 homolog B (C. Elegans) | NM_001042579 | 0.33 | 0.017 |
|  |  | **Olr428** | Olfactory receptor 428 | NM_001000394 | 0.34 | 0.010 |
|  |  | **Il4** | Interleukin 4 | NM_201270 | 0.34 | 0.002 |
|  |  | **Esr1** | Estrogen receptor 1 | NM_012689 | 0.34 | 0.002 |
|  |  | **Plek2** | Pleckstrin 2 | NM_001114180 | 0.34 | 0.006 |
|  |  | **Sstr1** | Somatostatin receptor 1 | NM_012719 | 0.35 | 0.010 |
|  |  | **Fgf8** | Fibroblast growth factor 8 | NM_133286 | 0.35 | 0.004 |
|  |  | **Olr1378** | Olfactory receptor 1378 | NM_214828 | 0.36 | 0.044 |
|  |  | **Afap1l2** | Actin filament associated protein 1-like 2 | XM_001064140 | 0.36 | 0.002 |
|  |  | **Prkcz** | Protein kinase C, zeta | NM_022507 | 0.37 | 0.012 |
|  |  | **Rasal3** | RAS protein activator like 3 | NM_001134562 | 0.37 | 0.028 |
|  |  | **Htr6** | 5-hydroxytryptamine (serotonin) receptor 6 | NM_024365 | 0.39 | 0.046 |
|  |  | **Rasgrp2** | RAS guanyl releasing protein 2 (calcium and DAG-regulated) | NM_001082977 | 0.39 | 0.010 |
|  |  | **Adcyap1** | Adenylate cyclase activating polypeptide 1 | NM_016989 | 0.40 | 0.002 |
|  |  | **Casp8** | Caspase 8 | NM_022277 | 0.40 | 0.028 |
|  |  | **Gna15** | Guanine nucleotide binding protein, alpha 15 | NM_053542 | 0.40 | 0.001 |
|  |  | **Oprl1** | Opiate receptor-like 1 | NM_031569 | 0.40 | 0.028 |
|  |  | **Dclk3** | Doublecortin-like kinase 3 | NM_001191800 | 0.41 | 0.000 |
|  |  | **Lck** | Lymphocyte-specific protein tyrosine kinase | NM_001100709 | 0.41 | 0.006 |
|  |  | **Olr1331** | Olfactory receptor 1331 | NM_001000790 | 0.41 | 0.040 |
|  |  | **Mas1** | MAS1 oncogene | NM_012757 | 0.42 | 0.030 |
|  |  | **Calcr** | Calcitonin receptor | NM_053816 | 0.42 | 0.040 |
|  |  | **Grem2** | Gremlin 2, cysteine knot superfamily, homolog (Xenopus laevis) | NM_001105974 | 0.42 | 0.028 |
|  |  | **Fgf3** | Fibroblast growth factor 3 | NM_130817 | 0.43 | 0.015 |
|  |  | **Wnt3** | Wingless-type MMTV integration site family, member 3 | NM_001105715 | 0.43 | 0.003 |
|  |  | **Rerg** | RAS-like, estrogen-regulated, growth-inhibitor | TC629838 | 0.43 | 0.017 |
|  |  | **Htr2c** | 5-hydroxytryptamine (serotonin) receptor 2C | NM_012765 | 0.44 | 0.041 |
|  |  | **Slc2a8** | Solute carrier family 2, (facilitated glucose transporter) member 8 | NM_053494 | 0.44 | 0.005 |
|  |  | **Adra2a** | Adrenergic, alpha-2A-, receptor | NM_012739 | 0.44 | 0.040 |
|  |  | **Socs7** | Suppressor of cytokine signaling 7 | XM_213443 | 0.45 | 0.004 |
|  |  | **Nppa** | Natriuretic peptide precursor A | NM_012612 | 0.45 | 0.014 |
|  |  | **Chn1** | Chimerin (chimaerin) 1 | NM_032083 | 0.45 | 0.010 |
|  |  | **Snca** | Synuclein, alpha (non A4 component of amyloid precursor) | S73008 | 0.45 | 0.030 |
|  |  | **Gucy2g** | Guanylate cyclase 2G | NM_139042 | 0.45 | 0.001 |
|  |  | **Adcy3** | Adenylate cyclase 3 | NM_130779 | 0.45 | 0.014 |
|  |  | **Olr1217** | Olfactory receptor 1217 | NM_001000439 | 0.46 | 0.047 |
|  |  | **Bmx** | BMX non-receptor tyrosine kinase | NM_001109016 | 0.46 | 0.020 |
|  |  | **Lingo1** | Leucine rich repeat and Ig domain containing 1 | NM_001100722 | 0.46 | 0.031 |
|  |  | **Rab40b** | Rab40b, member RAS oncogene family | NM_001107076 | 0.46 | 0.023 |
|  |  | **Madd** | MAP-kinase activating death domain | NM_053585 | 0.46 | 0.019 |
|  |  | **Sgef** | Src homology 3 domain-containing guanine nucleotide exchange factor | ENSRNOT00000019553 | 0.47 | 0.049 |
|  |  | **Olr750** | Olfactory receptor 750 | NM_001000366 | 0.47 | 0.001 |
|  |  | **Mc4r** | Melanocortin 4 receptor | NM_013099 | 0.47 | 0.033 |
|  |  | **Olr40** | Olfactory receptor 40 | NM_001000127 | 0.47 | 0.039 |
|  |  | **Rtn4rl1** | Reticulon 4 receptor-like 1 | NM_181377 | 0.47 | 0.020 |
|  |  | **Plekhg5** | Pleckstrin homology domain containing, family G (with rhogef domain) member 5 | NM_201272 | 0.48 | 0.003 |
|  |  | **Prlr** | Prolactin receptor | NM_012630 | 0.48 | 0.013 |
|  |  | **Gpr64** | G protein-coupled receptor 64 | NM_181366 | 0.48 | 0.000 |
|  |  | **Pick1** | Protein interacting with PRKCA 1 | NM_053460 | 0.48 | 0.018 |
|  |  | **Mapk8ip2** | Mitogen-activated protein kinase 8 interacting protein 2 | ENSRNOT00000055792 | 0.48 | 0.017 |
|  |  | **Prlhr** | Prolactin releasing hormone receptor | NM_139193 | 0.48 | 0.002 |
|  |  | **Olr777** | Olfactory receptor 777 | NM_001000579 | 0.49 | 0.017 |
|  |  | **Tac1** | Tachykinin 1 | NM_012666 | 0.49 | 0.010 |
|  |  | **Olr1697** | Olfactory receptor 1697 | NM_001001111 | 0.49 | 0.024 |
|  |  | **Asb2** | Ankyrin repeat and SOCS box-containing 2 | NM_001011984 | 0.49 | 0.005 |
|  |  | **Itgb2** | Integrin, beta 2 | NM_001037780 | 0.49 | 0.035 |
|  |  | **Tnfrsf11a** | Tumor necrosis factor receptor superfamily, member 11a | XM_001063501 | 0.49 | 0.005 |
|  |  | **Insig2** | Insulin induced gene 2 | NM_178091 | 0.49 | 0.013 |
|  |  | **Afp** | Alpha-fetoprotein | NM_012493 | 0.50 | 0.039 |
|  |  | **Mstn** | Myostatin | NM_019151 | 0.50 | 0.021 |
|  | | | | | | |
|  | | | | | | |
| **Synapse** | **Up-regulated** | **Gene Symbol** | **Gene name** | **Accession Number** | **Fold Change 7h** | ***p*-value** |
|  |  | **Unc13c** | Unc-13 homolog C (C. Elegans) | NM_173146 | 2.51 | 0.039 |
|  |  | **Fmr1** | Fragile X mental retardation 1 | NM_052804 | 2.34 | 0.027 |
|  |  | **Glra2** | Glycine receptor, alpha 2 | NM_012568 | 2.24 | 0.028 |
|  |  | | | | | |
|  | **Down-regulated** | **Gene Symbol** | **Gene name** | **Accession Number** | **Fold Change 7h** | ***p*-value** |
|  |  | **Unc13b** | Unc-13 homolog B (C. Elegans) | NM_001042579 | 0.33 | 0.017 |
|  |  | **Chrng** | Cholinergic receptor, nicotinic, gamma | NM_019145 | 0.38 | 0.035 |
|  |  | **Rasgrp2** | RAS guanyl releasing protein 2 (calcium and DAG-regulated) | NM_001082977 | 0.39 | 0.010 |
|  |  | **Snap25** | Synaptosomal-associated protein 25 | NM_030991 | 0.43 | 0.027 |
|  |  | **Chrna4** | Cholinergic receptor, nicotinic, alpha 4 | NM_024354 | 0.43 | 0.025 |
|  |  | **Ache** | Acetylcholinesterase | NM_172009 | 0.44 | 0.015 |
|  |  | **Slc2a8** | Solute carrier family 2, (facilitated glucose transporter) member 8 | NM_053494 | 0.44 | 0.005 |
|  |  | **Svop** | SV2 related protein | NM_134404 | 0.44 | 0.033 |
|  |  | **Adra2a** | Adrenergic, alpha-2A-, receptor | NM_012739 | 0.44 | 0.040 |
|  |  | **Slc17a7** | Solute carrier family 17 (sodium-dependent inorganic phosphate cotransporter), member 7 | NM_053859 | 0.45 | 0.022 |
|  |  | **Sema4f** | Sema domain, immunoglobulin domain (Ig), transmembrane domain (TM) and short cytoplasmic domain, (semaphorin) 4F | NM_019272 | 0.45 | 0.031 |
|  |  | **Snca** | Synuclein, alpha (non A4 component of amyloid precursor) | S73008 | 0.45 | 0.030 |
|  |  | **Slc17a6** | Solute carrier family 17 (sodium-dependent inorganic phosphate cotransporter), member 6 | NM_053427 | 0.46 | 0.047 |
|  |  | **Apbb1** | Amyloid beta (A4) precursor protein-binding, family B, member 1 (Fe65) | NM_080478 | 0.47 | 0.017 |
|  |  | **Syt3** | Synaptotagmin III | NM_019122 | 0.48 | 0.014 |
|  |  | **Pick1** | Protein interacting with PRKCA 1 | NM_053460 | 0.48 | 0.018 |
|  |  | **Synpr** | Synaptoporin | NM_023974 | 0.48 | 0.025 |
|  |  | **Syt17** | Synaptotagmin XVII | NM_138849 | 0.49 | 0.013 |
|  | | | | | | |
| **Transcription** |  | **Gene Symbol** | **Gene name** | **Accession Number** | **Fold Change 7h** | ***p*-value** |
|  |  | **Pax3** | Paired box 3 | NM_053710 | 10.65 | 0.044 |
|  |  | **Mkx** | Mohawk homeobox | ENSRNOT00000025623 | 9.17 | 0.040 |
|  |  | **Tcf7l2** | Transcription factor 7-like 2 (T-cell specific, HMG-box) | NM_001191052 | 7.39 | 0.050 |
|  |  | **Il6** | Interleukin 6 | NM_012589 | 7.19 | 0.022 |
|  | **Up-regulated** | **Ereg** | Epiregulin | NM_021689 | 6.11 | 0.002 |
|  |  | **Fst** | Follistatin | NM_012561 | 4.89 | 0.020 |
|  |  | **Nr4a3** | Nuclear receptor subfamily 4, group A, member 3 | NM_017352 | 4.54 | 0.003 |
|  |  | **Bmp2** | Bone morphogenetic protein 2 | NM_017178 | 4.00 | 0.003 |
|  |  | **Runx1** | Runt-related transcription factor 1 | NM_017325 | 3.90 | 0.000 |
|  |  | **Tlr2** | Toll-like receptor 2 | NM_198769 | 3.58 | 0.005 |
|  |  | **Batf3** | Basic leucine zipper transcription factor, ATF-like 3 | NM_021865 | 2.95 | 0.005 |
|  |  | **Uncx** | UNC homeobox | NM_017179 | 2.90 | 0.001 |
|  |  | **Foxe3** | Forkhead box E3 | ENSRNOT00000010208 | 2.85 | 0.017 |
|  |  | **Hlx** | H2.0-like homeobox | NM_001077674 | 2.83 | 0.000 |
|  |  | **Hmga1** | High mobility group AT-hook 1 | NM_139327 | 2.82 | 0.011 |
|  |  | **Pax1** | Paired box 1 | NM_001107787 | 2.59 | 0.030 |
|  |  | **Wnt6** | Wingless-type MMTV integration site family, member 6 | NM_001108226 | 2.39 | 0.048 |
|  |  | **Myc** | Myelocytomatosis oncogene | NM_012603 | 2.38 | 0.014 |
|  |  | **Sox7** | SRY (sex determining region Y)-box 7 | NM_001106045 | 2.37 | 0.044 |
|  |  | **Eid3** | EP300 interacting inhibitor of differentiation 3 | NM_001044304 | 2.37 | 0.027 |
|  |  | **Npm3** | Nucleophosmin/nucleoplasmin, 3 | ENSRNOT00000023963 | 2.29 | 0.012 |
|  |  | **Tgfb1** | Transforming growth factor, beta 1 | NM_021578 | 2.27 | 0.047 |
|  |  | **Junb** | Jun B proto-oncogene | NM_021836 | 2.26 | 0.001 |
|  |  | **Rgd1561672** | Similar to novel protein | XM_001065540 | 2.24 | 0.014 |
|  |  | **Klf5** | Kruppel-like factor 5 | NM_053394 | 2.23 | 0.012 |
|  |  | **Atf3** | Activating transcription factor 3 | NM_012912 | 2.20 | 0.021 |
|  |  | **Fzd6** | Frizzled homolog 6 (Drosophila) | NM_001130536 | 2.15 | 0.005 |
|  |  | **Zfp217** | Zinc finger protein 217 | NM_001107813 | 2.14 | 0.003 |
|  |  | **Sin3a** | SIN3 homolog A, transcription regulator (yeast) | NM_001108761 | 2.03 | 0.004 |
|  |  | **Hmgb1** | High mobility group box 1 | NM_012963 | 2.00 | 0.009 |
|  |  | | | | | |
|  | **Down-regulated** | **Gene Symbol** | **Gene name** | **Accession Number** | **Fold Change 7h** | ***p*-value** |
|  |  | **Hes5** | Hairy and enhancer of split 5 (Drosophila) | NM_024383 | 0.18 | 0.033 |
|  |  | **Nostrin** | Nitric oxide synthase trafficker | NM_001024260 | 0.24 | 0.029 |
|  |  | **Cdkn1c** | Cyclin-dependent kinase inhibitor 1C | NM_182735 | 0.25 | 0.005 |
|  |  | **Tp63** | Tumor protein p63 | NM_019221 | 0.25 | 0.000 |
|  |  | **Pou4f3** | POU class 4 homeobox 3 | NM_001108889 | 0.26 | 0.013 |
|  |  | **Tbx1** | T-box 1 | NM_001108322 | 0.33 | 0.037 |
|  |  | **Il4** | Interleukin 4 | NM_201270 | 0.34 | 0.002 |
|  |  | **Esr1** | Estrogen receptor 1 | NM_012689 | 0.34 | 0.002 |
|  |  | **Rgd1304879** | Similar to Zinc finger protein 398 (Zinc finger DNA binding protein p52/p71) | NM_001014056 | 0.35 | 0.004 |
|  |  | **Afap1l2** | Actin filament associated protein 1-like 2 | XM_001064140 | 0.36 | 0.002 |
|  |  | **Hoxc6** | Homeo box C6 | XM_001069410 | 0.40 | 0.015 |
|  |  | **Dmrtc1a** | DMRT-like family c1a | NM_001025288 | 0.40 | 0.008 |
|  |  | **Plagl1** | Pleiomorphic adenoma gene-like 1 | NM_012760 | 0.41 | 0.015 |
|  |  | **Hcfc1** | Host cell factor C1 | NM_001139507 | 0.43 | 0.005 |
|  |  | **Foxl2** | Forkhead box L2 | ENSRNOT00000023091 | 0.44 | 0.004 |
|  |  | **Egr4** | Early growth response 4 | NM_019137 | 0.44 | 0.008 |
|  |  | **Loc680273** | Similar to Forkhead box protein L1 (Forkhead-related protein FKHL11) (Forkhead-related transcription factor 7) (FREAC-7) | XM_001056413 | 0.45 | 0.036 |
|  |  | **Tceanc** | Transcription elongation factor A (SII) N-terminal and central domain containing | NM_001109015 | 0.46 | 0.007 |
|  |  | **Zfp112** | Zinc finger protein 112 | NM_001107487 | 0.47 | 0.005 |
|  |  | **Apbb1** | Amyloid beta (A4) precursor protein-binding, family B, member 1 (Fe65) | NM_080478 | 0.47 | 0.017 |
|  |  | **Zfp324** | Zinc finger protein 324 | ENSRNOT00000036874 | 0.48 | 0.009 |
|  |  | **Zcchc12** | Zinc finger, CCHC domain containing 12 | NM_001014065 | 0.48 | 0.021 |
|  |  | **Itgb2** | Integrin, beta 2 | NM_001037780 | 0.49 | 0.035 |
|  |  | **Tnfrsf11a** | Tumor necrosis factor receptor superfamily, member 11a | XM_001063501 | 0.49 | 0.005 |
|  |  | **Zbtb24** | Zinc finger and BTB domain containing 24 | NM_001098667 | 0.50 | 0.013 |
|  |  | **Mstn** | Myostatin | NM_019151 | 0.50 | 0.021 |
|  | | | | | | |
| **Receptor Activity** | **Up-regulated** | **Gene Symbol** | **Gene name** | **Accession Number** | **Fold Change 7h** | ***p*-value** |
|  |  | **Thbd** | Thrombomodulin | NM_031771 | 7.03 | 0.012 |
|  |  | **Nr4a3** | Nuclear receptor subfamily 4, group A, member 3 | NM_017352 | 4.54 | 0.003 |
|  |  | **Vom2r75** | Vomeronasal 2 receptor, 75 | NM_173320 | 3.83 | 0.028 |
|  |  | **Itgb6** | Integrin, beta 6 | NM_001004263 | 3.75 | 0.003 |
|  |  | **Olr374** | Olfactory receptor 374 | NM_001001289 | 3.65 | 0.011 |
|  |  | **Tlr2** | Toll-like receptor 2 | NM_198769 | 3.58 | 0.005 |
|  |  | **Plaur** | Plasminogen activator, urokinase receptor | NM_134352 | 3.13 | 0.006 |
|  |  | **F2rl1** | Coagulation factor II (thrombin) receptor-like 1 | NM_053897 | 2.46 | 0.007 |
|  |  | **Extl3** | Exostoses (multiple)-like 3 | NM_020097 | 2.38 | 0.029 |
|  |  | **Olr1694** | Olfactory receptor 1694 | NM_001001110 | 2.37 | 0.001 |
|  |  | **Pvr** | Poliovirus receptor | NM_017076 | 2.33 | 0.021 |
|  |  | **Olr1673** | Olfactory receptor 1673 | NM_001000268 | 2.29 | 0.014 |
|  |  | **Cd44** | Cd44 molecule | NM_012924 | 2.28 | 0.041 |
|  |  | **Glra2** | Glycine receptor, alpha 2 | NM_012568 | 2.24 | 0.028 |
|  |  | **Rxfp3** | Relaxin/insulin-like family peptide receptor 3 | NM_001008310 | 2.15 | 0.018 |
|  |  | **Fzd6** | Frizzled homolog 6 (Drosophila) | NM_001130536 | 2.15 | 0.005 |
|  |  | **Olr803** | Olfactory receptor 803 | NM_001000853 | 2.08 | 0.009 |
|  |  | **Cx3cr1** | Chemokine (C-X3-C motif) receptor 1 | NM_133534 | 2.06 | 0.041 |
|  |  | **Vom2r56** | Vomeronasal 2 receptor, 56 | NM_001099484 | 2.02 | 0.049 |
|  |  | | | | | |
|  | **Down-regulated** | **Gene Symbol** | **Gene name** | **Accession Number** | **Fold Change 7h** | ***p*-value** |
|  |  | **Olr282** | Olfactory receptor 282 | NM_001000224 | 0.19 | 0.038 |
|  |  | **Agtr1b** | Angiotensin II receptor, type 1b | NM_031009 | 0.21 | 0.002 |
|  |  | **Ptafr** | Platelet-activating factor receptor | NM_053321 | 0.30 | 0.025 |
|  |  | **Klrb1a** | Killer cell lectin-like receptor subfamily B, member 1A | NM_001010964 | 0.31 | 0.001 |
|  |  | **Gpr6** | G protein-coupled receptor 6 | NM_031806 | 0.31 | 0.003 |
|  |  | **Htr4** | 5-hydroxytryptamine (serotonin) receptor 4 | NM_012853 | 0.32 | 0.002 |
|  |  | **Sstr3** | Somatostatin receptor 3 | NM_133522 | 0.32 | 0.003 |
|  |  | **Cd244** | Cd244 molecule, natural killer cell receptor 2B4 | NM_022259 | 0.33 | 0.007 |
|  |  | **Gpr160** | G protein-coupled receptor 160 | NM_001025147 | 0.34 | 0.008 |
|  |  | **Olr428** | Olfactory receptor 428 | NM_001000394 | 0.34 | 0.010 |
|  |  | **Gfra4** | GDNF family receptor alpha 4 | NM_023967 | 0.34 | 0.010 |
|  |  | **Trpc7** | Transient receptor potential cation channel, subfamily C, member 7 | NM_001191691 | 0.34 | 0.031 |
|  |  | **Esr1** | Estrogen receptor 1 | NM_012689 | 0.34 | 0.002 |
|  |  | **Sstr1** | Somatostatin receptor 1 | NM_012719 | 0.35 | 0.010 |
|  |  | **Sorcs3** | Sortilin-related VPS10 domain containing receptor 3 | NM_001106367 | 0.35 | 0.013 |
|  |  | **Olr1378** | Olfactory receptor 1378 | NM_214828 | 0.36 | 0.044 |
|  |  | **Trpc6** | Transient receptor potential cation channel, subfamily C, member 6 | NM_053559 | 0.37 | 0.009 |
|  |  | **Gpr68** | G protein-coupled receptor 68 | NM_001108049 | 0.38 | 0.000 |
|  |  | **Il8ra** | Interleukin 8 receptor, alpha | NM_019310 | 0.38 | 0.015 |
|  |  | **Chrng** | Cholinergic receptor, nicotinic, gamma | NM_019145 | 0.38 | 0.035 |
|  |  | **Htr6** | 5-hydroxytryptamine (serotonin) receptor 6 | NM_024365 | 0.39 | 0.046 |
|  |  | **Pth2r** | Parathyroid hormone 2 receptor | NM_031089 | 0.40 | 0.038 |
|  |  | **Oprl1** | Opiate receptor-like 1 | NM_031569 | 0.40 | 0.028 |
|  |  | **Olr1331** | Olfactory receptor 1331 | NM_001000790 | 0.41 | 0.040 |
|  |  | **Mas1** | MAS1 oncogene | NM_012757 | 0.42 | 0.030 |
|  |  | **Calcr** | Calcitonin receptor | NM_053816 | 0.42 | 0.040 |
|  |  | **Rtn4r** | Reticulon 4 receptor | NM_053613 | 0.43 | 0.006 |
|  |  | **Chrna4** | Cholinergic receptor, nicotinic, alpha 4 | NM_024354 | 0.43 | 0.025 |
|  |  | **Htr2c** | 5-hydroxytryptamine (serotonin) receptor 2C | NM_012765 | 0.44 | 0.041 |
|  |  | **Tnfrsf17** | Tumor necrosis factor receptor superfamily, member 17 | NM_001105761 | 0.44 | 0.026 |
|  |  | **Adra2a** | Adrenergic, alpha-2A-, receptor | NM_012739 | 0.44 | 0.040 |
|  |  | **Lpar4** | Lysophosphatidic acid receptor 4 | NM_001106940 | 0.45 | 0.021 |
|  |  | **Chn1** | Chimerin (chimaerin) 1 | NM_032083 | 0.45 | 0.010 |
|  |  | **Sema4f** | Sema domain, immunoglobulin domain (Ig), transmembrane domain (TM) and short cytoplasmic domain, (semaphorin) 4F | NM_019272 | 0.45 | 0.031 |
|  |  | **Gucy2g** | Guanylate cyclase 2G | NM_139042 | 0.45 | 0.001 |
|  |  | **Olr1217** | Olfactory receptor 1217 | NM_001000439 | 0.46 | 0.047 |
|  |  | **Prom2** | Prominin 2 | NM_138857 | 0.46 | 0.031 |
|  |  | **Olr750** | Olfactory receptor 750 | NM_001000366 | 0.47 | 0.001 |
|  |  | **Mc4r** | Melanocortin 4 receptor | NM_013099 | 0.47 | 0.033 |
|  |  | **Olr40** | Olfactory receptor 40 | NM_001000127 | 0.47 | 0.039 |
|  |  | **Rtn4rl1** | Reticulon 4 receptor-like 1 | NM_181377 | 0.47 | 0.020 |
|  |  | **Prlr** | Prolactin receptor | NM_012630 | 0.48 | 0.013 |
|  |  | **Gpr176** | G protein-coupled receptor 176 | ENSRNOT00000007882 | 0.48 | 0.014 |
|  |  | **Gpr64** | G protein-coupled receptor 64 | NM_181366 | 0.48 | 0.000 |
|  |  | **Prlhr** | Prolactin releasing hormone receptor | NM_139193 | 0.48 | 0.002 |
|  |  | **Olr777** | Olfactory receptor 777 | NM_001000579 | 0.49 | 0.017 |
|  |  | **Olr1697** | Olfactory receptor 1697 | NM_001001111 | 0.49 | 0.024 |
|  |  | **Itgb2** | Integrin, beta 2 | NM_001037780 | 0.49 | 0.035 |
|  |  | **Trpm2** | Transient receptor potential cation channel, subfamily M, member 2 | NM_001011559 | 0.49 | 0.002 |
|  |  | **Tnfrsf11a** | Tumor necrosis factor receptor superfamily, member 11a | XM_001063501 | 0.49 | 0.005 |
|  | | | | | | |
| **Glutamate Secretion** | **Down-regulated** | **Gene Symbol** | **Gene name** | **Accession Number** | **Fold Change 7h** | ***p*-value** |
|  |  | **Htr6** | 5-hydroxytryptamine (serotonin) receptor 6 | NM_024365 | 0.39 | 0.046 |
|  |  | **Snca** | Synuclein, alpha (non A4 component of amyloid precursor) | S73008 | 0.45 | 0.030 |
|  | | | | | | |
| **RNA Biosynthetic Process** | **Up-regulation** | **Gene Symbol** | **Gene name** | **Accession Number** | **Fold Change 7h** | ***p*-value** |
|  |  | **Pax3** | Paired box 3 | NM_053710 | 10.65 | 0.044 |
|  |  | **Mkx** | Mohawk homeobox | ENSRNOT00000025623 | 9.17 | 0.040 |
|  |  | **Tcf7l2** | Transcription factor 7-like 2 (T-cell specific, HMG-box) | NM_001191052 | 7.39 | 0.050 |
|  |  | **Il6** | Interleukin 6 | NM_012589 | 7.19 | 0.022 |
|  |  | **Ereg** | Epiregulin | NM_021689 | 6.11 | 0.002 |
|  |  | **Fst** | Follistatin | NM_012561 | 4.89 | 0.020 |
|  |  | **Nr4a3** | Nuclear receptor subfamily 4, group A, member 3 | NM_017352 | 4.54 | 0.003 |
|  |  | **Bmp2** | Bone morphogenetic protein 2 | NM_017178 | 4.00 | 0.003 |
|  |  | **Runx1** | Runt-related transcription factor 1 | NM_017325 | 3.90 | 0.000 |
|  |  | **Tlr2** | Toll-like receptor 2 | NM_198769 | 3.58 | 0.005 |
|  |  | **Batf3** | Basic leucine zipper transcription factor, ATF-like 3 | NM_021865 | 2.95 | 0.005 |
|  |  | **Uncx** | UNC homeobox | NM_017179 | 2.90 | 0.001 |
|  |  | **Foxe3** | Forkhead box E3 | ENSRNOT00000010208 | 2.85 | 0.017 |
|  |  | **Hlx** | H2.0-like homeobox | NM_001077674 | 2.83 | 0.000 |
|  |  | **Hmga1** | High mobility group AT-hook 1 | NM_139327 | 2.82 | 0.011 |
|  |  | **Pax1** | Paired box 1 | NM_001107787 | 2.59 | 0.030 |
|  |  | **Wnt6** | Wingless-type MMTV integration site family, member 6 | NM_001108226 | 2.39 | 0.048 |
|  |  | **Myc** | Myelocytomatosis oncogene | NM_012603 | 2.38 | 0.014 |
|  |  | **Sox7** | SRY (sex determining region Y)-box 7 | NM_001106045 | 2.37 | 0.044 |
|  |  | **Npm3** | Nucleophosmin/nucleoplasmin, 3 | ENSRNOT00000023963 | 2.29 | 0.012 |
|  |  | **Tgfb1** | Transforming growth factor, beta 1 | NM_021578 | 2.27 | 0.047 |
|  |  | **Junb** | Jun B proto-oncogene | NM_021836 | 2.26 | 0.001 |
|  |  | **Rgd1561672** | Similar to novel protein | XM_001065540 | 2.24 | 0.014 |
|  |  | **Klf5** | Kruppel-like factor 5 | NM_053394 | 2.23 | 0.012 |
|  |  | **Atf3** | Activating transcription factor 3 | NM_012912 | 2.20 | 0.021 |
|  |  | **Fzd6** | Frizzled homolog 6 (Drosophila) | NM_001130536 | 2.15 | 0.005 |
|  |  | **Sin3a** | SIN3 homolog A, transcription regulator (yeast) | NM_001108761 | 2.03 | 0.004 |
|  |  | **Hmgb1** | High mobility group box 1 | NM_012963 | 2.00 | 0.009 |
|  |  | | | | | |
|  | **Down-regulation** | **Gene Symbol** | **Gene name** | **Accession Number** | **Fold Change 7h** | ***p*-value** |
|  |  | **Hes5** | Hairy and enhancer of split 5 (Drosophila) | NM_024383 | 0.18 | 0.033 |
|  |  | **Nostrin** | Nitric oxide synthase trafficker | NM_001024260 | 0.24 | 0.029 |
|  |  | **Cdkn1c** | Cyclin-dependent kinase inhibitor 1C | NM_182735 | 0.25 | 0.005 |
|  |  | **Tp63** | Tumor protein p63 | NM_019221 | 0.25 | 0.000 |
|  |  | **Pou4f3** | POU class 4 homeobox 3 | NM_001108889 | 0.26 | 0.013 |
|  |  | **Tbx1** | T-box 1 | NM_001108322 | 0.33 | 0.037 |
|  |  | **Il4** | Interleukin 4 | NM_201270 | 0.34 | 0.002 |
|  |  | **Esr1** | Estrogen receptor 1 | NM_012689 | 0.34 | 0.002 |
|  |  | **Rgd1304879** | Similar to Zinc finger protein 398 (Zinc finger DNA binding protein p52/p71) | NM_001014056 | 0.35 | 0.004 |
|  |  | **Afap1l2** | Actin filament associated protein 1-like 2 | XM_001064140 | 0.36 | 0.002 |
|  |  | **Hoxc6** | Homeo box C6 | XM_001069410 | 0.40 | 0.015 |
|  |  | **Plagl1** | Pleiomorphic adenoma gene-like 1 | NM_012760 | 0.41 | 0.015 |
|  |  | **Foxl2** | Forkhead box L2 | ENSRNOT00000023091 | 0.44 | 0.004 |
|  |  | **Loc680273** | Similar to Forkhead box protein L1 (Forkhead-related protein FKHL11) (Forkhead-related transcription factor 7) (FREAC-7) | XM_001056413 | 0.45 | 0.036 |
|  |  | **Tceanc** | Transcription elongation factor A (SII) N-terminal and central domain containing | NM_001109015 | 0.46 | 0.007 |
|  |  | **Zfp112** | Zinc finger protein 112 | NM_001107487 | 0.47 | 0.005 |
|  |  | **Apbb1** | Amyloid beta (A4) precursor protein-binding, family B, member 1 (Fe65) | NM_080478 | 0.47 | 0.017 |
|  |  | **Zfp324** | Zinc finger protein 324 | ENSRNOT00000036874 | 0.48 | 0.009 |
|  |  | **Itgb2** | Integrin, beta 2 | NM_001037780 | 0.49 | 0.035 |
|  |  | **Tnfrsf11a** | Tumor necrosis factor receptor superfamily, member 11a | XM_001063501 | 0.49 | 0.005 |
|  |  | **Zbtb24** | Zinc finger and BTB domain containing 24 | NM_001098667 | 0.50 | 0.013 |
|  | | | | | | |
| **Response to Oxidative Stress** | **Up-regulation** | **Gene Symbol** | **Gene name** | **Accession Number** | **Fold Change 7h** | ***p*-value** |
|  |  | **Areg** | Amphiregulin | NM_017123 | 3.07 | 0.017 |
|  |  | **Sin3a** | SIN3 homolog A, transcription regulator (yeast) | NM_001108761 | 2.03 | 0.004 |
|  |  | | | | | |
|  | **Down-regulation** | **Gene Symbol** | **Gene name** | **Accession Number** | **Fold Change 7h** | ***p*-value** |
|  |  | **Mmp9** | Matrix metallopeptidase 9 | NM_031055 | 0.23 | 0.027 |
|  |  | **Trpc7** | Transient receptor potential cation channel, subfamily C, member 7 | NM_001191691 | 0.34 | 0.031 |
|  |  | **Fgf8** | Fibroblast growth factor 8 | NM_133286 | 0.35 | 0.004 |
|  |  | **Lck** | Lymphocyte-specific protein tyrosine kinase | NM_001100709 | 0.41 | 0.006 |
|  |  | **Nox1** | NADPH oxidase 1 | NM_053683 | 0.43 | 0.002 |
|  |  | **Chrna4** | Cholinergic receptor, nicotinic, alpha 4 | NM_024354 | 0.43 | 0.025 |
|  |  | **Snca** | Synuclein, alpha (non A4 component of amyloid precursor) | S73008 | 0.45 | 0.030 |
|  |  | **Trpm2** | Transient receptor potential cation channel, subfamily M, member 2 | NM_001011559 | 0.49 | 0.002 |
