# Supplementary material for: In Vitro Ischemia Triggers a Transcriptional Response to Down-Regulate Synaptic Proteins in Hippocampal Neurons
Source: PLoS One. 2014 Jun 24;9(6):e99958. doi: 10.1371/journal.pone.0099958 (PMC4069008; doi:10.1371/journal.pone.0099958)
Supplement: Table S6 — List of genes up-regulated and down-regulated at 24 h after OGD for different ontological classes. Gene ontology analyses included genes that had a p-value <0.05 and a fold change of 2.0 and were performed using GoMiner. Classes were selected manually. Note that some genes are included in more than one class. (DOCX) [file pone.0099958.s007.docx]

| **Apoptosis** | **Up-regulated** | **Gene Symbol** | **Gene name** | **Accession Number** | **Fold Change 24h** | ***p*-value** |
| --- | --- | --- | --- | --- | --- | --- |
|  |  | **Ctgf** | Connective tissue growth factor | NM_022266 | 9.84 | 0.012 |
|  |  | **Angptl4** | Angiopoietin-like 4 | NM_199115 | 4.91 | 0.008 |
|  |  | **Anxa1** | Annexin A1 | NM_012904 | 4.62 | 0.018 |
|  |  | **Casp1** | Caspase 1 | NM_012762 | 4.56 | 0.026 |
|  |  | **Mycs** | Myc-like oncogene, s-myc protein | NM_021837 | 4.25 | 0.042 |
|  |  | **Cd44** | Cd44 molecule | NM_012924 | 3.07 | 0.032 |
|  |  | **Nqo1** | NAD(P)H dehydrogenase, quinone 1 | NM_017000 | 2.76 | 0.037 |
|  |  | **Ccr5** | Chemokine (C-C motif) receptor 5 | NM_053960 | 2.75 | 0.006 |
|  |  | **Hoxa13** | Homeo box A13 | XM_575481 | 2.72 | 0.047 |
|  |  | **Pdgfrb** | Platelet derived growth factor receptor, beta polypeptide | NM_031525 | 2.54 | 0.04 |
|  |  | **Six1** | SIX homeobox 1 | NM_053759 | 2.48 | 0.025 |
|  |  | **B4galt1** | UDP-Gal:betaglcnac beta 1,4- galactosyltransferase, polypeptide 1 | NM_053287 | 2.35 | 0.009 |
|  |  | **Cdkn2b** | Cyclin-dependent kinase inhibitor 2B (p15, inhibits CDK4) | NM_130812 | 2.31 | 0.034 |
|  |  | **Birc3** | Baculoviral IAP repeat-containing 3 | NM_023987 | 2.3 | 0.012 |
|  |  | **Gata6** | GATA binding protein 6 | NM_019185 | 2.27 | 0.035 |
|  |  | **Prkcd** | Protein kinase C, delta | NM_133307 | 2.2 | 0.013 |
|  |  | **Id1** | Inhibitor of DNA binding 1 | NM_012797 | 2.17 | 0.032 |
|  |  | **Pparg** | Peroxisome proliferator-activated receptor gamma | NM_001145367 | 2.17 | 0.024 |
|  |  | **Mitf** | Microphthalmia-associated transcription factor | NM_001191089 | 2.12 | 0.019 |
|  |  | **Casp14** | Caspase 14 | NM_001191776 | 2.06 | 0.019 |
|  |  | **Hand2** | Heart and neural crest derivatives expressed 2 | NM_022696 | 2.03 | 0.038 |
|  |  | **F3** | Coagulation factor III (thromboplastin, tissue factor) | NM_013057 | 2,00 | 0.04 |
|  |  | | | | | |
|  | **Down-regulated** | **Gene Symbol** | **Gene name** | **Accession Number** | **Fold Change 24h** | ***p*-value** |
|  |  | **Cd27** | CD27 molecule | NM_001024335 | 0.31 | 0.035 |
|  |  | **Grin1** | Glutamate receptor, ionotropic, N-methyl D-aspartate 1 | NM_017010 | 0.39 | 0.038 |
|  |  | **Dhcr24** | 24-dehydrocholesterol reductase | NM_001080148 | 0.43 | 0.045 |
|  |  | **Grin2a** | Glutamate receptor, ionotropic, N-methyl D-aspartate 2A | NM_012573 | 0.44 | 0.038 |
|  |  | **Ptgs2** | Prostaglandin-endoperoxide synthase 2 | NM_017232 | 0.48 | 0.008 |
|  | | | | | | |
| **Inflammatory Response** | **Up-regulated** | **Gene Symbol** | **Gene name** | **Accession Number** | **Fold Change 24h** | ***p*-value** |
|  |  | **Cxcl2** | Chemokine (C-X-C motif) ligand 2 | NM_053647 | 8.61 | 0.049 |
|  |  | **Cxcl10** | Chemokine (C-X-C motif) ligand 10 | NM_139089 | 7.57 | 0.015 |
|  |  | **Reg3g** | Regenerating islet-derived 3 gamma | NM_173097 | 6.57 | 0.005 |
|  |  | **Calca** | Calcitonin-related polypeptide alpha | NM_017338 | 5.11 | 0.032 |
|  |  | **Anxa1** | Annexin A1 | NM_012904 | 4.62 | 0.018 |
|  |  | **Il5** | Interleukin 5 | NM_021834 | 4.57 | 0.026 |
|  |  | **Olr1** | Oxidized low density lipoprotein (lectin-like) receptor 1 | NM_133306 | 4.1 | 0.016 |
|  |  | **Fgg** | Fibrinogen gamma chain | NM_012559 | 3.36 | 0.039 |
|  |  | **Ccl3** | Chemokine (C-C motif) ligand 3 | NM_013025 | 3.27 | 0.018 |
|  |  | **Cd44** | Cd44 molecule | NM_012924 | 3.07 | 0.032 |
|  |  | **Ccr5** | Chemokine (C-C motif) receptor 5 | NM_053960 | 2.75 | 0.006 |
|  |  | **S100a9** | S100 calcium binding protein A9 | NM_053587 | 2.49 | 0.029 |
|  |  | **Il1rl1** | Interleukin 1 receptor-like 1 | NM_001127689 | 2.39 | 0.022 |
|  |  | **B4galt1** | UDP-Gal:betaglcnac beta 1,4- galactosyltransferase, polypeptide 1 | NM_053287] | 2.35 | 0.009 |
|  |  | **Pparg** | Peroxisome proliferator-activated receptor gamma | NM_001145367 | 2.17 | 0.024 |
|  |  | **F3** | Coagulation factor III (thromboplastin, tissue factor) | NM_013057 | 2,00 | 0.04 |
|  |  | | | | | |
|  | **Down-regulated** | **Gene Symbol** | **Gene name** | **Accession Number** | **Fold Change 24h** | ***p*-value** |
|  |  | **F2** | Coagulation factor II (thrombin) | NM_022924 | 0.3 | 0.009 |
|  |  | **Ptgs2** | Prostaglandin-endoperoxide synthase 2 | NM_017232 | 0.48 | 0.008 |
|  | | | | | | |
| **Ion Transmembrane Transporter Activity** | **Up-regulated** | **Gene Symbol** | **Gene name** | **Accession Number** | **Fold Change 24h** | ***p*-value** |
|  |  | **S100a6** | S100 calcium binding protein A6 | NM_053485 | 7.79 | 0.027 |
|  |  | **Clca2** | Chloride channel calcium activated 2 | NM_001013202 | 4.67 | 0.038 |
|  |  | **Ucp3** | Uncoupling protein 3 (mitochondrial, proton carrier) | NM_013167 | 4.16 | 0.012 |
|  |  | **Slc17a1** | Solute carrier family 17 (sodium phosphate), member 1 | NM_133554 | 3.4 | 0.012 |
|  |  | **Clic1** | Chloride intracellular channel 1 | NM_001002807 | 3.04 | 0.017 |
|  |  | **Clic5** | Chloride intracellular channel 5 | NM_053603 | 2.26 | 0.026 |
|  |  | **Slco2b1** | Solute carrier organic anion transporter family, member 2b1 | NM_080786 | 2.17 | 0.048 |
|  |  | **Kcna10** | Potassium voltage-gated channel, shaker-related subfamily, member 10 | NM_001191713 | 2.1 | 0.026 |
|  |  | **Atp1b4** | Atpase, (Na+)/K+ transporting, beta 4 polypeptide | NM_053381 | 2.07 | 0.026 |
|  |  | **Slc12a7** | Solute carrier family 12 (potassium/chloride transporters), member 7 | NM_001013144 | 2.07 | 0.045 |
|  |  | **Atp1a4** | Atpase, Na+/K+ transporting, alpha 4 polypeptide | NM_022848 | 2.02 | 0.018 |
|  |  | | | | | |
|  | **Down-regulated** | **Gene Symbol** | **Gene name** | **Accession Number** | **Fold Change 24h** | ***p*-value** |
|  |  | **Grin1** | Glutamate receptor, ionotropic, N-methyl D-aspartate 1 | NM_017010 | 0.39 | 0.038 |
|  |  | **Trpc5** | Transient receptor potential cation channel, subfamily C, member 5 | NM_080898 | 0.41 | 0.048 |
|  |  | **Grin2a** | Glutamate receptor, ionotropic, N-methyl D-aspartate 2A | NM_012573 | 0.44 | 0.038 |
|  |  | **Grin2b** | Glutamate receptor, ionotropic, N-methyl D-aspartate 2B | NM_012574 | 0.44 | 0.022 |
|  |  | **Kcnq3** | Potassium voltage-gated channel, KQT-like subfamily, member 3 | NM_031597 | 0.46 | 0.036 |
|  |  | **Gabra3** | Gamma-aminobutyric acid (GABA) A receptor, alpha 3 | NM_017069 | 0.47 | 0.004 |
|  |  | **Gabrb3** | Gamma-aminobutyric acid (GABA) A receptor, beta 3 | NM_017065 | 0.48 | 0.015 |
|  |  | **Rgd1562492** | Similar to Orphan sodium- and chloride-dependent neurotransmitter transporter NTT5 (Solute carrier family 6 member 16) | NM_001109146 | 0.5 | 0.038 |
|  |  | **Slc8a1** | Solute carrier family 8 (sodium/calcium exchanger), member 1 | NM_019268 | 0.5 | 0.022 |
|  | | | | | | |
| **Metabolic Process** | **Up-regulated** | **Gene Symbol** | **Gene name** | **Accession Number** | **Fold Change 24h** | ***p*-value** |
|  |  | **Eif4g2** | Eukaryotic translation initiation factor 4, gamma 2 | AB256044 | 12.23 | 0.005 |
|  |  | **Loc293989** | Connective tissue growth factor | NM_022266 | 9.84 | 0.012 |
|  |  | **Ctgf** | Connective tissue growth factor | NM_022266 | 9.84 | 0.012 |
|  |  | **Ptgr1** | Prostaglandin reductase 1 | NM_138863 | 7.03 | 0.028 |
|  |  | **Hmga2** | High mobility group AT-hook 2 | NM_032070 | 6.99 | 0.023 |
|  |  | **Cd80** | Cd80 molecule | NM_012926 | 6.69 | 0.049 |
|  |  | **Lhx1** | LIM homeobox 1 | NM_145880 | 6.02 | 0.022 |
|  |  | **Loc687736** | Similar to double homeobox, 4 | XM_001079968 | 5.48 | 0.05 |
|  |  | **Akr1b8** | Aldo-keto reductase family 1, member B8 | NM_173136 | 5.34 | 0.031 |
|  |  | **Agpat2** | 1-acylglycerol-3-phosphate O-acyltransferase 2 (lysophosphatidic acid acyltransferase, beta) | NM_001107821 | 5.26 | 0.031 |
|  |  | **Srpx2** | Sushi-repeat-containing protein, X-linked 2 | NM_001108243 | 5.22 | 0.034 |
|  |  | **Calca** | Calcitonin-related polypeptide alpha | NM_017338 | 5.11 | 0.032 |
|  |  | **Loc300308** | Similar to hypothetical protein 4930509O22 | XM_001079328 | 5.08 | 0.016 |
|  |  | **Rgd1565071** | Similar to hypothetical protein 4930509O22 | XM_001079328 | 5.08 | 0.016 |
|  |  | **Angptl4** | Angiopoietin-like 4 | NM_199115 | 4.91 | 0.008 |
|  |  | **Rgd1563833** | Similar to putative homeobox protein | ENSRNOT00000066263 | 4.82 | 0.029 |
|  |  | **Rgd1564033** | Similar to Homeobox protein OTX1 | XM_001059672 | 4.73 | 0.041 |
|  |  | **Anxa1** | Annexin A1 | NM_012904 | 4.62 | 0.018 |
|  |  | **Il5** | Interleukin 5 | NM_021834 | 4.57 | 0.026 |
|  |  | **Casp1** | Caspase 1 | NM_012762 | 4.56 | 0.026 |
|  |  | **Mycs** | Myc-like oncogene, s-myc protein | NM_021837 | 4.25 | 0.042 |
|  |  | **Pla1a** | Phospholipase A1 member A | NM_138882 | 4.24 | 0.031 |
|  |  | **Ucp3** | Uncoupling protein 3 (mitochondrial, proton carrier) | NM_013167 | 4.16 | 0.012 |
|  |  | **Olr1** | Oxidized low density lipoprotein (lectin-like) receptor 1 | NM_133306 | 4.1 | 0.016 |
|  |  | **Rgd1563334** | Similar to novel protein similar to esterases | XM_578509 | 4.1 | 0.023 |
|  |  | **Loc503327** | Similar to serine/threonine kinase | XM_578861 | 3.96 | 0.023 |
|  |  | **Akr1c18** | Aldo-keto reductase family 1, member C18 | NM_138510 | 3.91 | 0.016 |
|  |  | **Loc100365510** | Homeobox protein-like | XM_002727408 | 3.7 | 0.033 |
|  |  | **Adamts14** | ADAM metallopeptidase with thrombospondin type 1 motif, 14 | NM_001107636 | 3.68 | 0.004 |
|  |  | **Loc100363448** | Double homeobox, 1-like | XM_002728977 | 3.62 | 0.042 |
|  |  | **Map3k6** | Mitogen-activated protein kinase kinase kinase 6 | NM_001107909 | 3.48 | 0.044 |
|  |  | **Rgd1561620** | Similar to isopentenyl diphosphate delta-isomerase type 2 | XM_225507 | 3.42 | 0.034 |
|  |  | **Hoxb7** | Homeo box B7 | NM_001017480 | 3.41 | 0.038 |
|  |  | **Loc686581** | Similar to transmembrane protease, serine 11A | XM_001074837 | 3.4 | 0.017 |
|  |  | **Rbms1** | RNA binding motif, single stranded interacting protein 1 | NM_001012184 | 3.32 | 0.042 |
|  |  | **Itgb3** | Integrin, beta 3 | NM_153720 | 3.28 | 0.039 |
|  |  | **Pcolce** | Procollagen C-endopeptidase enhancer | NM_019237 | 3.25 | 0.045 |
|  |  | **Loc687536** | Similar to Forkhead box protein F1 (Forkhead-related protein FKHL5) (Forkhead-related transcription factor 1) (FREAC-1) (Hepatocyte nuclear factor 3 forkhead homolog 8) (HFH-8) | XM_001079002 | 3.17 | 0.026 |
|  |  | **Mgst2** | Microsomal glutathione S-transferase 2 | NM_001106430 | 3.14 | 0.014 |
|  |  | **Cd44** | Cd44 molecule | NM_012924 | 3.07 | 0.032 |
|  |  | **Loc291480** | Similar to ribosomal protein L19 | XM_225800 | 3.01 | 0.02 |
|  |  | **Plcb2** | Phospholipase C, beta 2 | NM_053478 | 2.94 | 0.034 |
|  |  | **Mmp3** | Matrix metallopeptidase 3 | NM_133523 | 2.94 | 0.041 |
|  |  | **Ptgs1** | Prostaglandin-endoperoxide synthase 1 | NM_017043 | 2.92 | 0.004 |
|  |  | **Icam1** | Intercellular adhesion molecule 1 | NM_012967 | 2.91 | 0.033 |
|  |  | **Nr6a1** | Nuclear receptor subfamily 6, group A, member 1 | XM_001060553 | 2.87 | 0.038 |
|  |  | **Lyve1** | Lymphatic vessel endothelial hyaluronan receptor 1 | NM_001106286 | 2.8 | 0.038 |
|  |  | **Nqo1** | NAD(P)H dehydrogenase, quinone 1 | NM_017000 | 2.76 | 0.037 |
|  |  | **Ccr5** | Chemokine (C-C motif) receptor 5 | NM_053960 | 2.75 | 0.006 |
|  |  | **Hoxa13** | Homeo box A13 | XM_575481 | 2.72 | 0.047 |
|  |  | **Rgd1565321** | Similar to MAP/microtubule affinity-regulating kinase 4 (MAP/microtubule affinity-regulating kinase like 1) | XM_344642 | 2.71 | 0.014 |
|  |  | **Rac2** | Ras-related C3 botulinum toxin substrate 2 (rho family, small GTP binding protein Rac2) | NM_001008384 | 2.64 | 0.008 |
|  |  | **Gcm1** | Glial cells missing homolog 1 (Drosophila) | NM_017186 | 2.63 | 0.002 |
|  |  | **Foxs1** | Forkhead box S1 | NM_001012091 | 2.61 | 0.038 |
|  |  | **Pfkfb1** | 6-phosphofructo-2-kinase/fructose-2,6-biphosphatase 1 | ENSRNOT00000033656 | 2.56 | 0.004 |
|  |  | **Pdgfrb** | Platelet derived growth factor receptor, beta polypeptide | NM_031525 | 2.54 | 0.04 |
|  |  | **S100a9** | S100 calcium binding protein A9 | NM_053587 | 2.49 | 0.029 |
|  |  | **Six1** | SIX homeobox 1 | NM_053759 | 2.48 | 0.025 |
|  |  | **Cyp2d2** | Cytochrome P450, family 2, subfamily d, polypeptide 2 | NM_012730 | 2.48 | 0.021 |
|  |  | **G6pc** | Glucose-6-phosphatase, catalytic subunit | NM_013098 | 2.47 | 0.015 |
|  |  | **Sult2al1** | Sulfotransferase family 2A, dehydroepiandrosterone (DHEA)-preferring-like 1 | NM_012695 | 2.45 | 0.003 |
|  |  | **Loxl4** | Lysyl oxidase-like 4 | NM_001107592 | 2.43 | 0.011 |
|  |  | **Rgd1565390** | Similar to putative protein kinase | XM_344843 | 2.39 | 0.037 |
|  |  | **Cyp2c23** | Cytochrome P450, family 2, subfamily c, polypeptide 23 | NM_031839 | 2.37 | 0.011 |
|  |  | **B4galt1** | UDP-Gal:betaglcnac beta 1,4- galactosyltransferase, polypeptide 1 | NM_053287 | 2.35 | 0.009 |
|  |  | **P4ha3** | Prolyl 4-hydroxylase, alpha polypeptide III | NM_198775 | 2.35 | 0.037 |
|  |  | **Cdkn2b** | Cyclin-dependent kinase inhibitor 2B (p15, inhibits CDK4) | NM_130812 | 2.31 | 0.034 |
|  |  | **Gadd45a** | Growth arrest and DNA-damage-inducible, alpha | NM_024127 | 2.29 | 0.03 |
|  |  | **Rdh7** | Retinol dehydrogenase 7 | NM_133543 | 2.29 | 0.023 |
|  |  | **Mmp25** | Matrix metalloproteinase 25 | XM_002742434 | 2.27 | 0.005 |
|  |  | **Gata6** | GATA binding protein 6 | NM_019185 | 2.27 | 0.035 |
|  |  | **Iyd** | Iodotyrosine deiodinase | NM_001025000 | 2.25 | 0.031 |
|  |  | **Etv2** | Ets variant 2 | ENSRNOT00000031873 | 2.24 | 0.027 |
|  |  | **Adamts5** | ADAM metallopeptidase with thrombospondin type 1 motif, 5 | NM_198761 | 2.24 | 0.005 |
|  |  | **Dpep1** | Dipeptidase 1 (renal) | NM_053591 | 2.23 | 0.035 |
|  |  | **Ferd3l** | Fer3-like (Drosophila) | NM_001108980 | 2.22 | 0.005 |
|  |  | **Dse** | Dermatan sulfate epimerase | NM_001108933 | 2.21 | 0.028 |
|  |  | **Prkcd** | Protein kinase C, delta | NM_133307 | 2.2 | 0.013 |
|  |  | **Cpn1** | Carboxypeptidase N, polypeptide 1 | NM_053526 | 2.18 | 0.045 |
|  |  | **Vegfc** | Vascular endothelial growth factor C | NM_053653 | 2.17 | 0.011 |
|  |  | **Samd4a** | Sterile alpha motif domain containing 4A | NM_001107254 | 2.17 | 0.017 |
|  |  | **Sdr16c5** | Short chain dehydrogenase/reductase family 16C, member 5 | NM_001106634 | 2.17 | 0.004 |
|  |  | **Id1** | Inhibitor of DNA binding 1 | NM_012797 | 2.17 | 0.032 |
|  |  | **Pparg** | Peroxisome proliferator-activated receptor gamma | NM_001145367 | 2.17 | 0.024 |
|  |  | **Mixl1** | Mix1 homeobox-like 1 (Xenopus laevis) | NM_001105979 | 2.15 | 0.005 |
|  |  | **Tgm6** | Transglutaminase 6 | XM_001079039 | 2.14 | 0.004 |
|  |  | **Cpm** | Carboxypeptidase M | NM_001108098 | 2.12 | 0.045 |
|  |  | **Mitf** | Microphthalmia-associated transcription factor | NM_001191089 | 2.12 | 0.019 |
|  |  | **Nme4** | Non-metastatic cells 4, protein expressed in | NM_001109478 | 2.11 | 0.044 |
|  |  | **Atp1b4** | Atpase, (Na+)/K+ transporting, beta 4 polypeptide | NM_053381 | 2.07 | 0.026 |
|  |  | **Casp14** | Caspase 14 | NM_001191776 | 2.06 | 0.019 |
|  |  | **Rgd1564878** | Similar to natural killer cell protease 7 | XM_001058604 | 2.06 | 0.013 |
|  |  | **Enpp3** | Ectonucleotide pyrophosphatase/phosphodiesterase 3 | NM_019370 | 2.06 | 0.043 |
|  |  | **Ugt2b37** | UDP-glucuronosyltransferase 2 family, member 37 | NM_001007264 | 2.04 | 0.017 |
|  |  | **Hand2** | Heart and neural crest derivatives expressed 2 | NM_022696 | 2.03 | 0.038 |
|  |  | **Vrk2** | Vaccinia related kinase 2 | NM_001108366 | 2.03 | 0.025 |
|  |  | **Atp1a4** | Atpase, Na+/K+ transporting, alpha 4 polypeptide | NM_022848 | 2.02 | 0.018 |
|  |  | **Cfb** | Complement factor B | NM_212466 | 2.02 | 0.027 |
|  |  | **Sqrdl** | Sulfide quinone reductase-like (yeast) | NM_001047913 | 2.01 | 0.032 |
|  |  | **Loc682652** | Similar to 60S ribosomal protein L29 (P23) | XM_001062490 | 2,00 | 0.011 |
|  |  | **Hoxc4** | Homeo box C4 | NM_001109884 | 2,00 | 0.018 |
|  |  | **F3** | Coagulation factor III (thromboplastin, tissue factor) | NM_013057 | 2,00 | 0.04 |
|  |  | | | | | |
|  | **Down-regulated** | **Gene Symbol** | **Gene name** | **Accession Number** | **Fold Change 24h** | ***p*-value** |
|  |  | **Agbl1** | ATP/GTP binding protein-like 1 | XM_218798 | 0.27 | 0.015 |
|  |  | **F2** | Coagulation factor II (thrombin) | NM_022924 | 0.3 | 0.009 |
|  |  | **Grin1** | Glutamate receptor, ionotropic, N-methyl D-aspartate 1 | NM_017010 | 0.39 | 0.038 |
|  |  | **Gal3st3** | Galactose-3-O-sulfotransferase 3 | NM_001024290 | 0.4 | 0.042 |
|  |  | **Tesc** | Tescalcin | ENSRNOT00000001490 | 0.41 | 0.02 |
|  |  | **Prss35** | Protease, serine, 35 | NM_001008560 | 0.41 | 0.049 |
|  |  | **Dhcr24** | 24-dehydrocholesterol reductase | NM_001080148 | 0.43 | 0.045 |
|  |  | **Grin2a** | Glutamate receptor, ionotropic, N-methyl D-aspartate 2A | NM_012573 | 0.44 | 0.038 |
|  |  | **Grin2b** | Glutamate receptor, ionotropic, N-methyl D-aspartate 2B | NM_012574 | 0.44 | 0.022 |
|  |  | **Nfil3** | Nuclear factor, interleukin 3 regulated | NM_053727 | 0.45 | 0.048 |
|  |  | **Fam65b** | Family with sequence similarity 65, member B | NM_001014009 | 0.46 | 0.002 |
|  |  | **Prkce** | Protein kinase C, epsilon | NM_017171 | 0.46 | 0.022 |
|  |  | **Kndc1** | Kinase non-catalytic C-lobe domain (KIND) containing 1 | ENSRNOT00000034426 | 0.47 | 0.03 |
|  |  | **Ptgs2** | Prostaglandin-endoperoxide synthase 2 | NM_017232 | 0.48 | 0.008 |
|  |  | **Ambp** | Alpha-1-microglobulin/bikunin precursor | NM_012901 | 0.48 | 0.005 |
|  |  | **Adra1b** | Adrenergic, alpha-1B-, receptor | NM_016991 | 0.48 | 0.022 |
|  |  | **Mapk10** | Mitogen activated protein kinase 10 | NM_012806 | 0.49 | 0.025 |
|  | | | | | | |
| **Neurotransmitter secretion** | **Down-regulated** | **Gene Symbol** | **Gene name** | **Accession Number** | **Fold Change 24h** | ***p*-value** |
|  |  | **Syt1** | Synaptotagmin I | NM_001033680 | 0.44 | 0.031 |
|  | | | | | | |
| **Signaling Pathways** | **Up-regulated** | **Gene Symbol** | **Gene name** | **Accession Number** | **Fold Change 24h** | ***p*-value** |
|  |  | **Ctgf** | Connective tissue growth factor | NM_022266 | 9.84 | 0.012 |
|  |  | **Hmga2** | High mobility group AT-hook 2 | NM_032070 | 6.99 | 0.023 |
|  |  | **Olr1297** | Olfactory receptor 1297 | NM_001000461 | 5.93 | 0.045 |
|  |  | **Calca** | Calcitonin-related polypeptide alpha | NM_017338 | 5.11 | 0.032 |
|  |  | **Anxa1** | Annexin A1 | NM_012904 | 4.62 | 0.018 |
|  |  | **Il5** | Interleukin 5 | NM_021834 | 4.57 | 0.026 |
|  |  | **Casp1** | Caspase 1 | NM_012762 | 4.56 | 0.026 |
|  |  | **Npvf** | Neuropeptide VF precursor | NM_023952 | 3.99 | 0.021 |
|  |  | **Olr1595** | Olfactory receptor 1595 | NM_001000500 | 3.93 | 0.011 |
|  |  | **Olr60** | Olfactory receptor 60 | NM_001000748 | 3.88 | 0.048 |
|  |  | **Rab32** | RAB32, member RAS oncogene family | NM_001108902 | 3.77 | 0.031 |
|  |  | **Olr907** | Olfactory receptor 907 | NM_001001357 | 3.75 | 0.041 |
|  |  | **Olr1443** | Olfactory receptor 1443 | NM_001000018 | 3.69 | 0.009 |
|  |  | **Rgd1564327** | Similar to integrin alpha 8 | NM_001173972 | 3.52 | 0.027 |
|  |  | **Olr1666** | Olfactory receptor 1666 | NM_001000108 | 3.31 | 0.026 |
|  |  | **Itgb3** | Integrin, beta 3 | NM_153720 | 3.28 | 0.039 |
|  |  | **Olr1185** | Olfactory receptor 1185 | NM_001000982 | 3.27 | 0.009 |
|  |  | **Gpr39** | G protein-coupled receptor 39 | NM_001100943 | 3.25 | 0.021 |
|  |  | **Olr1261** | Olfactory receptor 1261 | NM_001000804 | 3.1 | 0.04 |
|  |  | **Cd44** | Cd44 molecule | NM_012924 | 3.07 | 0.032 |
|  |  | **Plcb2** | Phospholipase C, beta 2 | NM_053478 | 2.94 | 0.034 |
|  |  | **Olr1700** | Olfactory receptor 1700 | NM_001001113 | 2.88 | 0.028 |
|  |  | **Olr834** | Olfactory receptor 834 | NM_001000407 | 2.82 | 0.022 |
|  |  | **Ccr5** | Chemokine (C-C motif) receptor 5 | NM_053960 | 2.75 | 0.006 |
|  |  | **Olr1453** | Olfactory receptor 1453 | NM_001000772 | 2.74 | 0.043 |
|  |  | **Olr1012** | Olfactory receptor 1012 | NM_001000071 | 2.73 | 0.002 |
|  |  | **Hoxa13** | Homeo box A13 | XM_575481 | 2.72 | 0.047 |
|  |  | **Rac2** | Ras-related C3 botulinum toxin substrate 2 (rho family, small GTP binding protein Rac2) | NM_001008384 | 2.64 | 0.008 |
|  |  | **Pfkfb1** | 6-phosphofructo-2-kinase/fructose-2,6-biphosphatase 1 | ENSRNOT00000033656 | 2.56 | 0.004 |
|  |  | **Pdgfrb** | Platelet derived growth factor receptor, beta polypeptide | NM_031525 | 2.54 | 0.04 |
|  |  | **Olr1361** | Olfactory receptor 1361 | NM_173333 | 2.5 | 0.008 |
|  |  | **Il1rl1** | Interleukin 1 receptor-like 1 | NM_001127689 | 2.39 | 0.022 |
|  |  | **Olr326** | Olfactory receptor 326 | NM_001000248 | 2.34 | 0.025 |
|  |  | **Olr1061** | Olfactory receptor 1061 | NM_001000065 | 2.32 | 0.032 |
|  |  | **Cdkn2b** | Cyclin-dependent kinase inhibitor 2B (p15, inhibits CDK4) | NM_130812 | 2.31 | 0.034 |
|  |  | **Gadd45a** | Growth arrest and DNA-damage-inducible, alpha | NM_024127 | 2.29 | 0.03 |
|  |  | **Olr1368** | Olfactory receptor 1368 | NM_214825 | 2.28 | 0.026 |
|  |  | **Olr1369** | Olfactory receptor 1369 | NM_001000494 | 2.28 | 0.023 |
|  |  | **Gata6** | GATA binding protein 6 | NM_019185 | 2.27 | 0.035 |
|  |  | **Olr1512** | Olfactory receptor 1512 | NM_001001350 | 2.26 | 0.014 |
|  |  | **Olr1174** | Olfactory receptor 1174 | NM_001001016 | 2.25 | 0.023 |
|  |  | **Asb15** | Ankyrin repeat and SOCS box-containing protein 15 | ENSRNOT00000008917 | 2.23 | 0.004 |
|  |  | **Prkcd** | Protein kinase C, delta | NM_133307 | 2.2 | 0.013 |
|  |  | **Olr718** | Olfactory receptor 718 | NM_001000361 | 2.2 | 0.024 |
|  |  | **Vegfc** | Vascular endothelial growth factor C | NM_053653 | 2.17 | 0.011 |
|  |  | **Olr185** | Olfactory receptor 185 | NM_001000183 | 2.17 | 0.004 |
|  |  | **Id1** | Inhibitor of DNA binding 1 | NM_012797 | 2.17 | 0.032 |
|  |  | **Rgd1563046** | Similar to cerberus-like | NM_001115031 | 2.16 | 0.031 |
|  |  | **Vom1r52** | Vomeronasal 1 receptor 52 | NM_001008930 | 2.14 | 0.018 |
|  |  | **Olr1022** | Olfactory receptor 1022 | NM_001001075 | 2.14 | 0.043 |
|  |  | **Olr1440** | Olfactory receptor 1440 | NM_001000017 | 2.13 | 0.031 |
|  |  | **Loc685368** | Similar to Ral guanine nucleotide dissociation stimulator (ralgef) (ralgds) | XM_001063509 | 2.12 | 0.019 |
|  |  | **Mitf** | Microphthalmia-associated transcription factor | NM_001191089 | 2.12 | 0.019 |
|  |  | **Olr464** | Olfactory receptor 464 | NM_001000296 | 2.12 | 0.018 |
|  |  | **Olr713** | Olfactory receptor 713 | NM_001000360 | 2.08 | 0.022 |
|  |  | **Olr768** | Olfactory receptor 768 | NM_001000919 | 2.08 | 0.007 |
|  |  | **Olr306** | Olfactory receptor 306 | NM_001000766 | 2.07 | 0.003 |
|  |  | **Olr233** | Olfactory receptor 233 | NM_001000207 | 2.07 | 0.036 |
|  |  | **Olr866** | Olfactory receptor 866 | NM_001000411 | 2.04 | 0.031 |
|  |  | **Chrm2** | Cholinergic receptor, muscarinic 2 | NM_031016 | 2.01 | 0.012 |
|  |  | **Olr127** | Olfactory receptor 127 | NM_001001026 | 2.01 | 0.006 |
|  |  | | | | | |
|  | **Down-regulated** | **Gene Symbol** | **Gene name** | **Accession Number** | **Fold Change 24h** | ***p*-value** |
|  |  | **Olr1256** | Olfactory receptor 1256 | NM_001001086 | 0.17 | 0.011 |
|  |  | **Gpr83** | G protein-coupled receptor 83 | NM_080411 | 0.3 | 0.031 |
|  |  | **F2** | Coagulation factor II (thrombin) | NM_022924 | 0.3 | 0.009 |
|  |  | **Cd27** | CD27 molecule | NM_001024335 | 0.31 | 0.035 |
|  |  | **Grin1** | Glutamate receptor, ionotropic, N-methyl D-aspartate 1 | NM_017010 | 0.39 | 0.038 |
|  |  | **Dhcr24** | 24-dehydrocholesterol reductase | NM_001080148 | 0.43 | 0.045 |
|  |  | **Grin2a** | Glutamate receptor, ionotropic, N-methyl D-aspartate 2A | NM_012573 | 0.44 | 0.038 |
|  |  | **Grin2b** | Glutamate receptor, ionotropic, N-methyl D-aspartate 2B | NM_012574 | 0.44 | 0.022 |
|  |  | **Prkce** | Protein kinase C, epsilon | NM_017171 | 0.46 | 0.022 |
|  |  | **Gabra3** | Gamma-aminobutyric acid (GABA) A receptor, alpha 3 | NM_017069 | 0.47 | 0.004 |
|  |  | **Kndc1** | Kinase non-catalytic C-lobe domain (KIND) containing 1 | ENSRNOT00000034426 | 0.47 | 0.03 |
|  |  | **Adra1b** | Adrenergic, alpha-1B-, receptor | NM_016991 | 0.48 | 0.022 |
|  |  | **Mapk10** | Mitogen activated protein kinase 10 | NM_012806 | 0.49 | 0.025 |
|  |  | **Rab6b** | RAB6B, member RAS oncogene family | NM_001108775 | 0.5 | 0.039 |
|  | | | | | | |
| **Synapse** | **Up-regulated** | **Gene Symbol** | **Gene name** | **Accession Number** | **Fold Change 24h** | ***p*-value** |
|  |  | **Cald1** | Caldesmon 1 | NM_013146 | 2.55 | 0.011 |
|  |  | **Samd4a** | Sterile alpha motif domain containing 4A | NM_001107254 | 2.17 | 0.017 |
|  |  | **Chrm2** | Cholinergic receptor, muscarinic 2 | NM_031016 | 2.01 | 0.012 |
|  |  | | | | | |
|  | **Down-regulated** | **Gene Symbol** | **Gene name** | **Accession Number** | **Fold Change 24h** | ***p*-value** |
|  |  | **Grin1** | Glutamate receptor, ionotropic, N-methyl D-aspartate 1 | NM_017010 | 0.39 | 0.038 |
|  |  | **Grin2a** | Glutamate receptor, ionotropic, N-methyl D-aspartate 2A | NM_012573 | 0.44 | 0.038 |
|  |  | **Syt1** | Synaptotagmin I | NM_001033680 | 0.44 | 0.031 |
|  |  | **Grin2b** | Glutamate receptor, ionotropic, N-methyl D-aspartate 2B | NM_012574 | 0.44 | 0.022 |
|  |  | **Clstn2** | Calsyntenin 2 | NM_134377 | 0.44 | 0.018 |
|  |  | **Cabp1** | Calcium binding protein 1 | NM_001033676 | 0.47 | 0.041 |
|  |  | **Gabra3** | Gamma-aminobutyric acid (GABA) A receptor, alpha 3 | NM_017069 | 0.47 | 0.004 |
|  |  | **Gabrb3** | Gamma-aminobutyric acid (GABA) A receptor, beta 3 | NM_017065 | 0.48 | 0.015 |
|  | | | | | | |
| **Transcription** | **Up-regulated** | **Gene Symbol** | **Gene name** | **Accession Number** | **Fold Change 24h** | ***p*-value** |
|  |  | **Ferd3l** | Connective tissue growth factor | NM_022266 | 9.84 | 0.012 |
|  |  | **Hmga2** | High mobility group AT-hook 2 | NM_032070 | 6.99 | 0.023 |
|  |  | **Lhx1** | LIM homeobox 1 | NM_145880 | 6.02 | 0.022 |
|  |  | **Loc687736** | Similar to double homeobox, 4 | XM_001079968 | 5.48 | 0.05 |
|  |  | **Calca** | Calcitonin-related polypeptide alpha | NM_017338 | 5.11 | 0.032 |
|  |  | **Rgd1563833** | Similar to putative homeobox protein | ENSRNOT00000066263 | 4.82 | 0.029 |
|  |  | **Rgd1564033** | Similar to Homeobox protein OTX1 | XM_001059672 | 4.73 | 0.041 |
|  |  | **Mycs** | Myc-like oncogene, s-myc protein | NM_021837 | 4.25 | 0.042 |
|  |  | **Loc100365510** | Homeobox protein-like | XM_002727408 | 3.7 | 0.033 |
|  |  | **Loc100363448** | Double homeobox, 1-like | XM_002728977 | 3.62 | 0.042 |
|  |  | **Hoxb7** | Homeo box B7 | NM_001017480 | 3.41 | 0.038 |
|  |  | **Loc687536** | Similar to Forkhead box protein F1 (Forkhead-related protein FKHL5) (Forkhead-related transcription factor 1) (FREAC-1) (Hepatocyte nuclear factor 3 forkhead homolog 8) (HFH-8) | XM_001079002 | 3.17 | 0.026 |
|  |  | **Icam1** | Intercellular adhesion molecule 1 | NM_012967 | 2.91 | 0.033 |
|  |  | **Nr6a1** | Nuclear receptor subfamily 6, group A, member 1 | XM_001060553 | 2.87 | 0.038 |
|  |  | **Hoxa13** | Homeo box A13 | XM_575481 | 2.72 | 0.047 |
|  |  | **Gcm1** | Glial cells missing homolog 1 (Drosophila) | NM_017186 | 2.63 | 0.002 |
|  |  | **Foxs1** | Forkhead box S1 | NM_001012091 | 2.61 | 0.038 |
|  |  | **Six1** | SIX homeobox 1 | NM_053759 | 2.48 | 0.025 |
|  |  | **Cdkn2b** | Cyclin-dependent kinase inhibitor 2B (p15, inhibits CDK4) | NM_130812 | 2.31 | 0.034 |
|  |  | **Gata6** | GATA binding protein 6 | NM_019185 | 2.27 | 0.035 |
|  |  | **Etv2** | Ets variant 2 | ENSRNOT00000031873 | 2.24 | 0.027 |
|  |  | **Id1** | Inhibitor of DNA binding 1 | NM_012797 | 2.17 | 0.032 |
|  |  | **Pparg** | Peroxisome proliferator-activated receptor gamma | NM_001145367 | 2.17 | 0.024 |
|  |  | **Mixl1** | Mix1 homeobox-like 1 (Xenopus laevis) | NM_001105979 | 2.15 | 0.005 |
|  |  | **Mitf** | Microphthalmia-associated transcription factor | NM_001191089 | 2.12 | 0.019 |
|  |  | **Atp1b4** | Atpase, (Na+)/K+ transporting, beta 4 polypeptide | NM_053381 | 2.07 | 0.026 |
|  |  | **Hand2** | Heart and neural crest derivatives expressed 2 | NM_022696 | 2.03 | 0.038 |
|  |  | **Hoxc4** | Homeo box C4 | NM_001109884 | 2,00 | 0.018 |
|  |  | | | | | |
|  | **Down-regulated** | **Gene Symbol** | **Gene name** | **Accession Number** | **Fold Change 24h** | ***p*-value** |
|  |  | **Grin1** | Glutamate receptor, ionotropic, N-methyl D-aspartate 1 | NM_017010 | 0.39 | 0.038 |
|  |  | **Tesc** | Tescalcin | ENSRNOT00000001490 | 0.41 | 0.02 |
|  |  | **Nfil3** | Nuclear factor, interleukin 3 regulated | NM_053727 | 0.45 | 0.048 |
|  | | | | | | |
| **Receptor Activity** | **Up-regulated** | **Gene Symbol** | **Gene name** | **Accession Number** | **Fold Change 24h** | ***p*-value** |
|  |  | **Olr1012** | Connective tissue growth factor | NM_022266 | 9.84 | 0.012 |
|  |  | **Mrgprf** | MAS-related GPR, member F | NM_153722 | 6.88 | 0,000 |
|  |  | **Vom2r73** | Vomeronasal 2 receptor, 73 | NM_001099486 | 6.16 | 0.03 |
|  |  | **Olr1297** | Olfactory receptor 1297 | NM_001000461 | 5.93 | 0.045 |
|  |  | **Loc680894** | Similar to putative pheromone receptor (Go-VN4) | XM_001059364 | 5.11 | 0.025 |
|  |  | **Olr1** | Oxidized low density lipoprotein (lectin-like) receptor 1 | NM_133306 | 4.1 | 0.016 |
|  |  | **Olr1595** | Olfactory receptor 1595 | NM_001000500 | 3.93 | 0.011 |
|  |  | **Ccr6** | Chemokine (C-C motif) receptor 6 | NM_001013145 | 3.88 | 0.013 |
|  |  | **Olr60** | Olfactory receptor 60 | NM_001000748 | 3.88 | 0.048 |
|  |  | **Olr907** | Olfactory receptor 907 | NM_001001357 | 3.75 | 0.041 |
|  |  | **Olr1443** | Olfactory receptor 1443 | NM_001000018 | 3.69 | 0.009 |
|  |  | **Rgd1564327** | Similar to integrin alpha 8 | NM_001173972 | 3.52 | 0.027 |
|  |  | **Vom2r3** | Vomeronasal 2 receptor, 3 | NM_001099460 | 3.38 | 0.009 |
|  |  | **Olr1666** | Olfactory receptor 1666 | NM_001000108 | 3.31 | 0.026 |
|  |  | **Itgb3** | Integrin, beta 3 | NM_153720 | 3.28 | 0.039 |
|  |  | **Olr1185** | Olfactory receptor 1185 | NM_001000982 | 3.27 | 0.009 |
|  |  | **Gpr39** | G protein-coupled receptor 39 | NM_001100943 | 3.25 | 0.021 |
|  |  | **Olr1261** | Olfactory receptor 1261 | NM_001000804 | 3.1 | 0.04 |
|  |  | **Cd44** | Cd44 molecule | NM_012924 | 3.07 | 0.032 |
|  |  | **Ly49i4** | Ly49 inhibitory receptor 4 | NM_001009495 | 3.06 | 0.021 |
|  |  | **Olr1700** | Olfactory receptor 1700 | NM_001001113 | 2.88 | 0.028 |
|  |  | **Nr6a1** | Nuclear receptor subfamily 6, group A, member 1 | XM_001060553 | 2.87 | 0.038 |
|  |  | **Olr834** | Olfactory receptor 834 | NM_001000407 | 2.82 | 0.022 |
|  |  | **Lyve1** | Lymphatic vessel endothelial hyaluronan receptor 1 | NM_001106286 | 2.8 | 0.038 |
|  |  | **Ccr5** | Chemokine (C-C motif) receptor 5 | NM_053960 | 2.75 | 0.006 |
|  |  | **Olr1453** | Olfactory receptor 1453 | NM_001000772 | 2.74 | 0.043 |
|  |  | **Pdgfrb** | Platelet derived growth factor receptor, beta polypeptide | NM_031525 | 2.54 | 0.04 |
|  |  | **Olr1361** | Olfactory receptor 1361 | NM_173333 | 2.5 | 0.008 |
|  |  | **Vom2r49** | Vomeronasal 2 receptor, 49 | NM_001099515 | 2.48 | 0.007 |
|  |  | **Loxl4** | Lysyl oxidase-like 4 | NM_001107592 | 2.43 | 0.011 |
|  |  | **Il1rl1** | Interleukin 1 receptor-like 1 | NM_001127689 | 2.39 | 0.022 |
|  |  | **Olr326** | Olfactory receptor 326 | NM_001000248 | 2.34 | 0.025 |
|  |  | **Clec4b2** | C-type lectin domain family 4, member b2 | NM_001005896 | 2.34 | 0.045 |
|  |  | **Olr1061** | Olfactory receptor 1061 | NM_001000065 | 2.32 | 0.032 |
|  |  | **Olr1368** | Olfactory receptor 1368 | NM_214825 | 2.28 | 0.026 |
|  |  | **Olr1369** | Olfactory receptor 1369 | NM_001000494 | 2.28 | 0.023 |
|  |  | **Cxcl16** | Chemokine (C-X-C motif) ligand 16 | NM_001017478 | 2.28 | 0.015 |
|  |  | **Olr1512** | Olfactory receptor 1512 | NM_001001350 | 2.26 | 0.014 |
|  |  | **Olr1174** | Olfactory receptor 1174 | NM_001001016 | 2.25 | 0.023 |
|  |  | **Olr718** | Olfactory receptor 718 | NM_001000361 | 2.2 | 0.024 |
|  |  | **Olr185** | Olfactory receptor 185 | NM_001000183 | 2.17 | 0.004 |
|  |  | **Pparg** | Peroxisome proliferator-activated receptor gamma | NM_001145367 | 2.17 | 0.024 |
|  |  | **Vom1r52** | Vomeronasal 1 receptor 52 | NM_001008930 | 2.14 | 0.018 |
|  |  | **Olr1022** | Olfactory receptor 1022 | NM_001001075 | 2.14 | 0.043 |
|  |  | **Olr1440** | Olfactory receptor 1440 | NM_001000017 | 2.13 | 0.031 |
|  |  | **Olr464** | Olfactory receptor 464 | NM_001000296 | 2.12 | 0.018 |
|  |  | **Olr713** | Olfactory receptor 713 | NM_001000360 | 2.08 | 0.022 |
|  |  | **Olr768** | Olfactory receptor 768 | NM_001000919 | 2.08 | 0.007 |
|  |  | **Olr306** | Olfactory receptor 306 | NM_001000766 | 2.07 | 0.003 |
|  |  | **Olr233** | Olfactory receptor 233 | NM_001000207 | 2.07 | 0.036 |
|  |  | **Enpp3** | Ectonucleotide pyrophosphatase/phosphodiesterase 3 | NM_019370 | 2.06 | 0.043 |
|  |  | **Olr866** | Olfactory receptor 866 | NM_001000411 | 2.04 | 0.031 |
|  |  | **Taar4** | Trace amine-associated receptor 4 | RatNM_175583 | 2.03 | 0.039 |
|  |  | **Chrm2** | Cholinergic receptor, muscarinic 2 | NM_031016 | 2.01 | 0.012 |
|  |  | **Olr127** | Olfactory receptor 127 | NM_001001026 | 2.01 | 0.006 |
|  |  | **Vom2r71** | Vomeronasal 2 receptor, 71 | NM_001099516 | 2.01 | 0.029 |
|  |  | | | | | |
|  | **Down-regulated** | **Gene Symbol** | **Gene name** | **Accession Number** | **Fold Change 24h** | ***p*-value** |
|  |  | **Olr1256** | Olfactory receptor 1256 | NM_001001086 | 0.17 | 0.011 |
|  |  | **Gpr83** | G protein-coupled receptor 83 | NM_080411 | 0.3 | 0.031 |
|  |  | **F2** | Coagulation factor II (thrombin) | NM_022924 | 0.3 | 0.009 |
|  |  | **Cd27** | CD27 molecule | NM_001024335 | 0.31 | 0.035 |
|  |  | **Grin1** | Glutamate receptor, ionotropic, N-methyl D-aspartate 1 | NM_017010 | 0.39 | 0.038 |
|  |  | **Trpc5** | Transient receptor potential cation channel, subfamily C, member 5 | NM_080898 | 0.41 | 0.048 |
|  |  | **Grin2a** | Glutamate receptor, ionotropic, N-methyl D-aspartate 2A | NM_012573 | 0.44 | 0.038 |
|  |  | **Grin2b** | Glutamate receptor, ionotropic, N-methyl D-aspartate 2B | NM_012574 | 0.44 | 0.022 |
|  |  | **Gabra3** | Gamma-aminobutyric acid (GABA) A receptor, alpha 3 | NM_017069 | 0.47 | 0.004 |
|  |  | **Adra1b** | Adrenergic, alpha-1B-, receptor | NM_016991 | 0.48 | 0.022 |
|  |  | **Gabrb3** | Gamma-aminobutyric acid (GABA) A receptor, beta 3 | NM_017065 | 0.48 | 0.015 |
|  |  | **Gpr123** | G protein-coupled receptor 123 | NM_001107559 | 0.49 | 0.028 |
|  | | | | | | |
| **Glutamate Secretion** | **Down-regulated** | **Gene Symbol** | **Gene name** | **Accession Number** | **Fold Change 24h** | ***p*-value** |
|  |  | **Grin2b** | Glutamate receptor, ionotropic, N-methyl D-aspartate 2B | NM_012574 | 0.44 | 0.022 |
|  | | | | | | |
| **RNA Biosynthetic Process** | **Up-regulated** | **Gene Symbol** | **Gene name** | **Accession Number** | **Fold Change 24h** | ***p*-value** |
|  |  | **Cdkn2b** | Connective tissue growth factor | NM_022266 | 9.84 | 0.012 |
|  |  | **Hmga2** | High mobility group AT-hook 2 | NM_032070 | 6.99 | 0.023 |
|  |  | **Lhx1** | LIM homeobox 1 | NM_145880 | 6.02 | 0.022 |
|  |  | **Loc687736** | Similar to double homeobox, 4 | XM_001079968 | 5.48 | 0.05 |
|  |  | **Rgd1563833** | Similar to putative homeobox protein | ENSRNOT00000066263 | 4.82 | 0.029 |
|  |  | **Rgd1564033** | Similar to Homeobox protein OTX1 | XM_001059672 | 4.73 | 0.041 |
|  |  | **Mycs** | Myc-like oncogene, s-myc protein | NM_021837 | 4.25 | 0.042 |
|  |  | **Loc100365510** | Homeobox protein-like | XM_002727408 | 3.7 | 0.033 |
|  |  | **Loc100363448** | Double homeobox, 1-like | XM_002728977 | 3.62 | 0.042 |
|  |  | **Hoxb7** | Homeo box B7 | NM_001017480 | 3.41 | 0.038 |
|  |  | **Loc687536** | Similar to Forkhead box protein F1 (Forkhead-related protein FKHL5) (Forkhead-related transcription factor 1) (FREAC-1) (Hepatocyte nuclear factor 3 forkhead homolog 8) (HFH-8) | XM_001079002 | 3.17 | 0.026 |
|  |  | **Icam1** | Intercellular adhesion molecule 1 | NM_012967 | 2.91 | 0.033 |
|  |  | **Nr6a1** | Nuclear receptor subfamily 6, group A, member 1 | XM_001060553 | 2.87 | 0.038 |
|  |  | **Hoxa13** | Homeo box A13 | XM_575481 | 2.72 | 0.047 |
|  |  | **Gcm1** | Glial cells missing homolog 1 (Drosophila) | NM_017186 | 2.63 | 0.002 |
|  |  | **Foxs1** | Forkhead box S1 | NM_001012091 | 2.61 | 0.038 |
|  |  | **Six1** | SIX homeobox 1 | NM_053759 | 2.48 | 0.025 |
|  |  | **Gata6** | GATA binding protein 6 | NM_019185 | 2.27 | 0.035 |
|  |  | **Etv2** | Ets variant 2 | ENSRNOT00000031873 | 2.24 | 0.027 |
|  |  | **Id1** | Inhibitor of DNA binding 1 | NM_012797 | 2.17 | 0.032 |
|  |  | **Pparg** | Peroxisome proliferator-activated receptor gamma | NM_001145367 | 2.17 | 0.024 |
|  |  | **Mixl1** | Mix1 homeobox-like 1 (Xenopus laevis) | NM_001105979 | 2.15 | 0.005 |
|  |  | **Mitf** | Microphthalmia-associated transcription factor | NM_001191089 | 2.12 | 0.019 |
|  |  | **Hand2** | Heart and neural crest derivatives expressed 2 | NM_022696 | 2.03 | 0.038 |
|  |  | **Hoxc4** | Homeo box C4 | NM_001109884 | 2,00 | 0.018 |
|  |  | | | | | |
|  | **Down-regulated** | **Gene Symbol** | **Gene name** | **Accession Number** | **Fold Change 24h** | ***p*-value** |
|  |  | **Grin1** | Glutamate receptor, ionotropic, N-methyl D-aspartate 1 | NM_017010 | 0.39 | 0.038 |
|  |  | **Tesc** | Tescalcin | ENSRNOT00000001490 | 0.41 | 0.02 |
|  |  | **Nfil3** | Nuclear factor, interleukin 3 regulated | NM_053727 | 0.45 | 0.048 |
|  | | | | | | |
| **Oxidative Stress** | **Up-regulated** | **Gene Symbol** | **Gene name** | **Accession Number** | **Fold Change 24h** | ***p*-value** |
|  |  | **F3** | Connective tissue growth factor | NM_022266 | 9.84 | 0.012 |
|  |  | **Anxa1** | Annexin A1 | NM_012904 | 4.62 | 0.018 |
|  |  | **Ucp3** | Uncoupling protein 3 (mitochondrial, proton carrier) | NM_013167 | 4.16 | 0.012 |
|  |  | **Olr1** | Oxidized low density lipoprotein (lectin-like) receptor 1 | NM_133306 | 4.1 | 0.016 |
|  |  | **Ptgs1** | Prostaglandin-endoperoxide synthase 1 | NM_017043 | 2.92 | 0.004 |
|  |  | **Nqo1** | NAD(P)H dehydrogenase, quinone 1 | NM_017000 | 2.76 | 0.037 |
|  |  | **Pdgfrb** | Platelet derived growth factor receptor, beta polypeptide | NM_031525 | 2.54 | 0.04 |
|  |  | **Prkcd** | Protein kinase C, delta | NM_133307 | 2.2 | 0.013 |
|  |  | | | | | |
|  | **Down-regulated** | **Gene Symbol** | **Gene name** | **Accession Number** | **Fold Change 24h** | ***p*-value** |
|  |  | **Dhcr24** | 24-dehydrocholesterol reductase | NM_001080148 | 0.43 | 0.045 |
|  |  | **Ptgs2** | Prostaglandin-endoperoxide synthase 2 | NM_017232 | 0.48 | 0.008 |
|  |  | **Slc8a1** | Solute carrier family 8 (sodium/calcium exchanger), member 1 | NM_019268 | 0.5 | 0.022 |
