# Supplementary material for: In Vitro Ischemia Triggers a Transcriptional Response to Down-Regulate Synaptic Proteins in Hippocampal Neurons
Source: PLoS One. 2014 Jun 24;9(6):e99958. doi: 10.1371/journal.pone.0099958 (PMC4069008; doi:10.1371/journal.pone.0099958)
Supplement: Table S7 — List of genes up-regulated and down-regulated at both 7 h and 24 h after OGD for different ontological classes. Gene ontology analyses included genes that had a p-value <0.05 and a fold change of 2.0 and were performed using GoMiner. Classes were selected manually. Note that some genes are included in more than one class. (DOCX) [file pone.0099958.s008.docx]

| **Apoptosis** | **Up-regulated** | **Gene Symbol** | **Gene name** | **Accession Number** | **Fold Change 7h** | ***p*-value 7h** | **Fold Change 24h** | ***p*-value 24h** |
| --- | --- | --- | --- | --- | --- | --- | --- | --- |
|  |  | Gal | Galanin prepropeptide | NM_033237 | 9.39 | 0.004 | 5.6 | 0.029 |
|  |  | Serpine1 | Serpin peptidase inhibitor, clade E (nexin, plasminogen activator inhibitor type 1), member 1 | NM_012620 | 5.03 | 0.018 | 9.77 | 0.028 |
|  |  | Foxb1 | Forkhead box B1 | NM_001013248 | 5.62 | 0.048 | 6.39 | 0.035 |
|  |  | Epha2 | Eph receptor A2 | NM_001108977 | 2.91 | 0.023 | 6.39 | 0.026 |
|  |  | Ripk3 | Receptor-interacting serine-threonine kinase 3 | NM_139342 | 2.98 | 0.049 | 5.02 | 0.025 |
|  |  | Cd44 | Cd44 molecule | NM_012924 | 2.2 | 0.006 | 3.32 | 0.036 |
|  |  | Il1rn | Interleukin 1 receptor antagonist | NM_022194 | 2.79 | 0.001 | 2.2 | 0.024 |
|  |  | | | | | | | |
|  | **Down-regulated** | **Gene Symbol** | **Gene name** | **Accession Number** | **Fold Change 7h** | ***p*-value 7h** | **Fold Change 24h** | ***p*-value 24h** |
|  |  | Acvr1c | Activin A receptor, type IC | NM_139090 | 0.49 | 0.023 | 0.3 | 0.043 |
|  |  | Pml | Promyelocytic leukemia | XM_236296 | 0.43 | 0.008 | 0.44 | 0.036 |
|  | | | | | | | | |
| **Inflammatory response** | **Up-regulated** | **Gene Symbol** | **Gene name** | **Accession Number** | **Fold Change 7h** | ***p*-value 7h** | **Fold Change 24h** | ***p*-value 24h** |
|  |  | Il1rl1 | Interleukin 1 receptor-like 1 | NM_013037 | 13.01 | 0.03 | 47.02 | 0.001 |
|  |  | Reg3b | Regenerating islet-derived 3 beta | NM_053289 | 17.43 | 0.015 | 29,00 | 0.007 |
|  |  | Cxcl1 | Chemokine (C-X-C motif) ligand 1 (melanoma growth stimulating activity, alpha) | NM_030845 | 8.35 | 0.002 | 11.99 | 0.011 |
|  |  | Gal | Galanin prepropeptide | NM_033237 | 9.39 | 0.004 | 5.6 | 0.029 |
|  |  | Serpine1 | Serpin peptidase inhibitor, clade E (nexin, plasminogen activator inhibitor type 1), member 1 | NM_012620 | 5.03 | 0.018 | 9.77 | 0.028 |
|  |  | Gpx2 | Glutathione peroxidase 2 | NM_183403 | 5.91 | 0.008 | 8.85 | 0.004 |
|  |  | Cd44 | Cd44 molecule | NM_012924 | 2.2 | 0.006 | 3.32 | 0.036 |
|  |  | Il1rn | Interleukin 1 receptor antagonist | NM_022194 | 2.79 | 0.001 | 2.2 | 0.024 |
|  | | | | | | | | |
| **Ion Transmembr. Transporter Activity** | **Down-regulated** | **Gene Symbol** | **Gene name** | **Accession Number** | **Fold Change 7h** | ***p*-value 7h** | **Fold Change 24h** | ***p*-value 24h** |
|  |  | Atp13a2 | Activin A receptor, type IC | NM_139090 | 0.49 | 0.023 | 0.3 | 0.043 |
|  | | | | | | | | |
| **Metabolic Process** |  | **Gene Symbol** | **Gene name** | **Accession Number** | **Fold Change 7h** | ***p*-value 7h** | **Fold Change 24h** | ***p*-value 24h** |
|  |  | Gal | Galanin prepropeptide | NM_033237 | 9.39 | 0.004 | 5.6 | 0.029 |
|  |  | Serpine1 | Serpin peptidase inhibitor, clade E (nexin, plasminogen activator inhibitor type 1), member 1 | NM_012620 | 5.03 | 0.018 | 9.77 | 0.028 |
|  |  | Gpx2 | Glutathione peroxidase 2 | NM_183403 | 5.91 | 0.008 | 8.85 | 0.004 |
|  |  | Plau | Plasminogen activator, urokinase | NM_013085 | 4.85 | 0.003 | 9.07 | 0.03 |
|  |  | Epha2 | Eph receptor A2 | NM_001108977 | 2.91 | 0.023 | 6.39 | 0.026 |
|  | **Up-regulated** | Ripk3 | Receptor-interacting serine-threonine kinase 3 | NM_139342 | 2.98 | 0.049 | 5.02 | 0.025 |
|  |  | Lipg | Lipase, endothelial | NM_001012741 | 3,00 | 0.037 | 4.08 | 0.012 |
|  |  | Runx2 | Runt-related transcription factor 2 | NM_053470 | 2.86 | 0,000 | 2.98 | 0.049 |
|  |  | Hopx | HOP homeobox | NM_133621 | 2.08 | 0.032 | 3.47 | 0.037 |
|  |  | Cd44 | Cd44 molecule | NM_012924 | 2.2 | 0.006 | 3.32 | 0.036 |
|  |  | Il1rn | Interleukin 1 receptor antagonist | NM_022194 | 2.79 | 0.001 | 2.2 | 0.024 |
|  |  | Adamts7 | ADAM metallopeptidase with thrombospondin type 1 motif, 7 | NM_001047101 | 2.29 | 0.018 | 2.64 | 0.023 |
|  |  | Mst1 | Macrophage stimulating 1 (hepatocyte growth factor-like) | NM_024352 | 2.24 | 0.044 | 2.61 | 0.004 |
|  |  | Tgif1 | TGFB-induced factor homeobox 1 | NM_001015020 | 2.11 | 0.041 | 2.73 | 0.04 |
|  |  | Lbxcor1 | LBXCOR1 homolog (mouse) | XM_002727063 | 2.35 | 0.042 | 2.49 | 0.024 |
|  |  | | | | | | | |
|  | **Down-regulated** | **Gene Symbol** | **Gene name** | **Accession Number** | **Fold Change 7h** | ***p*-value 7h** | **Fold Change 24h** | ***p*-value 24h** |
|  |  | Mmp28 | Matrix metallopeptidase 28 | NM_001079888 | 0.2 | 0.019 | 0.24 | 0.01 |
|  |  | Ube2ql1 | Ubiquitin-conjugating enzyme E2Q family-like 1 | NM_001145163 | 0.31 | 0.007 | 0.36 | 0.038 |
|  |  | Far2 | Fatty acyl coa reductase 2 | ENSRNOT00000002528 | 0.33 | 0.001 | 0.35 | 0.027 |
|  |  | Acvr1c | Activin A receptor, type IC | NM_139090 | 0.49 | 0.023 | 0.3 | 0.043 |
|  |  | Gpr26 | G protein-coupled receptor 26 | NM_138841 | 0.48 | 0.013 | 0.33 | 0.03 |
|  |  | St8sia5 | ST8 alpha-N-acetyl-neuraminide alpha-2,8-sialyltransferase 5 | NM_213628 | 0.49 | 0.007 | 0.37 | 0.016 |
|  |  | Pml | Promyelocytic leukemia | XM_236296 | 0.43 | 0.008 | 0.44 | 0.036 |
|  |  | Lass4 | LAG1 homolog, ceramide synthase 4 | NM_001107117 | 0.42 | 0.008 | 0.47 | 0.029 |
|  |  | Actn2 | Actinin alpha 2 | NM_001170325 | 0.48 | 0.001 | 0.44 | 0.032 |
|  |  | Atp13a2 | Atpase type 13A2 | NM_001173432 | 0.44 | 0.013 | 0.5 | 0.043 |
|  |  | Eno2 | Enolase 2, gamma, neuronal | NM_139325 | 0.48 | 0.006 | 0.48 | 0.041 |
|  | | | | | | | | |
| **Signaling Pathway** | **Up-regulation** | **Gene Symbol** | **Gene name** | **Accession Number** | **Fold Change 7h** | ***p*-value 7h** | **Fold Change 24h** | ***p*-value 24h** |
|  |  | Il1rl1 | Interleukin 1 receptor-like 1 | NM_013037 | 13.01 | 0.03 | 47.02 | 0.001 |
|  |  | Gal | Galanin prepropeptide | NM_033237 | 9.39 | 0.004 | 5.6 | 0.029 |
|  |  | Postn | Periostin, osteoblast specific factor | NM_001108550 | 2.31 | 0.04 | 7.35 | 0.013 |
|  |  | Epha2 | Eph receptor A2 | NM_001108977 | 2.91 | 0.023 | 6.39 | 0.026 |
|  |  | Clcf1 | Cardiotrophin-like cytokine factor 1 | NM_207615 | 2.13 | 0.007 | 6.53 | 0.028 |
|  |  | Ripk3 | Receptor-interacting serine-threonine kinase 3 | NM_139342 | 2.98 | 0.049 | 5.02 | 0.025 |
|  |  | Runx2 | Runt-related transcription factor 2 | NM_053470 | 2.86 | 0,000 | 2.98 | 0.049 |
|  |  | Cd44 | Cd44 molecule | NM_012924 | 2.2 | 0.006 | 3.32 | 0.036 |
|  |  | Il1rn | Interleukin 1 receptor antagonist | NM_022194 | 2.79 | 0.001 | 2.2 | 0.024 |
|  |  | Tgif1 | TGFB-induced factor homeobox 1 | NM_001015020 | 2.11 | 0.041 | 2.73 | 0.04 |
|  |  | Lbxcor1 | LBXCOR1 homolog (mouse) | XM_002727063 | 2.35 | 0.042 | 2.49 | 0.024 |
|  |  | | | | | | | |
|  | **Down-regulation** | **Gene Symbol** | **Gene name** | **Accession Number** | **Fold Change 7h** | ***p*-value 7h** | **Fold Change 24h** | ***p*-value 24h** |
|  |  | Rasgrp1 | RAS guanyl releasing protein 1 (calcium and DAG-regulated) | NM_019211 | 0.25 | 0.003 | 0.3 | 0.034 |
|  |  | Loc100360071 | Neuropeptide S-like | ENSRNOT00000054896 | 0.19 | 0,000 | 0.38 | 0.05 |
|  |  | Chrm1 | Cholinergic receptor, muscarinic 1 | NM_080773 | 0.43 | 0.016 | 0.36 | 0.04 |
|  |  | Acvr1c | Activin A receptor, type IC | NM_139090 | 0.49 | 0.023 | 0.3 | 0.043 |
|  |  | Gpr26 | G protein-coupled receptor 26 | NM_138841 | 0.48 | 0.013 | 0.33 | 0.03 |
|  |  | Pml | Promyelocytic leukemia | XM_236296 | 0.43 | 0.008 | 0.44 | 0.036 |
|  | | | | | | | | |
| **Synapse** | **Up-regulation** | **Gene Symbol** | **Gene name** | **Accession Number** | **Fold Change 7h** | ***p*-value 7h** | **Fold Change 24h** | ***p*-value 24h** |
|  |  | Sypl2 | Synaptophysin-like 2 | NM_013037 | 13.01 | 0.03 | 47.02 | 0.001 |
|  |  | | | | | | | |
|  | **Down-regulation** | **Gene Symbol** | **Gene name** | **Accession Number** | **Fold Change 7h** | ***p*-value 7h** | **Fold Change 24h** | ***p*-value 24h** |
|  |  | Chrm1 | Cholinergic receptor, muscarinic 1 | NM_080773 | 0.43 | 0.016 | 0.36 | 0.04 |
|  |  | Clstn3 | Calsyntenin 3 | NM_134376 | 0.45 | 0.014 | 0.39 | 0.036 |
|  |  | Dlgap2 | Discs, large (Drosophila) homolog-associated protein 2 | NM_053901 | 0.48 | 0.011 | 0.4 | 0.036 |
|  | | | | | | | | |
| **Transcription** | **Up-regulation** | **Gene Symbol** | **Gene name** | **Accession Number** | **Fold Change 7h** | ***p*-value 7h** | **Fold Change 24h** | ***p*-value 24h** |
|  |  | Tgif1 | TGFB-induced factor homeobox 1 | NM_013037 | 13.01 | 0.03 | 47.02 | 0.001 |
|  |  | Runx2 | Runt-related transcription factor 2 | NM_053470 | 2.86 | 0,000 | 2.98 | 0.049 |
|  |  | Hopx | HOP homeobox | NM_133621 | 2.08 | 0.032 | 3.47 | 0.037 |
|  |  | Lbxcor1 | LBXCOR1 homolog (mouse) | XM_002727063 | 2.35 | 0.042 | 2.49 | 0.024 |
|  |  | | | | | | | |
|  | **Down-regulation** | **Gene Symbol** | **Gene name** | **Accession Number** | **Fold Change 7h** | ***p*-value 7h** | **Fold Change 24h** | ***p*-value 24h** |
|  |  | Pml | Promyelocytic leukemia | XM_236296 | 0.43 | 0.008 | 0.44 | 0.036 |
|  |  | Lass4 | LAG1 homolog, ceramide synthase 4 | NM_001107117 | 0.42 | 0.008 | 0.47 | 0.029 |
|  |  | Actn2 | Actinin alpha 2 | NM_001170325 | 0.48 | 0.001 | 0.44 | 0.032 |
|  | | | | | | | | |
| **Receptor Activity** | **Up-regulation** | **Gene Symbol** | **Gene name** | **Accession Number** | **Fold Change 7h** | ***p*-value 7h** | **Fold Change 24h** | ***p*-value 24h** |
|  |  | Tas2r120 | Taste receptor, type 2, member 120 | NM_001080937 | 3.91 | 0.047 | 6.21 | 0.008 |
|  |  | Il1rl1 | Interleukin 1 receptor-like 1 | NM_013037 | 13.01 | 0.03 | 47.02 | 0.001 |
|  |  | Cd93 | CD93 molecule | NM_053383 | 4.77 | 0,000 | 6.84 | 0.008 |
|  |  | Epha2 | Eph receptor A2 | NM_001108977 | 2.91 | 0.023 | 6.39 | 0.026 |
|  |  | Procr | Protein C receptor, endothelial | NM_001025733 | 3.66 | 0.027 | 5.49 | 0.039 |
|  |  | Gpr4 | G protein-coupled receptor 4 | NM_001025680 | 3.24 | 0.01 | 3.72 | 0.035 |
|  |  | Cd44 | Cd44 molecule | NM_012924 | 2.2 | 0.006 | 3.32 | 0.036 |
|  |  | Il1rn | Interleukin 1 receptor antagonist | NM_022194 | 2.79 | 0.001 | 2.2 | 0.024 |
|  |  | | | | | | | |
|  | **Down-regulation** | **Gene Symbol** | **Gene name** | **Accession Number** | **Fold Change 7h** | ***p*-value 7h** | **Fold Change 24h** | ***p*-value 24h** |
|  |  | Chrm1 | Cholinergic receptor, muscarinic 1 | NM_080773 | 0.43 | 0.016 | 0.36 | 0.04 |
|  |  | Acvr1c | Activin A receptor, type IC | NM_139090 | 0.49 | 0.023 | 0.3 | 0.043 |
|  |  | Gpr26 | G protein-coupled receptor 26 | NM_138841 | 0.48 | 0.013 | 0.33 | 0.03 |
|  | | | | | | | | |
| **Glutamate Secretion** | **Up-regulated** | **Gene Symbol** | **Gene name** | **Accession Number** | **Fold Change 7h** | ***p*-value 7h** | **Fold Change 24h** | ***p*-value 24h** |
|  |  | Il1rn | Interleukin 1 receptor antagonist | NM_022194 | 2.79 | 0.001 | 2.2 | 0.024 |
|  | | | | | | | | |
| **RNA Biosynthetic Process** | **Up-regulation** | **Gene Symbol** | **Gene name** | **Accession Number** | **Fold Change 7h** | ***p*-value 7h** | **Fold Change 24h** | ***p*-value 24h** |
|  |  | Hopx | HOP homeobox | NM_013037 | 13.01 | 0.03 | 47.02 | 0.001 |
|  |  | Runx2 | Runt-related transcription factor 2 | NM_053470 | 2.86 | 0,000 | 2.98 | 0.049 |
|  |  | Tgif1 | TGFB-induced factor homeobox 1 | NM_001015020 | 2.11 | 0.041 | 2.73 | 0.04 |
|  |  | | | | | | | |
|  | **Down-regulation** | **Gene Symbol** | **Gene name** | **Accession Number** | **Fold Change 7h** | ***p*-value 7h** | **Fold Change 24h** | ***p*-value 24h** |
|  |  | Lass4 | LAG1 homolog, ceramide synthase 4 | NM_001107117 | 0.42 | 0.008 | 0.47 | 0.029 |
|  | | | | | | | | |
| **Response to Oxidative Stress** | **Up-regulation** | **Gene Symbol** | **Gene name** | **Accession Number** | **Fold Change 7h** | ***p*-value 7h** | **Fold Change 24h** | ***p*-value 24h** |
|  |  | Gpx2 | Glutathione peroxidase 2 | NM_013037 | 13.01 | 0.03 | 47.02 | 0.001 |
|  |  | Serpine1 | Serpin peptidase inhibitor, clade E (nexin, plasminogen activator inhibitor type 1), member 1 | NM_012620 | 5.03 | 0.018 | 9.77 | 0.028 |
|  |  | | | | | | | |
|  | **Down-regulation** | **Gene Symbol** | **Gene name** | **Accession Number** | **Fold Change 7h** | ***p*-value 7h** | **Fold Change 24h** | ***p*-value 24h** |
|  |  | Pml | Promyelocytic leukemia | XM_236296 | 0.43 | 0.008 | 0.44 | 0.036 |
