# Supplementary material for: In Vitro Ischemia Triggers a Transcriptional Response to Down-Regulate Synaptic Proteins in Hippocampal Neurons
Source: PLoS One. 2014 Jun 24;9(6):e99958. doi: 10.1371/journal.pone.0099958 (PMC4069008; doi:10.1371/journal.pone.0099958)
Supplement: Table S8 — Microarray data of the genes selected for validation through qPCR analyses. (DOCX) [file pone.0099958.s009.docx]

|  | **Gene Name** | **Function** | **Status in microarrays** | **Fold change in microarrays** |
| --- | --- | --- | --- | --- |
| **Prkcε** | Protein kinase C epsilon | Protein kinase C | Down-regulated at 24h after OGD | 0.46 |
| **Prkcδ** | Protein kinase C delta | Protein kinase C | Up-regulated at 24h after OGD | 2.20 |
| **Mmp25** | Matrix metallopeptidase 25 | Extracelular matrix protease | Up-regulated at 24h after OGD | 2.27 |
| **Mmp3** | Matrix metallopeptidase 3 | Extracelular matrix protease | Up-regulated at 24h after OGD | 2.93 |
| **Adamts5** | ADAM metallopeptidase with thrombospondin type 1 motif 5 | Extracelular matrix protease | Up-regulated at 24h after OGD | 2.24 |
| **Adamts7** | ADAM metallopeptidase with thrombospondin type 1 motif 7 | Extracelular matrix protease | Up-regulated at 7h and 24h after OGD | 2.28 (7h) / 2.64 (24h) |
| **Batf3** | Basic leucine zipper transcription factor, ATF-like 3 | Transcription factor | Up-regulated at 7h after OGD | 2.94 |
| **Hmgb1** | High mobility group box 1 | Transcription factor | Up-regulated at 7h after OGD | 2.00 |
| **Nfil3** | Nuclear factor, interleukin 3 regulated | Transcription factor | Down-regulated at 24h after OGD | 0.44 |
| **Itgb6** | Integrin beta 6 | Cell adhesion | Up-regulated at 7h after OGD | 3.74 |
| **Gadd45g** | Growth arrest and DNA-damage-inducible, gamma | Cell death | Up-regulated at 7h after OGD | 2.08 |
